# Pt(II) Complexes with Tetradentate C^N*N^C Luminophores: From Supramolecular Interactions to Temperature-Sensing Materials with Memory and Optical Readouts

**DOI:** 10.3390/molecules28217353

**Published:** 2023-10-30

**Authors:** Matias E. Gutierrez Suburu, Meik Blanke, Alexander Hepp, Oliver Maus, Dominik Schwab, Nikos L. Doltsinis, Wolfgang G. Zeier, Michael Giese, Jens Voskuhl, Cristian A. Strassert

**Affiliations:** 1 Institut für Anorganische und Analytische Chemie, Universität Münster, Corrensstraße 28/30, D-48149 Münster, Germany; 2 Center of Nanotechnology (CeNTech), Center for Soft Nanosciences (SoN), Cells in Motion Interfaculty Cluster (CiMIC), Universität Münster, Heisenbergstraße 11, D-48149 Munster, Germany; 3 Center for Nanointegration Duisburg-Essen (CENIDE), Faculty of Chemistry (Organic Chemistry), University of Duisburg-Essen, Universitätsstraße 7, D-45141 Essen, Germany; 4 Center for Multiscale Theory and Computation, Institut für Festkörpertheorie, Universität Münster, Wilhelm-Klemm-Straße 10, D-48149 Münster, Germany

**Keywords:** Pt(II) complex, organometallic compounds, phosphorescent complexes, thermochromism

## Abstract

A series of four regioisomeric Pt(II) complexes (**PtL^a^-n** and **PtL^b^-n**) bearing tetradentate luminophores as dianionic ligands were synthesized. Hence, both classes of cyclometallating chelators were decorated with three *n*-hexyl (*n* = 6) or *n*-dodecyl (*n* = 12) chains. The new compounds were unambiguously characterized by means of multiple NMR spectroscopies and mass spectrometry. Steady-state and time-resolved photoluminescence spectroscopy as well quantum chemical calculations show that the effect of the regioisomerism on the emission colour and on the deactivation rate constants can be correlated with the participation of the Pt atom on the excited state. The thermal properties of the complexes were studied by DSC, POM and temperature-dependent steady-state photoluminescence spectroscopy. Three of the four complexes (**PtL^a^-12**, **PtL^b^-6** and **PtL^b^-12**) present an intriguing thermochromism resulting from the responsive metal–metal interactions involving adjacent monomeric units. Each material has different transition temperatures and memory capabilities, which can be tuned at the intermolecular level. Hence, dipole–dipole interactions between the luminophores and disruption of the crystalline packing by the alkyl groups are responsible for the final properties of the resulting materials.

## 1. Introduction

Luminescent Pt(II) complexes have been widely studied as optical sensors for both physical (mechanic [1,2,3,4], temperature [5,6,7], polarity [6]) and chemical (vapours [2,8,9], ions [10,11], small molecules [12], molecular oxygen [5,13,14]) stimuli. Moreover, several patents on their use in optoelectronic devices and as sensors have been applied for [15,16,17,18]. The phosphorescence of Pt(II) complexes, which is widely discussed in the bibliographic literature, generally stems from metal-perturbed ligand-centred triplet states (^3^MP-LC), where the emissive species possesses a mixed ^3^LC (ligand-centred, i.e., π-π*) and ^3^MLCT (metal-to-ligand charge transfer, i.e., d-π*) character [19,20,21,22]. There are numerous examples of Pt(II) complexes with both bidentate and tridentate ligands that are flexible enough to allow for geometrical distortion out of the coordination plane, which upon photoexcitation facilitates non-radiative decay pathways through conical intersections with the ground state [23,24]. Hence, the use of tetradentate ligands can increase the rigidity and, in consequence, enhance the photoluminescence efficiency of the complexes [25,26]. Moreover, cyclometallation can be further used to enhance the ligand-field splitting by the introduction of strong *σ*-donors, raising the antibonding *d*_x_^2^_-y_^2^ orbital to higher energies and, in consequence, destabilizing dissociative states (including d-d* and π-d* configurations), which in turn become less thermally accessible [26,27,28,29,30].

Several Pt(II)-based species with sensing applications rely on their capability to form aggregates, both in solution and in the solid state. The formation of Pt(II)-based aggregates is possible due to their planar coordination geometry and is mainly driven by *van der Waals* interactions. When the distance between the Pt atoms is approximately 3.5 Å or shorter (i.e., below the sum of their *van der Waals* radii), coupling between the *d*_z_^2^ orbital lobes protruding out of the coordination plane becomes feasible. The resulting aggregates show a red-shifted emission if compared with the monomeric species, which arises from triplet metal–metal-to-ligand charge transfer (^3^MMLCT) states with a certain degree of excimeric character [14,21,31,32]. Hence, we have now prepared a series of Pt(II) complexes where the modification in the length and position of peripheral alkyl chains allows us to control the photophysical properties, the characteristics of the solid phase and the transition temperatures of the resulting solids. These concepts can lead to temperature-sensing materials with memory based on Pt-Pt interactions and providing optical readouts.

The complexes presented in this work have been developed as a natural progression of our previous research [4]. The herein-reported replacement of the methoxyphenyl group at the bridging N-atom by alkyl chains aims at promoting the aggregation of the complexes. This stacking was partially hampered in our previous complexes, due to the 90° rotation of the phenyl ring with respect to the luminophoric plane [4,14]. In addition, modulation of the length and position of the alkyl chains affects the packing and supramolecular interactions between the molecular units in the solid state, leading to changes in the thermal properties of the complexes.

## 2. Synthesis

**Scheme 1:**
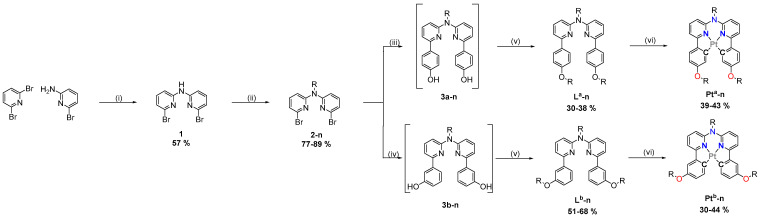
Synthesis of the complexes. (i) NaH/DMF; (ii) alkylbromide, NaH /DMF; (iii) 4-hydroxyphenylboronic acid, K_2_CO_3_, Pd(PPh_3_)_4_/THF; (iv) 3-hydroxyphenylboronic acid, K_2_CO_3_, Pd(PPh_3_)_4_/THF; (v) alkylbromide, K_2_CO_3_/butanone; (vi) K_2_PtCl_4_/acetic acid.

The synthesis of the ligands (Scheme 1) started with the reaction between 2,6-dibromopyridine and 2-amino-6-bromopyridine promoted by NaH to give the precursor **1**. The intermediate **1** was reacted with 1-bromohexane or 1-bromododecane to yield the precursors **2-6** and **2-12**, respectively. A further Suzuki–Miyaura cross-coupling with an excess of the corresponding hydroxyphenylboronic acid allowed us to prepare the intermediates **3a-n** and **3b-n** (*n* = 6 or 12). The phenolic species were not isolated and the ligands **L^a^-n** and **L^b^-n** were finally obtained by Williamson’s ether synthesis using the corresponding alkylbromide [4]. The Pt(II) complexes were prepared by a standard cycloplatination reaction with K_2_PtCl_4_ in acetic acid at reflux with yields between 30 and 44%, which are in agreement with previously reported syntheses [4,14]. The comparable yields obtained for both regioisomers suggest that, in the case of **PtL^b^-n** complexes, only one of the three potential cyclometallation products is formed. This result may be attributed to the steric hindrance imposed by the alkyl chains during the cycloplatination reaction. The complexes show a good solubility in polar organic solvents, such as DCM and THF, mostly due to the presence of the peripheral alkyl chains [4]. All the precursors and complexes were unambiguously characterized by means of 2D-NMR (including a full assignment of ^1^H and ^13^C signals) as well as by high-resolution mass spectrometry (Figures S1–S61). Further experimental details can be found in the ESI.

## 3. Photophysics in Diluted Conditions

**Figure 1:**
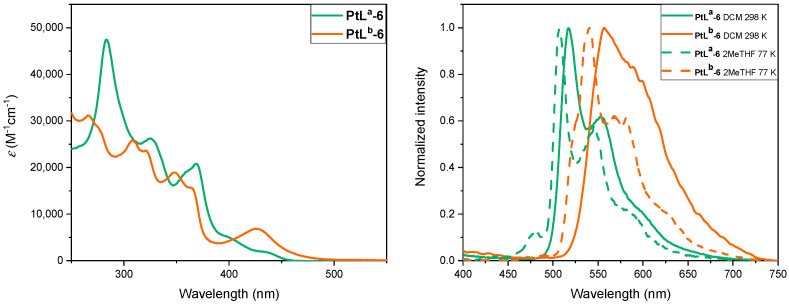
Absorption (**left**) and emission spectra (*λ*_exc_ = 350 nm, **right**) of **Pt^a^-6** (green) and **Pt^b^-6** (orange) in fluid DCM solution at r.t. (solid lines), in 2-MeTHF glassy matrices at 77 K (dashed lines) and in PMMA films at r.t. (dotted lines).

The photophysical characterization of the complexes was initially performed in diluted DCM solutions (complex concentration *c* ≈ 10^−5^ M) at room temperature. The absorption bands in the region between 235 and 300 nm can be assigned to transitions into ^1^LC states, while the bands in the region between 350 and 450 nm are related to mixed singlet states with variable degrees of LC and MLCT character, as was previously reported for comparable complexes (Figure 1 left, Figure S62 and Table S1) [14,21,33,34,35]. Interestingly, the **PtL^b^-n** complexes present a stronger absorption band in the ^1^MLCT (≈420 nm) region if compared with the **PtL^a^-n** complexes. This can be attributed to the higher contribution of the alkoxy group in *para* position (with respect to the Pt atom) to the HOMO than in case of the moiety in *meta* position, and therefore to a higher destabilization of the HOMO (as previously observed for Ir(III) complexes and [36,37] in phenylpyridine Pt(II)-based complexes [38]).

The emission spectra of the **PtL^a^-n** complexes (Figure 1 right, Figure S63) show a maximum at 517 nm with vibrational shoulders at 553 nm, which arise mainly from ^3^MP-LC states [14,33,34,39]. The spectra of **PtL^b^-n** are red-shifted with a maximum at 555 nm (Figure 1 right, Figure S64). The red shift in the emission can be also ascribed to a destabilization of the HOMO and consequently to a reduction in the gap between the excited and ground state [37,38]. To support and interpret the experimental results, (TD-)DFT calculations were performed for the methoxy-substituted complexes (**PtL^a^-1** and **PtL^b^-1**) as model compounds. After the geometry optimization of the ground state (S_0_) and the lowest triplet state (T_1_) was achieved, the emission maxima were calculated (Figure S77) [40,41,42,43,44,45,46,47].

**Figure 2:**
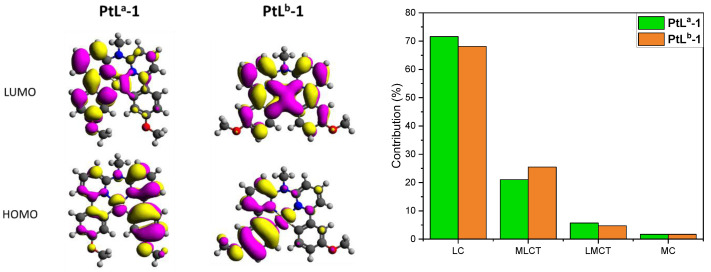
Molecular orbitals dominating the T_1_ state of **PtL^a^-1** and **PtL^b^-1** at the optimized T_1_ geometry (**left**). Characterization of the emissive T_1_ state by correlated electron–hole pair analysis (**right**).

A detailed characterization of the emissive state can be achieved with the aid of a correlated electron-hole pair analysis using the software package for Theoretical Density, Orbital Relaxation and Exciton analysis (TheoDORE) [48]. The result of the decomposition into contributions from MLCT, LMCT (ligand-to-metal charge transfer), LC and MC is presented in Figure 2. The higher MLCT character in the excited state of **PtL^b^-n**, particularly if compared with **PtL^a^-n** (25.0% vs 21.1%, respectively), may be responsible for the lack of vibronic progression in **PtL^b^-n** complexes, due the stronger geometrical distortion in the excited state. The distortion of the excited state (Δ*Q*) can be estimated by the so-called Huang–Rhys factor (*S*) according to Equation (1), where *I*_0-0_ is the emission intensity of the 0-0 transition and *I*_1-0_ the one corresponding to the first vibronic peak (Table 1) [26]. Consistently, *S* is higher for **PtL^b^-n** than for **PtL^a^-n** complexes.

(1) $$S=\frac{I_{0-1}}{I_{0-0}}$$

The photophysical properties were also studied in diluted glassy matrices of 2-methyltetrahydrofuran (complex concentration, *c* ≈ 10^−5^ M) at 77 K. All the complexes show a blue shift in the emission spectra of approximately 10 nm, if compared with the solutions at r.t. This is a consequence of the weaker charge-transfer stabilization associated with the limited solvent reorientation in the rigid matrices, thereby decreasing the ^3^MLCT character of the emissive state [49,50]. Another consequence of the restricted solvent reorientation is the reduced density of solvent-related roto-vibrational states, thus leading to a well-defined vibrational progression [51]. Interestingly, the reduction in *S* is more significant for **PtL^b^-n** than for **PtL^a^-n** when the fluid solution is changed to the glassy matrix, which is in agreement with a higher loss of MLCT character from the lack of solvent reorientation.

The excited state of Pt(II) complexes can be deactivated in fluid solution by ^3^O_2_-mediated diffusional quenching, which is reflected in the decrease (by a factor > 50) in the *k*_nr_ after Ar purging of the samples. The **PtL^a^-n** series shows longer lifetimes (Figures S65–S76) and higher quantum yields than their **PtL^b^-n** isomers (Table 2); a similar effect was previously reported for Ir(III) complexes [37].

When the complexes are studied in dilute fluid solutions, different conformations are thermally accessible and possess comparable excited state characters yet different deactivation rates; therefore, multiexponential decays can be observed in some cases. This effect is also noticeable in glassy matrices, yielding multiexponential decays as previously reported for other Pt(II) complexes [33,51,52]. Compared to the analogous complex with a methoxyphenyl group (which has been previously reported by our group [4]), **PL^a^-6** presents somewhat higher *k*_r_ and *k*_nr_ rates. This can be attributed to the reduced steric strain associated with the *n*-alkyl substitution, as opposed to a phenyl moiety at the bridging N-atom. The *k*_r_ and *k*_nr_ values of **PtL^a^-n** and **PtL^b^-n** show two interesting trends. The *k*_r_ are in the same range for both luminophores; however, **PtL^b^-n** complexes present *k*_nr_ values between 2 and 3 times higher than the **PtL^a^-n** compounds. In this case, the faster non-radiative intersystem crossing rate constant between the T_1_ and S_0_ states can be ascribed to the higher geometrical distortion in the excited state of **PtL^b^-n**, if compared with the **PtL^a^-n** complexes. It would be expected that a lower MLCT character of the excited state leads to a higher *k*_r_ at 77 K than at r.t. However, the four complexes show a counterintuitive tendency, with higher *k*_r_ at lower temperatures. This was already observed for other Pt(II) complexes with tridentate luminophores, and (TD-)DFT calculations at a higher level of sophistication (including a temperature-dependent *Boltzmann* analysis of the radiative rate constants) will be needed to elucidate the nature of this observation [53].

**Table 2:** Photophysical parameters of the complexes.

	PtL^a^-6				PtL^a^-12				PtL^b^-6				PtL^b^-12			
	*τ*_av_ ^a^ (µs)	*Φ*_L_ ^b^	*k*_r_ ^c^/10^4^ (s^−1^)	*k*_nr_ ^d^/10^4^ (s^−1^)	*τ*_av_ ^a^ (µs)	*Φ*_L_ ^b^	*k*_r_ ^c^/10^4^ (s^−1^)	*k*_nr_ ^d^/10^4^ (s^−1^)	*τ*_av_ ^a^ (µs)	*Φ*_L_ ^b^	*k*_r_ ^c^/10^4^ (s^−1^)	*k*_nr_ ^d^/10^4^ (s^−1^)	*τ*_av_ ^a^ (µs)	*Φ*_L_ ^b^	*k*_r_ ^c^/10^4^ (s^−1^)	*k*_nr_ ^d^/10^4^ (s^−1^)
Air-equilibrated DCM solution (r.t.)	0.203 ± 0.001 ^e^	<0.02	<10	480 < *k*_nr_ < 490	0.174 ± 0.001 ^e^	<0.02	<12	560 < *k*_nr_ < 570	0.163 ± 0.001 ^e^	<0.02	<12	600 < *k*_nr_ < 610	0.209 ± 0.001 ^f^	<0.02	<10	470 < *k*_nr_ < 480
Ar-purged DCM solution (r.t.)	11.22 ± 0.02 ^e^	0.49 ± 0.02	4.4 ± 0.2	4.5 ± 0.2	10.60 ± 0.02 ^f^	0.57 ± 0.02	5.4 ± 0.2	4.1 ± 0.2	6.102 ± 0.003 ^e^	0.26 ± 0.02	4.3 ± 0.3	12.1 ± 0.3	8.55 ± 0.03 ^f^	0.27 ± 0.02	3.2 ± 0.2	8.5 ± 0.3
2-MeTHF glassy matrix (77 K)	16.8 ± 0.1 ^e^	0.99 ± 0.01	6.0 ± 0.2	<6	11.4 ± 0.8 ^f^	0.99 ± 0.01	8.8 ± 0.8	<9	7.60 ± 0.03 ^f^	0.99 ± 0.01	13.2 ± 0.3	<13	8.7 ± 0.1 ^f^	0.99 ± 0.01	11.6 ± 0.4	<11

^a^ Amplitude-weighted average lifetime. ^b^ Photoluminescence quantum yield. ^c^ Average radiative rate constant $k_{r}=\frac{\Phi_{L}}{\tau_{av}}$; ${∆k}_{r}=\left(\frac{{∆\Phi }_{L}}{\tau_{av}}\right)+\left(\frac{\Phi_{L}}{{\tau_{av}}^{2}}\;∆\tau_{av}\right)$ [54]. ^d^ Average radiationless deactivation rate constant $k_{nr}=\frac{1-\Phi_{L}}{\tau_{av}}$; ${∆k}_{nr}=\left(\frac{{∆\Phi }_{L}}{\tau_{av}}\right)+\left(\frac{∆\tau_{av}}{{\tau_{av}}^{2}}\;\right)+\left(\frac{\Phi_{L}}{{\tau_{av}}^{2}}\;∆\tau_{av}\right)$ [54]. ^e^ Monoexponential decay. ^f^ Biexponential decay.

## 4. Thermochromic Properties

The thermochromic properties of the compounds were studied by differential scanning calorimetry (DSC), polarized optic microscopy (POM) and temperature-dependent steady-state luminescence spectroscopy.

In DSC experiments (Table 3, Figures S78–S81), **PtL^a^-6** melts at 245 °C with decomposition. No changes below this temperature were observed in POM. **PtL^a^-12** shows, within a first cycle, a crystal-to-crystal transition at 74 °C, while melting at 197 °C and crystallizing again at 186 °C (Figure S82). Changes in the melting temperature within cycles for **PtL^a^-12** indicate a small decomposition during the phase transition. **PtL^b^-6** and **PtL^b^-12** both melt at around 130 °C, returning not to a crystalline phase, but rather to a glassy phase in a reversible way (Figures S83 and S84). In the case of the “open” series **PtL^b^-n**, the increasing chain length produces an enhancement of the *van der Waals* interactions between the alkyl groups while causing a slight raise of the melting temperature and a significant increment of the melting enthalpy.

**Figure 3:**
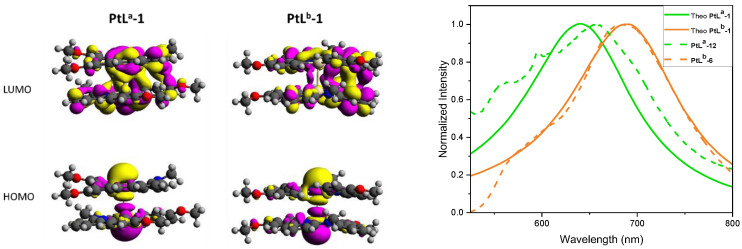
Molecular orbitals dominating the description of the T_1_ state for the dimers of **PtL^a^-1 A** (**left**) and **PtL^b^-1 B** (**right**) at the optimized T_1_ geometry (**left**). Calculated (solid lines) and experimental (dashed lines) emission spectra of the aggregates (**right**).

Interestingly, the transitions undergone by **PtL^a^-12** involve a change in the luminescence colour, with a decrease of the monomeric emission and the growth of an unstructured yet red-shifted band. This new luminescence can be ascribed to the formation of dimers and aggregates where the Pt···Pt distances are shorter than 3.5 Å. [3,14,34,55]. The assignment of this emission band was carried out by comparing the calculated emission spectra of the dimers with the experimental bands measured upon heating of the samples. The calculated emission spectra of **PtL^a^-1** and **PtL^b^-1** dimers are in very good agreement with the experimental data (Figure 3).

**Figure 4:**
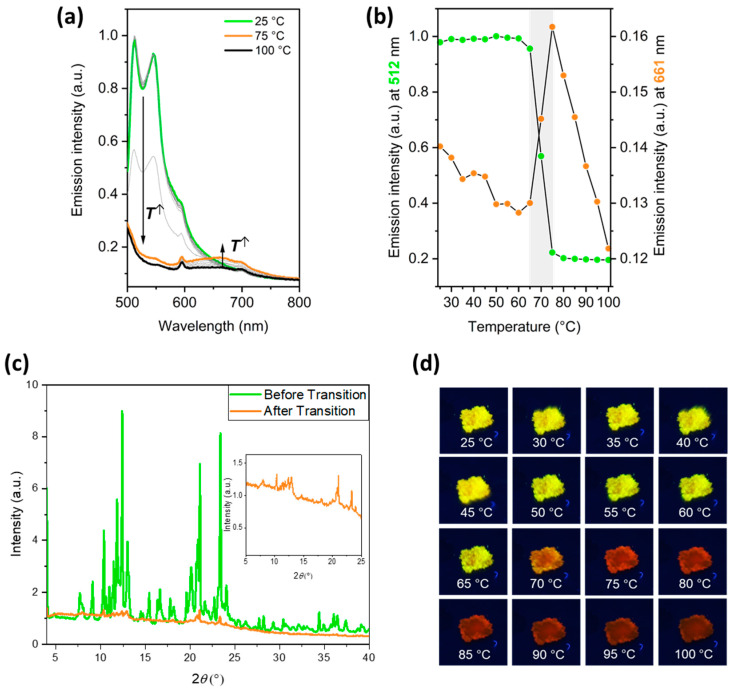
(**a**) Emission spectra of solid **PtL^a^-12** upon heating (*λ*_exc_ = 470 nm). (**b**) Emission intensity at 512 nm and 661 nm as a function of the temperature. (**c**) X-ray diffraction pattern of **PtL^a^-12** before and after the phase transition. Inset: detail of the X-ray diffraction pattern after the phase transition. (**d**) Photographs of solid **PtL^a^-12** at different temperatures under UV-light excitation, *λ*_exc_ = 365 nm.

Powder X-ray diffractometry of **PtL^a^-12** was performed before and after heating the sample above the transition temperature. The diffraction pattern before the thermal treatment shows typical *Bragg* reflections of a polycrystalline sample. Upon heating, no *Bragg* reflections are observed while showing the formation of a mostly amorphous solid (Figure 4).

In the case of complexes **PtL^b^-6** and **PtL^b^-12**, the monomeric emission intensities decrease with increasing temperature (Figures S86 and S87), as expected for the progressive population of roto-vibrational states while leading to faster non-radiative deactivation rates. On the other hand, **PtL^a^-12** shows a very stable emission intensity at 512 nm as a function of temperature below the transition temperature. In the range between 65 °C and 75 °C, the emission intensity at 512 nm decreases by roughly 80%.

**Figure 5:**
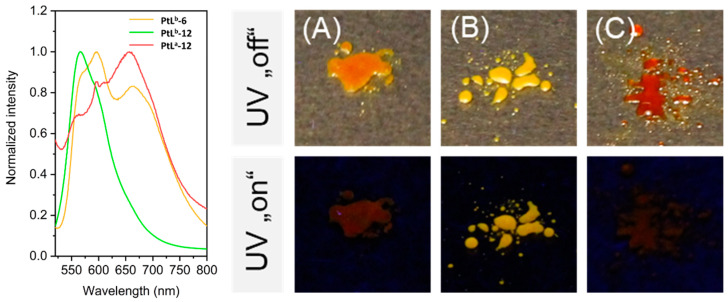
Emission spectra of **PtL^a^-12** (red), **PtL^b^-12** (green) and **PtL^b^-6** (orange) after melting and 48 h storage under ambient conditions (**left**). Photographs of the complexes **PtL^a^-12** (**A**), **PtL^b^-12** (**B**) and **PtL^b^-6** (**C**) after melting and 48 h storage under ambient conditions as observed under room light and under UV-light irradiation, *λ*_exc_ = 365 nm (**right**).

The emission spectra of **PtL^a^-12**, **PtL^b^-6** and **PtL^b^-12** were measured 48 h after melting (Figure 5). Notably, each complex exhibits a different behaviour. **PtL^a^-12** keeps the emission of the aggregates kinetically blocked and **PtL^b^-6** shows partial recovery, whereas for **PtL^b^-12** the original emission (i.e., before heating) is fully recovered. These observations can be explained in terms of the intermolecular interactions within the solids: the “closed” complexes **PtL^a^-n** possess a higher dipolar moment than the “open” **PtL^b^-n** series. Consequently, the interaction between the aromatic cores within the aggregates is more pronounced. Conversely, the longer alkyl moieties disrupt the interactions between the aromatic planes [56,57]. As a result of these combined effects, **PtL^b^-12** is able to return to a monomer-dominated phase, whereas **PtL^b^-6** achieves only a partial reversion. Ultimately, the more robust dipole interactions sustain the aggregates in the case of **PtL^a^-12**.

## 5. Conclusions

Four new Pt(II) complexes with C^N*N^C ligands were synthesized and characterized. The position of the alkoxy groups has a crucial effect on the photophysical properties of the monomeric complexes by varying the metal participation on the excited state. The **PtL^b^-n** complexes with an extended metal contribution display emission maxima that are red-shifted by approximately 45 nm while having higher *k*_nr_ values in comparison with their **PtL^a^-n** isomers; this demonstrates that small changes in the ligand structure can significantly affect the photophysical properties. The alkyl chain length affects the capability of the complexes to form crystalline phases and their transition temperatures. Interestingly, the phase transitions facilitate the Pt-Pt coupling, which causes a red-shifted luminescence due to the formation of dimers. Depending on the substitution pattern and the alkyl chain length, the thermal properties of the materials can be tuned. Moreover, the balance between the different classes of intermolecular interactions affects the memory of thermal exposure above the transition temperatures. Hence, variable alkyl chain lengths can be exploited to design materials with different sensitivity ranges, in order to cover appropriated windows for their use as sensors with multiple optical readouts (i.e., based on photoluminescence wavelengths, intensities and lifetimes).

## Figures and Tables

**Table 1:** Huang–Rhys factor in fluid solution at r.t. and in glassy matrices at 77 K for the four complexes.

Complex	Huang–Rhys Factor *S*	
	Fluid Solution at r.t.	Glassy Matrix at 77 K
**PtL^a^-6**	0.61	0.57
**PtL^a^-12**	0.66	0.46
**PtL^b^-6**	0.83	0.61
**PtL^b^-12**	0.80	0.54

**Table 3:** Transition temperatures and enthalpies of the complexes.

	Phase Transitions	
*n*	6	12
**PtL^a^-n**	Cr 245 I_decomp_	Cr 197 (22.3) I
**PtL^b^-n**	Cr 127 (30.8) I	Cr 132 (45.4) I

Temperatures given in °C; enthalpies in kJ·mol^−1^ are indicated in parentheses. Cr: crystalline phase; I: isotropic liquid. Heating rates: 10 °C/s. Values are reported in the second heating cycle except for **PL^a^-6**.

## Data Availability

Further data supporting the results can be found in the electronic supporting information.

## Supplementary Materials

The following supporting information can be downloaded at: https://www.mdpi.com/article/10.3390/molecules28217353/s1. Figure S1. ^1^H-NMR spectrum (500 MHz, CD_2_Cl_2_) of 1. Figure S2. ^13^C-{^1^H}-NMR spectrum (126 MHz, CD_2_Cl_2_) of 1. Figure S3. ESI-MS (MeOH) of 1. Figure S4. ^1^H-NMR spectrum (500 MHz, CD_2_Cl_2_) of 2-6. Figure S5. ^13^C-{^1^H}-NMR spectrum (126 MHz, CD_2_Cl_2_) of 2-6. Figure S6. ESI-MS (MeOH) of 2-6. Figure S7. ^1^H-NMR spectrum (500 MHz, CD_2_Cl_2_) of 2-12. Figure S8. ^13^C-{^1^H}-NMR spectrum (126 MHz, CD_2_Cl_2_) of 2-12. Figure S9. ESI-MS (MeOH) of 2-12. Figure S10. ^1^H-NMR spectrum (500 MHz, CD_2_Cl_2_) of L^a^-6. Figure S11. ^13^C-{^1^H}-NMR spectrum (126 MHz, CD_2_Cl_2_) of L^a^-6. Figure S12. ^1^H/^1^H-COSY-NMR spectrum (500 MHz/500 MHz, CD_2_Cl_2_) of L^a^-6. Figure S13. ^1^H/^13^C-gHSQC-NMR spectrum (500 MHz/126 MHZ, CD_2_Cl_2_) of L^a^-6. Figure S14. ^1^H/^13^C-gHMBC-NMR spectrum (500 MHz/126 MHz, CD_2_Cl_2_) of L^a^-6. Figure S15. ESI-MS (MeOH) of L^a^-6. Figure S16. ^1^H-NMR spectrum (500 MHz, CD_2_Cl_2_) of L^a^-12. Figure S17. ^13^C-{^1^H}-NMR spectrum (126 MHz, CD_2_Cl_2_) of L^a^-12. Figure S18. ^1^H/^1^H-COSY-NMR spectrum (500 MHz/500 MHz, CD_2_Cl_2_) of L^a^-12. Figure S19. ^1^H/^13^C-gHSQC-NMR spectrum (500 MHz/126 MHZ, CD_2_Cl_2_) of L^a^-12. Figure S20. ^1^H/^13^C-gHMBC-NMR spectrum (500 MHz/126 MHz, CD_2_Cl_2_) of L^a^-12. Figure S21. ESI-MS (MeOH) of L^a^-12. Figure S22. ^1^H-NMR spectrum (500 MHz, CD_2_Cl_2_) of L^b^-6. Figure S23. ^13^C-{^1^H}-NMR spectrum (126 MHz, CD_2_Cl_2_) of L^b^-6. Figure S24. ^1^H/^1^H-COSY-NMR spectrum (500 MHz/500 MHz, CD_2_Cl_2_) of L^b^-6. Figure S25. ^1^H/^13^C-gHSQC-NMR spectrum (500 MHz/126 MHZ, CD_2_Cl_2_) of L^b^-6. Figure S26. ^1^H/^13^C-gHMBC-NMR spectrum (500 MHz/126 MHz, CD_2_Cl_2_) of L^b^-6. Figure S27. ESI-MS (MeOH) of L^b^-6. Figure S28. ^1^H-NMR spectrum (400 MHz, CD_2_Cl_2_) of L^b^-12. Figure S29. ^13^C-{^1^H}-NMR spectrum (101 MHz, CD_2_Cl_2_) of L^b^-12. Figure S30. ^1^H/^1^H-COSY-NMR spectrum (400 MHz/400 MHz, CD_2_Cl_2_) of L^b^-12. Figure S31. ^1^H/^13^C-gHSQC-NMR spectrum (400 MHz/101 MHZ, CD_2_Cl_2_) of L^b^-12. Figure S32. ^1^H/^13^C-gHMBC-NMR spectrum (400 MHz/101 MHz, CD_2_Cl_2_) of L^b^-12. Figure S33. ESI-MS (MeOH) of L^b^-12. Figure S34. ^1^H-NMR spectrum (400 MHz, CD_2_Cl_2_) of PtL^a^-6. Figure S35. ^13^C-{^1^H}-NMR spectrum (101 MHz, CD_2_Cl_2_) of PtL^a^-6. Figure S36. ^195^Pt-NMR spectrum (86 MHz, CD_2_Cl_2_) of PtL^a^-6. Figure S37. ^1^H/^1^H-COSY-NMR spectrum (400 MHz/400 MHz, CD_2_Cl_2_) of PtL^a^-6. Figure S38. ^1^H/^13^C-gHSQC-NMR spectrum (400 MHz/101 MHZ, CD_2_Cl_2_) of PtL^a^-6. Figure S39. ^1^H/^13^C-gHMBC-NMR spectrum (400 MHz/101 MHz, CD_2_Cl_2_) of PtL^a^-6. Figure S40. ESI-MS (MeOH) of PtL^a^-16. Figure S41. ^1^H-NMR spectrum (500 MHz, CD_2_Cl_2_) of PtL^a^-12. Figure S42. ^13^C-{^1^H}-NMR spectrum (126 MHz, CD_2_Cl_2_) of PtL^a^-12. Figure S43. ^195^Pt-NMR spectrum (107 MHz, CD_2_Cl_2_) of PtL^a^-12. Figure S44. ^1^H/^1^H-COSY-NMR spectrum (500 MHz/500 MHz, CD_2_Cl_2_) of PtL^a^-12. Figure S45. ^1^H/^13^C-gHSQC-NMR spectrum (500 MHz/126 MHZ, CD_2_Cl_2_) of PtL^a^-12. Figure S46. ^1^H/^13^C-gHMBC-NMR spectrum (500 MHz/126 MHz, CD_2_Cl_2_) of PtL^a^-12. Figure S47. ESI-MS (MeOH) of PtL^a^-12. Figure S48. ^1^H-NMR spectrum (500 MHz, CD_2_Cl_2_) of PtL^b^-6. Figure S49. ^13^C-{^1^H}-NMR spectrum (126 MHz, CD_2_Cl_2_) of PtL^b^-6. Figure S50. ^195^Pt-NMR spectrum (107 MHz, CD_2_Cl_2_) of PtL^b^-6. Figure S51. ^1^H/^1^H-COSY-NMR spectrum (500 MHz/500 MHz, CD_2_Cl_2_) of PtL^b^-6. Figure S52. ^1^H/^13^C-gHSQC-NMR spectrum (500 MHz/126 MHZ, CD_2_Cl_2_) of PtL^b^-6. Figure S53. ^1^H/^13^C-gHMBC-NMR spectrum (500 MHz/126 MHz, CD_2_Cl_2_) of PtL^b^-6. Figure S54. ESI-MS (MeOH) of PtL^b^-6. Figure S55. ^1^H-NMR spectrum (500 MHz, CD_2_Cl_2_) of PtL^b^-12. Figure S56. ^13^C-{^1^H}-NMR spectrum (126 MHz, CD_2_Cl_2_) of PtL^b^-12. Figure S57. ^195^Pt-NMR spectrum (107 MHz, CD_2_Cl_2_) of PtL^a^-12. Figure S58. ^1^H/^1^H-COSY-NMR spectrum (500 MHz/500 MHz, CD_2_Cl_2_) of PtL^b^-12. Figure S59. ^1^H/^13^C-gHSQC-NMR spectrum (500 MHz/126 MHZ, CD_2_Cl_2_) of PtL^b^-12. Figure S60. ^1^H/^13^C-gHMBC-NMR spectrum (500 MHz/126 MHz, CD_2_Cl_2_) of PtL^b^-12. Figure S61. ESI-MS (MeOH) of PtL^b^-12. Figure S62. Absorption spectra of the four complexes in DCM at room temperature. Table S1. Absorption coefficients of the four complexes at selected wavelengths. Figure S63. Excitation (left, at the corresponding emission maximum) and emission (right, λ_exc_ = 350 nm) spectra of PtL^a^-6 and PtL^a^-12. Excitation (left, at the corresponding emission maximum) and emission (right, λ_exc_ = 350 nm) spectra of PtL^b^-6 and PtL^b^-12. Figure S65. Left: Raw time-resolved photoluminescence decay of PtL^a^-6 (c ≈ 10^−5^ M) in fluid air-equilibrated DCM at r.t., including the residuals (λ_exc_ = 376.7 nm, λ_em_ = 515 nm). Right: Fitting parameters including pre-exponential factors and confidence limits. Figure S66. Left: Raw time-resolved photoluminescence decay of PtL^a^-6 (c ≈ 10^−5^ M) in fluid Ar-purged DCM at r.t., including the residuals (λ_exc_ = 376.7 nm, λ_em_ = 515 nm). Right: Fitting parameters including pre-exponential factors and confidence limits. Figure S67. Left: Raw time-resolved photoluminescence decay of PtL^a^-6 (c ≈ 10^−5^ M) in a frozen glassy matrix of 2Me-THF at 77 K, including the residuals (λ_exc_ = 376.7 nm, λ_em_ = 505 nm). Right: Fitting parameters including pre-exponential factors and confidence limits. Figure S68. Left: Raw time-resolved photoluminescence decay of PtL^a^-12 (c ≈ 10^−5^ M) in fluid air-equilibrated DCM at r.t., including the residuals (λ_exc_ = 376.7 nm, λ_em_ = 515 nm). Right: Fitting parameters including pre-exponential factors and confidence limits. Figure S69. Left: Raw time-resolved photoluminescence decay of PtL^a^-12 (c ≈ 10^−5^ M) in fluid Ar-purged DCM at r.t., including the residuals (λ_exc_ = 376.7 nm, λ_em_ = 515 nm). Right: Fitting parameters including pre-exponential factors and confidence limits. Figure S70. Left: Raw time-resolved photoluminescence decay of PtL^a^-12 (c ≈ 10^−5^ M) in a frozen glassy matrix of 2Me-THF at 77 K, including the residuals (λ_exc_ = 376.7 nm, λ_em_ = 505 nm). Right: Fitting parameters including pre-exponential factors and confidence limits. Figure S71. Left: Raw time-resolved photoluminescence decay of PtL^b^-6 (c ≈ 10^−5^ M) in fluid air-equilibrated DCM at r.t., including the residuals (λ_exc_ = 376.7 nm, λ_em_ = 555 nm). Right: Fitting parameters including pre-exponential factors and confidence limits. Figure S72. Left: Raw time-resolved photoluminescence decay of PtL^b^-6 (c ≈ 10^−5^ M) in fluid Ar-purged DCM at r.t., including the residuals (λ_exc_ = 376.7 nm, λ_em_ = 555 nm). Right: Fitting parameters including pre-exponential factors and confidence limits. Figure S73. Left: Raw time-resolved photoluminescence decay of PtL^b^-6 (c ≈ 10^−5^ M) in a frozen glassy matrix of 2Me-THF at 77 K, including the residuals (λ_exc_ = 376.7 nm, λ_em_ = 540 nm). Right: Fitting parameters including pre-exponential factors and confidence limits. Figure S74. Left: Raw time-resolved photoluminescence decay of PtL^b^-12 (c ≈ 10^−5^ M) in fluid air-equilibrated DCM at r.t., including the residuals (λ_exc_ = 376.7 nm, λ_em_ = 555 nm). Right: Fitting parameters including pre-exponential factors and confidence limits. Figure S75. Left: Raw time-resolved photoluminescence decay of PtL^b^-12 (c ≈ 10^−5^ M) in fluid Ar-purged DCM at r.t., including the residuals (λ_exc_ = 376.7 nm, λ_em_ = 555 nm). Right: Fitting parameters including pre-exponential factors and confidence limits. Figure S76. Left: Raw time-resolved photoluminescence decay of PtL^b^-12 (c ≈ 10^−5^ M) in a frozen glassy matrix of 2Me-THF at 77 K, including the residuals (λ_exc_ = 376.7 nm, λ_em_ = 540 nm). Right: Fitting parameters including pre-exponential factors and confidence limits. Figure S77. Calculated emission spectra at 77 K in THF. Figure S78. DSC profile of PtL^a^-6 on heating with a heating/cooling rate of 10 °C/min. First cycle (black and red curves), second cycle (blue and green curves), third cycle (purple and brown curves). Figure S79. DSC profile of PtL^a^-12 on heating with a heating/cooling rate of 10 °C/min. First cycle (black and red curves), second cycle (blue and green curves), third cycle (purple and brown curves). Figure S80. DSC profile of PtL^b^-6 on heating with a heating/cooling rate of 10 °C/min. First cycle (black and red curves), second cycle (blue and green curves), third cycle (purple and brown curves). Figure S81. DSC profile of PtL^b^-12 on heating with a heating/cooling rate of 10 °C/min. First cycle (black and red curves), second cycle (blue and green curves), third cycle (purple and brown curves). Figure S82. POM images of PtL^a^-12 in the crystalline phase (left) and in the isotropic phase (right) after melting on cooling. Figure S83. POM images of PtL^b^-6 in the glassy phase (left) and in the isotropic phase (right) after melting on cooling. Figure S84. POM images of PtL^b^-12 in the glassy phase (left) and in the isotropic phase (right) after melting on cooling. Figure S85. Left: Emission spectra of solid PtL^a^-12 upon heating (λ_exc_ = 470 nm). Centre: Emission intensity at 512 nm and 661 nm as a function of the temperature. Right: Photographs of solid PtL^a^-12 at different temperatures under UV excitation, λ_exc_ = 365 nm. Figure S86. Left: Emission spectra of solid PtL^b^-6 upon heating (λ_exc_ = 470 nm). Centre: Emission intensity at 512 nm and 661 nm as a function of the temperature. Right: Photographs of solid PtL^a^-12 at different temperatures under UV excitation, λ_exc_ = 365 nm. Figure S87. Left: Emission spectra of solid PtL^b^-12 upon heating (λ_exc_ = 470 nm). Centre: Emission intensity at 512 nm and 661 nm as a function of the temperature. Right: Photographs of solid PtL^a^-12 at different temperatures under UV excitation, λ_exc_ = 365 nm.
