# Supplementary material for: Pt(II) Complexes with Tetradentate C^N*N^C Luminophores: From Supramolecular Interactions to Temperature-Sensing Materials with Memory and Optical Readouts
Source: Molecules. 2023 Oct 30;28(21):7353. doi: 10.3390/molecules28217353 (PMC10649584; doi:10.3390/molecules28217353)
Supplement: Supplementary file 1 [file molecules-28-07353-s001.zip › molecules-2638431-supplementary.pdf]

Supporting Information

Pt(II) complexes with tetradentate C^N*N^C luminophores: from supramolecular interactions to temperature-sensing materials with memory and optical redouts

Matias E. Gutierrez Suburu [1,2], Meik Blanke [3], Alexander Hepp [1], Oliver Maus [1], Dominik Schwab [4], Nikos L. Doltsinis [4], Wolfgang G. Zeier [1], Michael Giese [3], Jens Voskuhl [3] and Cristian A. Strassert [1,2,*]

[1]Institut für Anorganische und Analytische Chemie, Universität Münster, Corrensstraße 28/30, D-48149 Münster, Germany

[2]Center of Nanotechnology (CeNTech), Center for Soft Nanosciences (SoN), Cells in Motion Interfaculty Cluster (CiMIC), Universität Münster, Heisenbergstraße 11, D-48149 Munster, Germany

[3]Center for Nanointegration Duisburg-Essen (CENIDE), Faculty of Chemistry (Organic Chemistry), University of Duisburg-Essen, Universitätsstraße 7, D-45141 Essen, Germany

[4]Center for Multiscale Theory and Computation, Institut für Festkörpertheorie, Universität Münster, Wilhelm-Klemm-Straße 10, D-48149 Münster, Germany

*Correspondence: ca.s@wwu.de

1) Synthesis and characterization……………………………………………2
2) Photophysical characterization………………………………………41
3) Theoretical calculations…………………………………………………….50
4) Thermal studies……………………………………………………………..…51
5) References………………………………………………………………………..…55

1) Synthesis and characterization

All the synthetic precursors, reagents and solvents (analytical grade) were used as purchased without further purification.

NMR spectra were obtained at the Institut für Anorganische und Analytische Chemie (WWU), using an Avance Neo 500 MHz or a Avance I or III 400 MHz. All measurements were performed at room temperature unless mentioned otherwise. The ^{1}H-NMR and ^{13}C-NMR chemical shifts (δ) of the signals are given in parts per million and are referenced to the residual proton signal in the deuterated solvent DCM-d_2 (^{1}H: 5.32 ppm / ^{13}C: 54.0 ppm). The signal multiplicities are abbreviated as follows: s, singlet; d, doublet; t, triplet; q, quartet; m, multiplet.

Exact mass (EM) determination by mass spectrometry (MS) was carried out at the Organisch-Chemisches Institut in Münster (WWU) using a LTQ Orbitap LTQ XL (Thermo-Fisher Scientific, Bremen) with electron spray ionization (ESI).

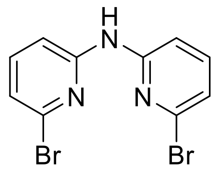

Bis(6-bromopyridin-2-yl)amine (1)

6-bromopyridin-2-amine (1.439 g, 8.3 mmol) was dissolved in dry DMF (100 mL). The solution was cooled in an ice bath. NaH (0.626 g, 26.1 mmol) was added slowly while stirring. H_2 evolved as the mixture turned brown. 2,6 dibromopyridine (1.979 g, 8.4 mmol) was added and the mixture was stirred overnight at room temperature. The reaction was quenched by the addition of H_2O. The organic phase was separated from the liquid phase and dried under vacuum resulting in a red solid. This solid was purified by column chromatography using cyclohexane/ethyl acetate (10:1) as eluent to yield a white crystalline solid (1.566 g, 57%).

^{1}H-NMR (500 MHz, CD_2Cl_2): δ = 7.53 – 7.45 (m, 5H), 7.07 (dd, J = 5.8, 2.5 Hz, 2H).

^{13}C-NMR (126 MHz, CD_2Cl_2): δ = 153.5, 140.5, 139.7, 120.8, 110.4.

EM-MS (ESI+, MeOH, m/z): calcd. for $[M + H]^+$ = 329.90590, found 329.90587.

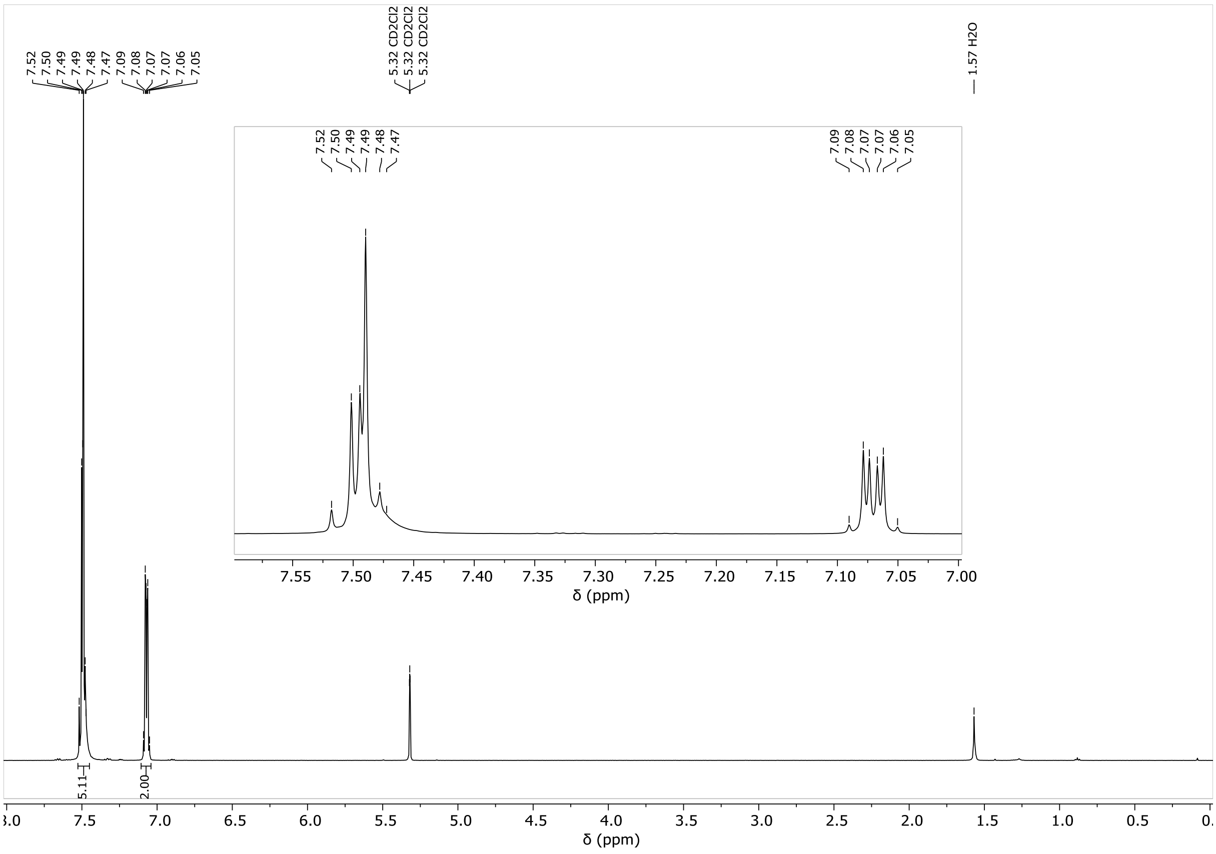

Figure S1. ^{1}H-NMR spectrum (500 MHz, CD_2Cl_2) of **1**.

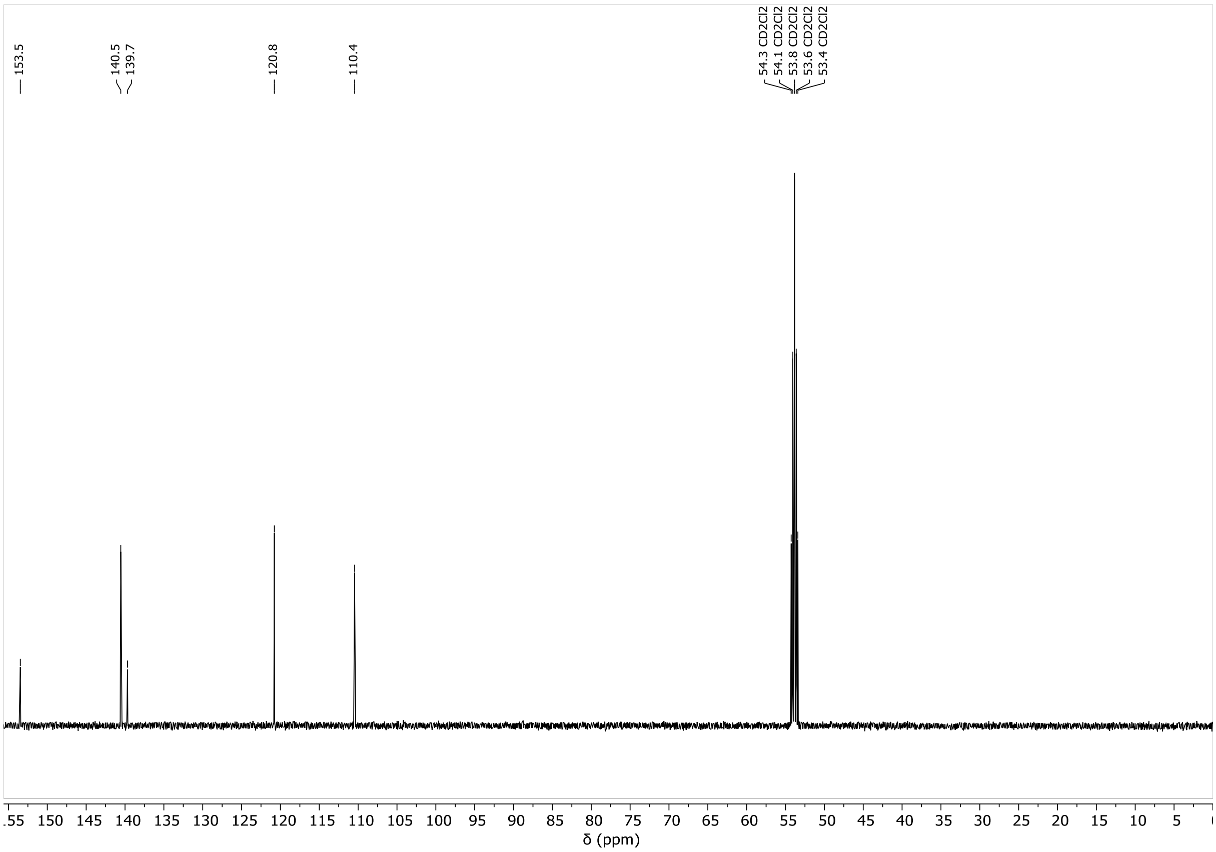

Figure S2. ^{13}C-{^{1}H}-NMR spectrum (126 MHz, CD_2Cl_2) of **1**.

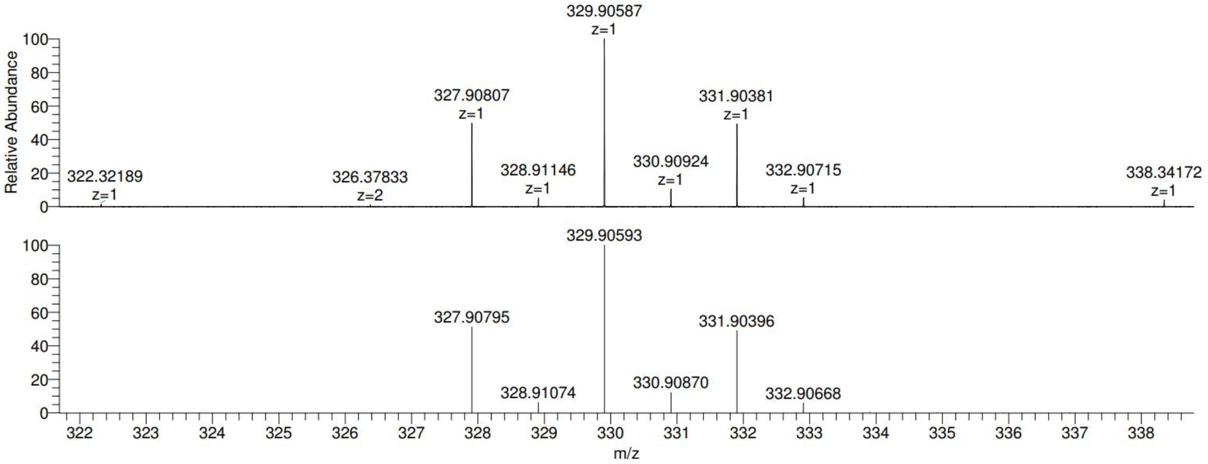

Figure S3. ESI-MS (MeOH) of **1**.

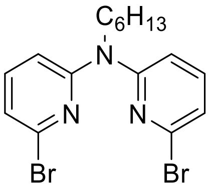

N,N-bis(6-bromopyridin-2-yl)-N-hexylamine (2-6)

Bis(6-bromopyridin-2-yl)amine (1.000 g, 2.51 mmol) was dissolved in dry DMF (30 mL). The solution was cooled in an ice bath. NaH (0.181 g, 7.51 mmol) was added slowly while stirring. H_2 evolved as the mixture turned yellowish. 1-bromohexane (0.5 mL, 3.56 mmol) was added and the mixture was stirred overnight at room temperature. The reaction was quenched by the addition of H_2O (50 mL). The mixture was extracted with CH_2Cl_2 (25 mL x 3). The combined organic layers were washed with brine (50 mL) and dried with Na_2SO_4. The crude product was purified by column chromatography (SiO_2) using cyclohexane/CH_2Cl_2 (10:1) as eluent to yield a colourless oil (0.800 g, 77%).

^{1}H NMR (500 MHz, CD_2Cl_2): δ = 7.44 – 7.38 (m, 2H), 7.09 (d, J = 8.1 Hz, 2H), 7.06 (d, J = 7.6 Hz, 2H), 4.12 – 4.06 (m, 2H), 1.67 (p, J = 7.4 Hz, 2H), 1.32 (h, J = 7.4, 6.9 Hz, 6H), 0.88 (t, J = 7.0 Hz, 3H).

^{13}C NMR (126 MHz, CD_2Cl_2): δ = 157.0, 140.0, 140.0, 139.8, 121.2, 113.3, 48.8, 31.8, 28.1, 26.9, 23.0, 23.0, 14.2.

EM-MS (ESI+, MeOH, m/z): calcd. for $[M + H]^+$ = 413.99980, found 413.99965; calcd. for $[M + Na]^+$ = 435.98175, found 435.98157; calcd. for $[M + K]^+$ = 451.95568, found 451.95564.

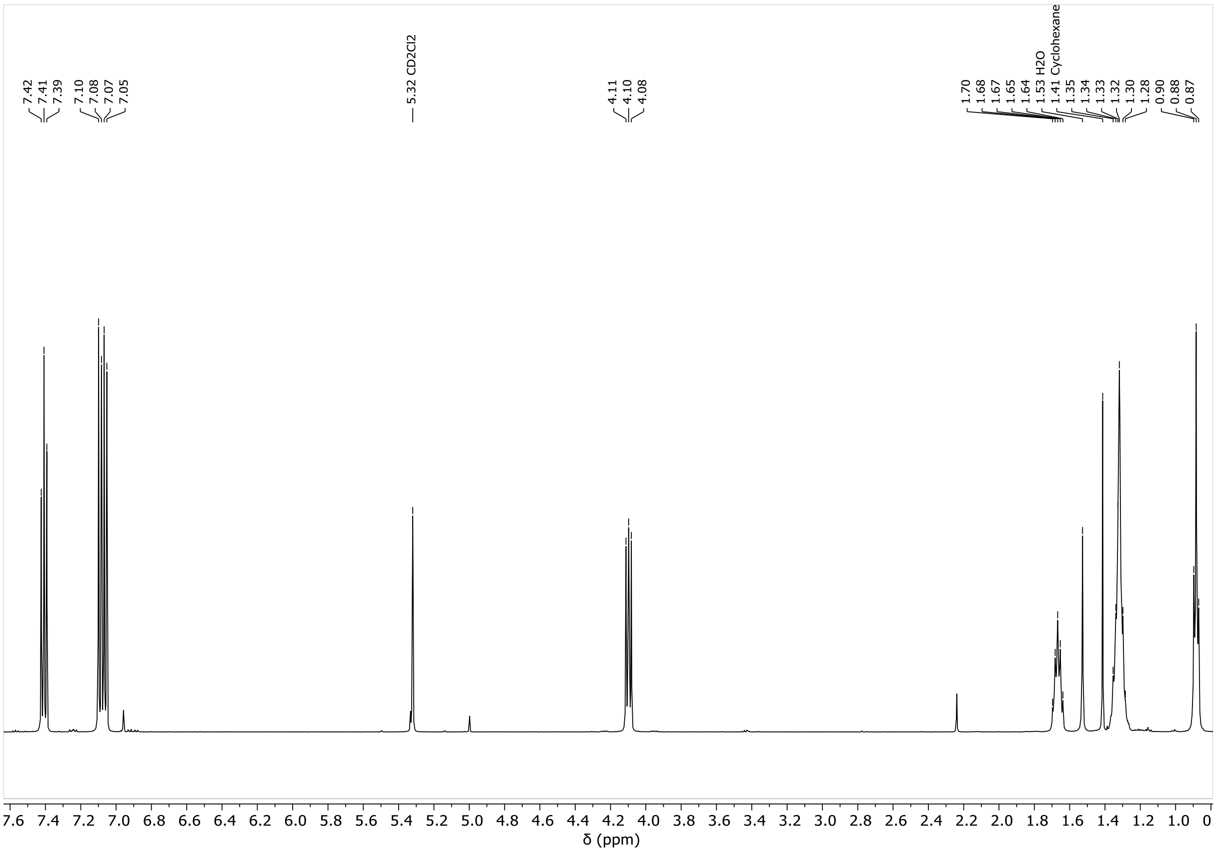

Figure S4. ^{1}H-NMR spectrum (500 MHz, CD_2Cl_2) of **2-6**.

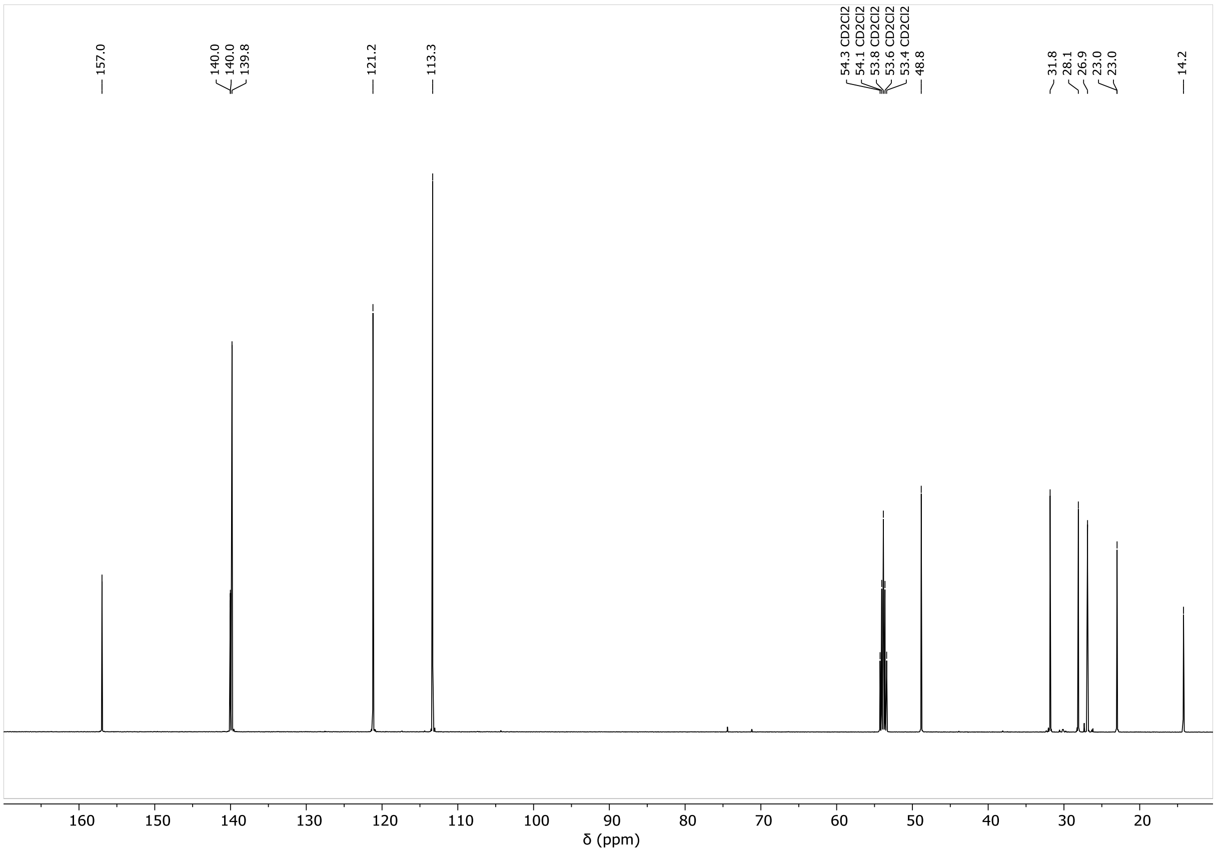

Figure S5. ^{13}C-{^{1}H}-NMR spectrum (126 MHz, CD_2Cl_2) of **2-6**.

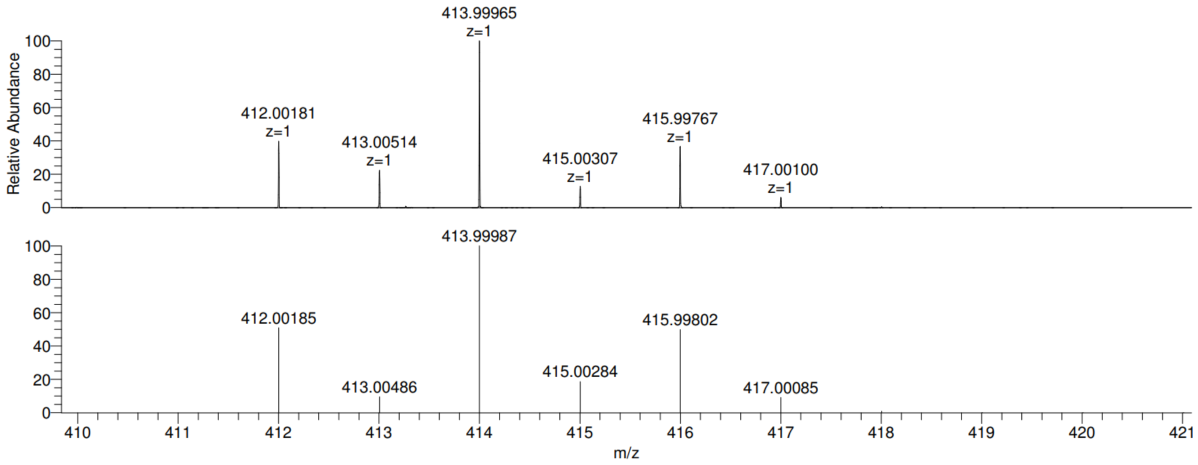

Figure S6. ESI-MS (MeOH) of **2-6**.

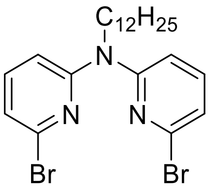

N,N-bis(6-bromopyridin-2-yl)-N-dodecylamine (2-12)

Bis(6-bromopyridin-2-yl)amine (1.016 g, 3.08 mmol) was dissolved in dry DMF (30 mL). The solution was cooled in an ice bath. NaH (0.265 g, 11.04 mmol) was added slowly while stirring. H_2 evolved as the mixture turned yellowish. 1-bromododecane (1.0 mL, 4.16 mmol) was added and the mixture was stirred overnight at room temperature. The reaction was quenched by the addition of H_2O (50 mL). The mixture was extracted with CH_2Cl_2 (25 mL x 3). The combined organic layers were washed with brine (50 mL) and dried with Na_2SO_4. The crude product was purified by column chromatography (SiO_2) using cyclohexane/CH_2Cl_2 (10:1) as eluent to yield an oil (2.155 g, 89%).

^{1}H NMR (500 MHz, CD_2Cl_2): δ = 7.41 (t, J = 7.9 Hz, 2H), 7.09 (d, J = 8.2 Hz, 2H), 7.06 (d, J = 7.5 Hz, 2H), 4.13 – 4.07 (t, J = 7.6 Hz, 2H), 1.67 (p, J = 7.7 Hz, 2H), 1.36 – 1.24 (m, 18H), 0.88 (t, J = 6.9 Hz, 3H).

^{13}C NMR (126 MHz, CD_2Cl_2): δ = 157.0, 140.0, 139.8, 121.2, 113.3, 48.9, 32.3, 30.1, 30.1, 30.0, 30.0, 29.7, 29.7, 28.1, 27.2, 23.1, 14.3.

EM-MS (ESI+, MeOH, m/z): calcd. for $[M + H]^+$ = 496.09594, found 496.09575.

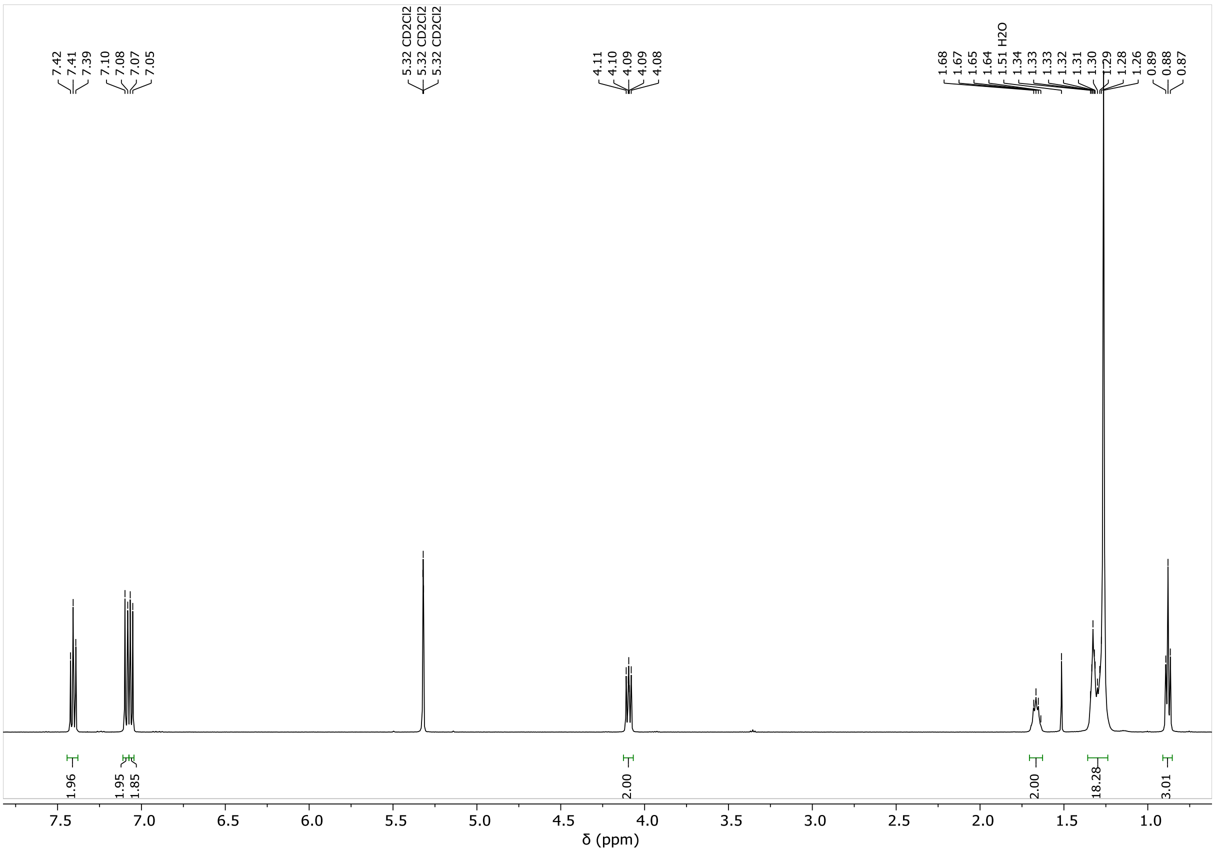

Figure S7. ^{1}H-NMR spectrum (500 MHz, CD_2Cl_2) of **2-12**.

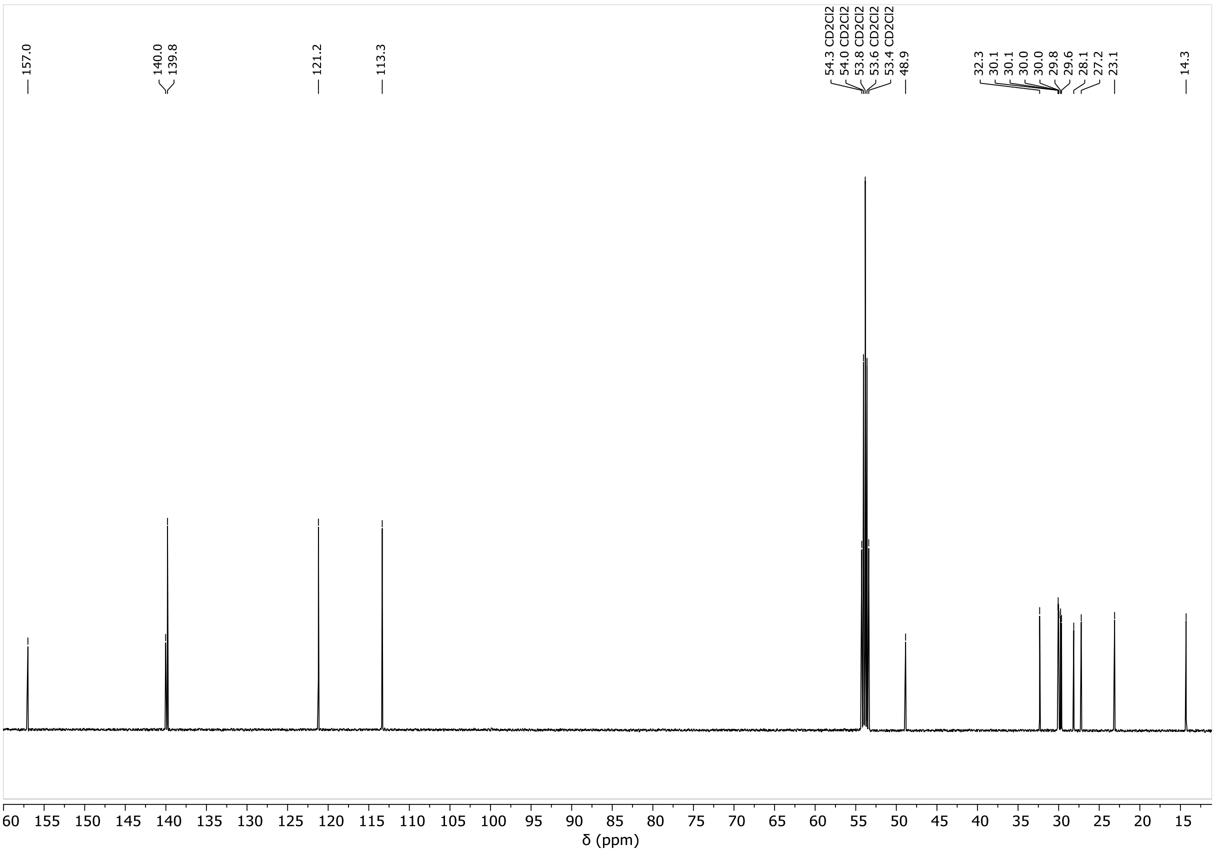

Figure S8. ^{13}C-{^{1}H}-NMR spectrum (126 MHz, CD_2Cl_2) of **2-12**.

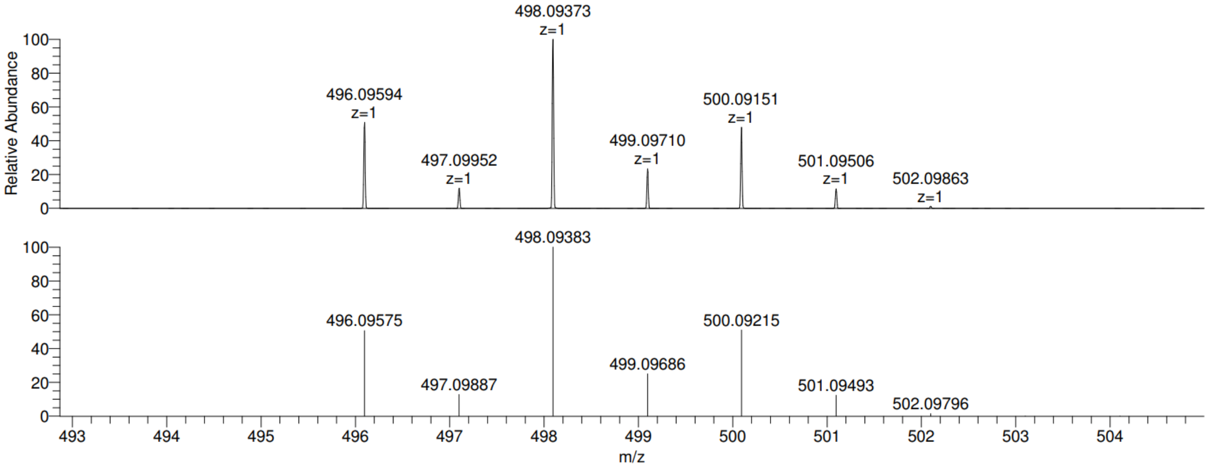

Figure S9. ESI-MS (MeOH) of **2-12**.

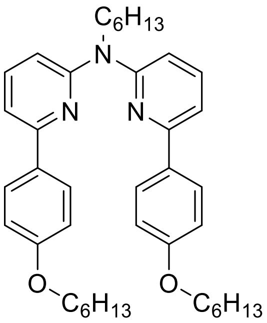

N-hexyl-N,N-bis(6-(4-(hexyloxy)phenyl)pyridine-2-yl)-amine (L^a-6)

2-6 (1.124 g, 2.72 mmol), $Pd(PPh_3)_4$ (0.376 g, 0.33 mmol,) and 4-hydroxyphenylboronic acid (0.963 g, 6.98 mmol) were placed in a 250 mL two neck flask and dissolved in 100 mL of THF. The solution was purged by bubbling with Ar and heated to 75 °C. Aqueous K_2CO_3 (2 M, 10 mL, 20 mmol) was added with a syringe. The resulting yellow solution was stirred at 75 °C overnight. THF was removed under reduced pressure. The residue was transferred to a separation funnel with diluted HCl (25 mL) and extracted with CH_2Cl_2 (25 mL x 3). The combined organic layers were washed with brine (25 mL) and dried with Na_2SO_4. The solution was concentrated and then filtered through a SiO_2 plug, washed with CH_2Cl_2/MeOH (100:1) while collecting the yellow solution. CH_2Cl_2 was evaporated and the crude product combined with K_2CO_3 (1.009 g, 7.30 mmol) in 75 mL of butanone. The mixture was purged by bubbling with Ar and heated up to reflux. Then $C_{12}H_{25}Br$ (0.8 mL, 5.70 mmol) was added and the mixture was reacted for 14 h. The mixture was cooled to room temperature and the solvent was removed under reduced pressure. The product was dissolved in CH_2Cl_2 (50 mL), washed with water (25 mL) and brine (25 mL), dried with Na_2SO_4 and the solvent was evaporated. The product was purified by column chromatography (SiO_2) using cyclohexane/ethyl acetate (100:1 to 100:3) to yield a white solid (0.627 g, 38%).

^{1}H NMR (500 MHz, CD_2Cl_2): δ = 8.01 (d, J = 8.9 Hz, 4H), 7.58 (t, J = 7.9 Hz, 2H), 7.29 (d, J = 7.5 Hz, 2H), 7.09 (d, J = 8.3 Hz, 2H), 6.98 (d, J = 8.9 Hz, 4H), 4.38 (t, J = 7.6 Hz, 2H), 4.03 (t, J = 6.6 Hz, 4H), 1.83 (m, 6H), 1.58 – 1.39 (m, 6H), 1.42 – 1.26 (m, 12H), 0.94 (t, J = 7.0, 3H), 0.90 (t, J = 7.2 Hz, 3H).

^{13}C NMR (126 MHz, CD_2Cl_2): δ = 160.6, 157.6, 155.7, 138.2, 132.4, 128.4, 115.0, 112.8, 112.4, 68.7, 48.8, 32.4, 32.2, 29.9, 28.9, 27.5, 26.3, 23.3, 23.2, 14.5, 14.4.

EM-MS (ESI+, MeOH, m/z): calcd. for $[M + H]^+$ = 608.42105, found 608.42080

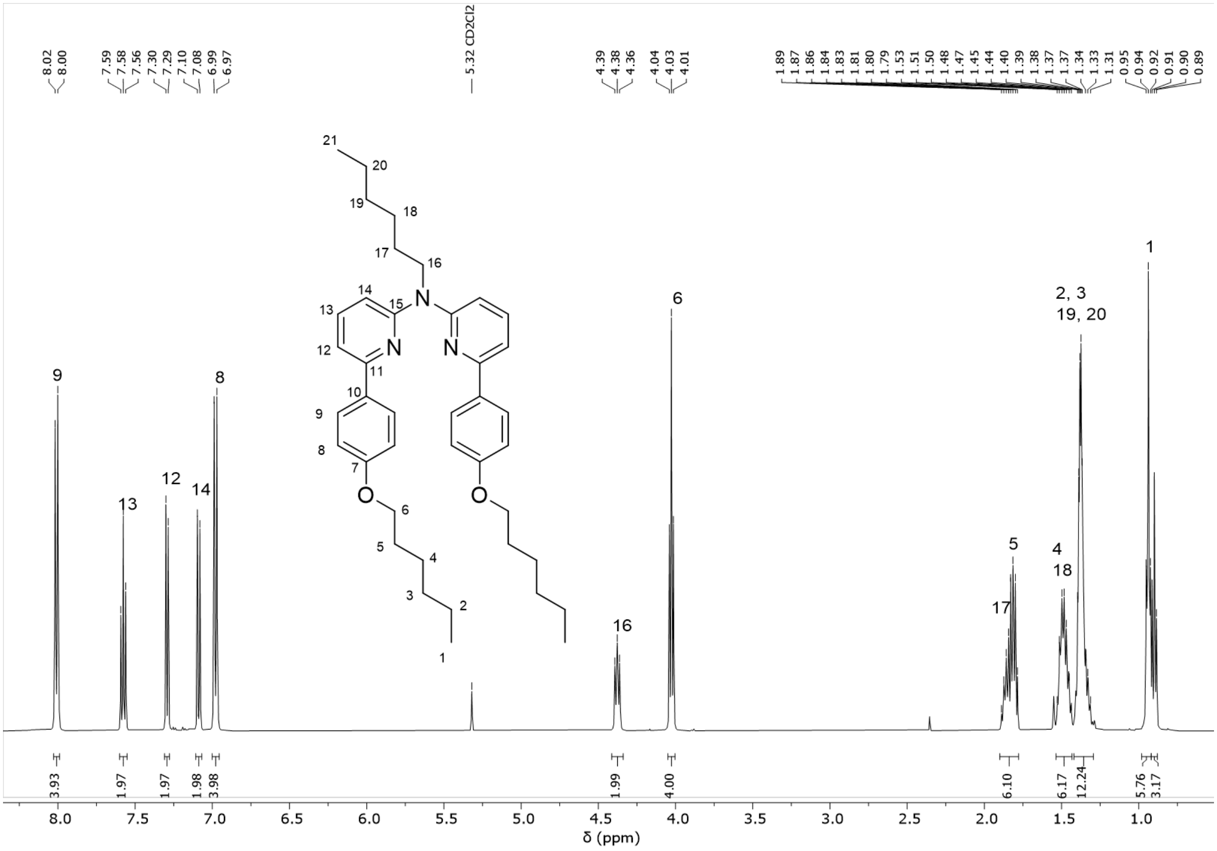

Figure S10. ^{1}H-NMR spectrum (500 MHz, CD_2Cl_2) of **L^{a}-6**.

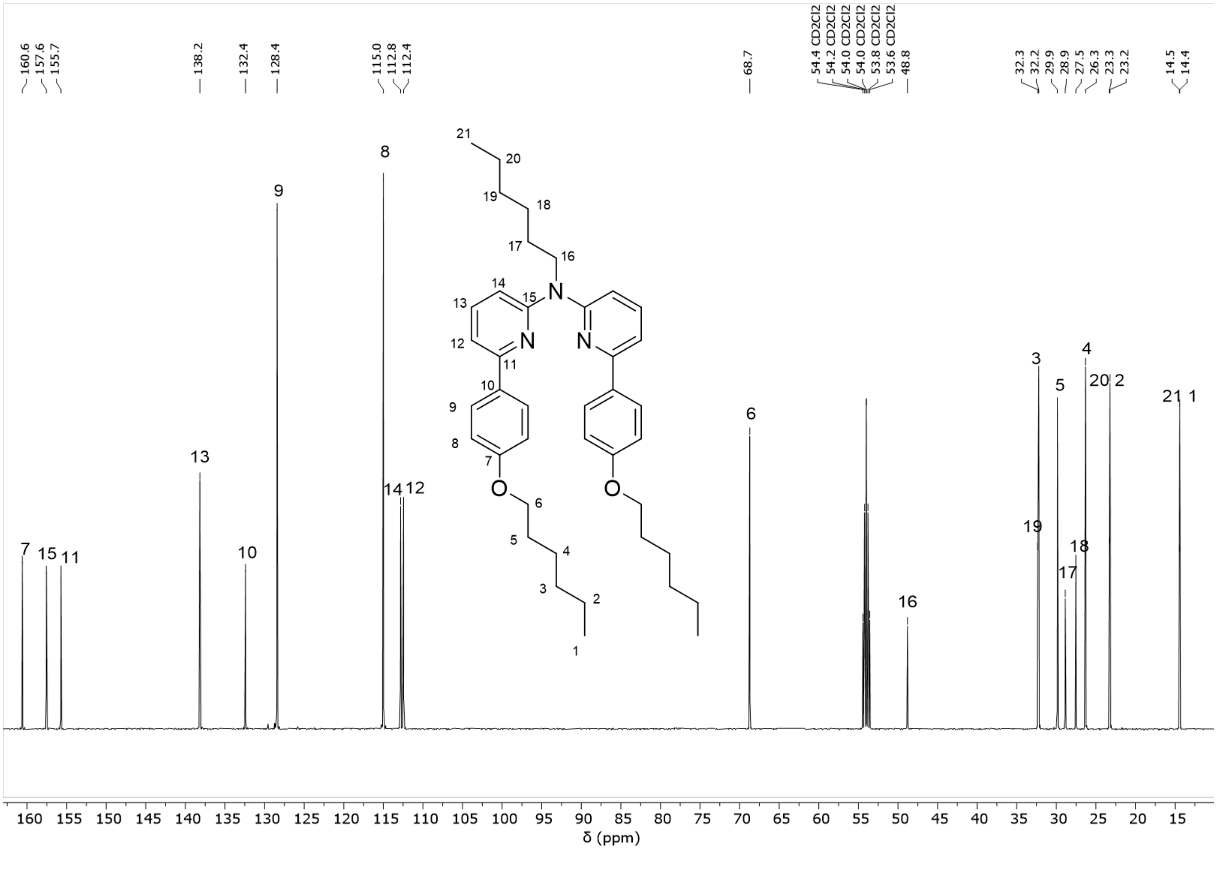

Figure S11. ^{13}C-{^{1}H}-NMR spectrum (126 MHz, CD_2Cl_2) of **L^{a}-6**.

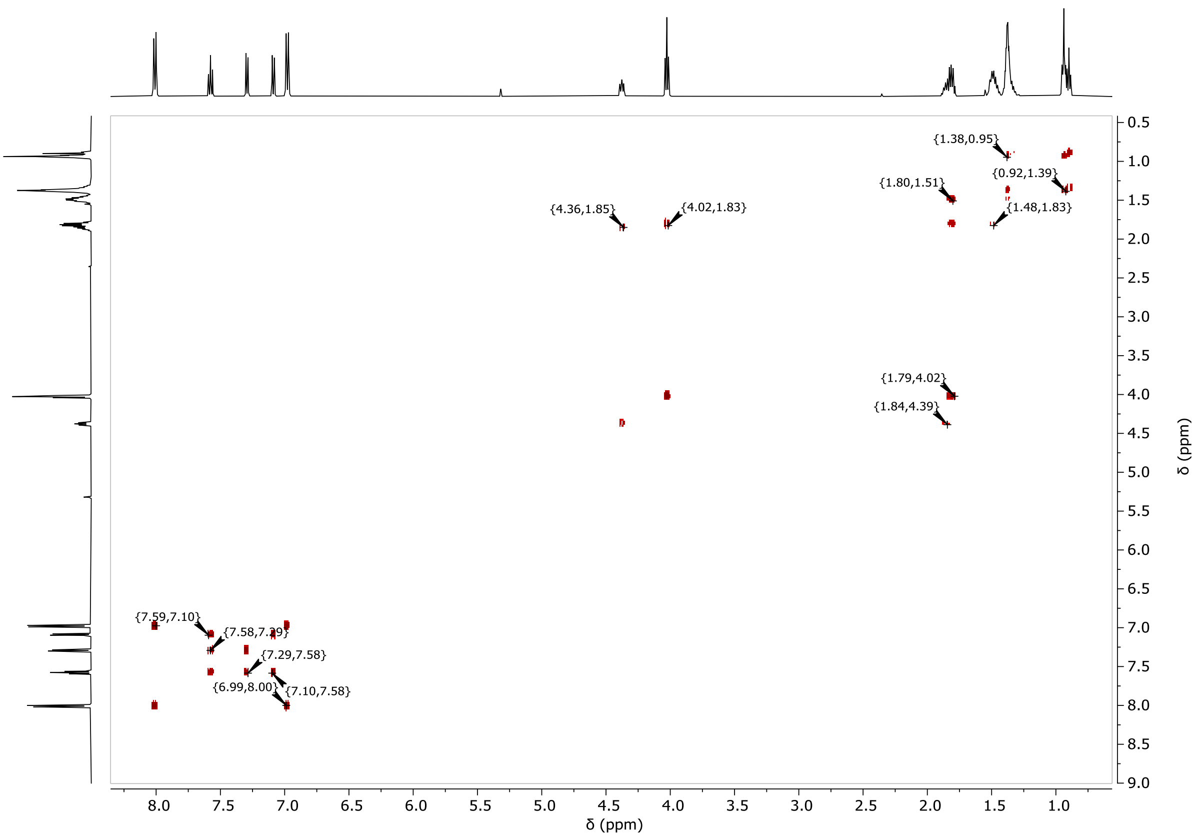

Figure S12. $^1H/^1H$-COSY-NMR spectrum (500 MHz/500 MHz, CD_2Cl_2) of **L^a-6.**

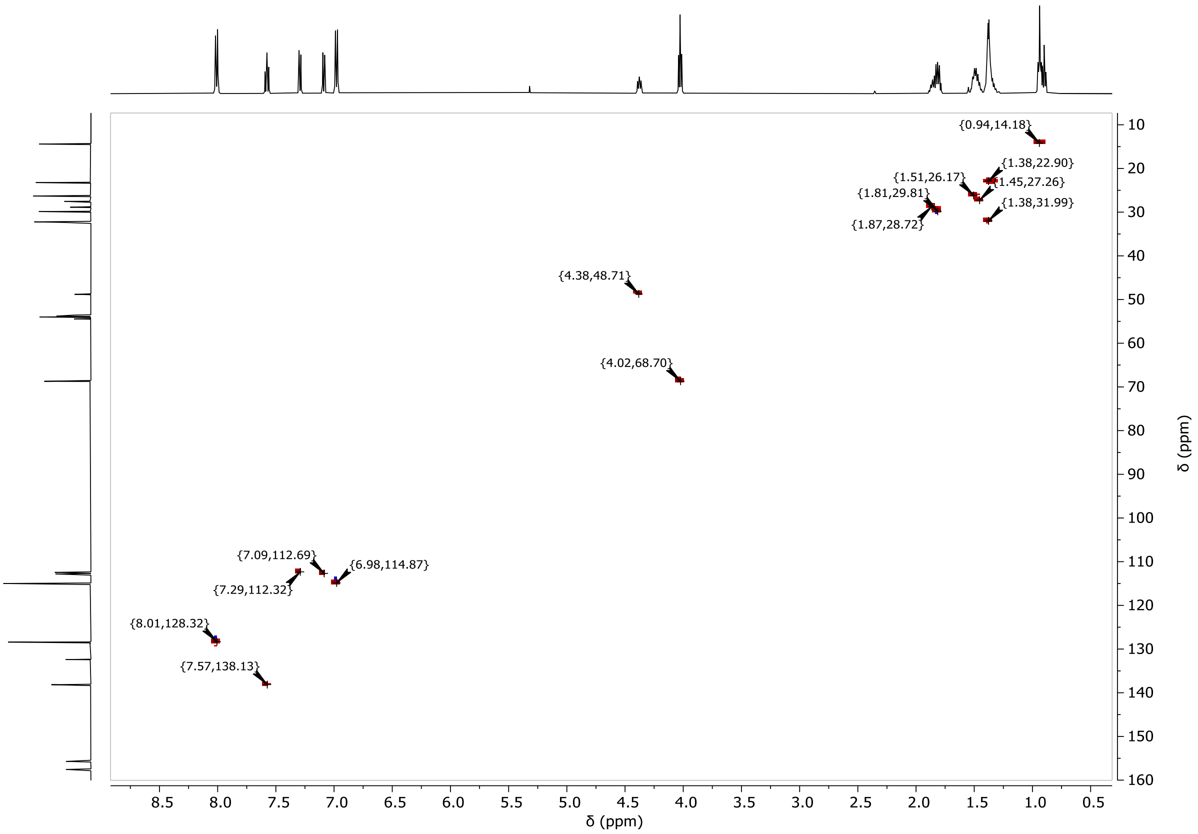

Figure S13. $^1H/^{13}C$-gHSQC-NMR spectrum (500 MHz/126 MHZ, CD_2Cl_2) of **L^a-6**.

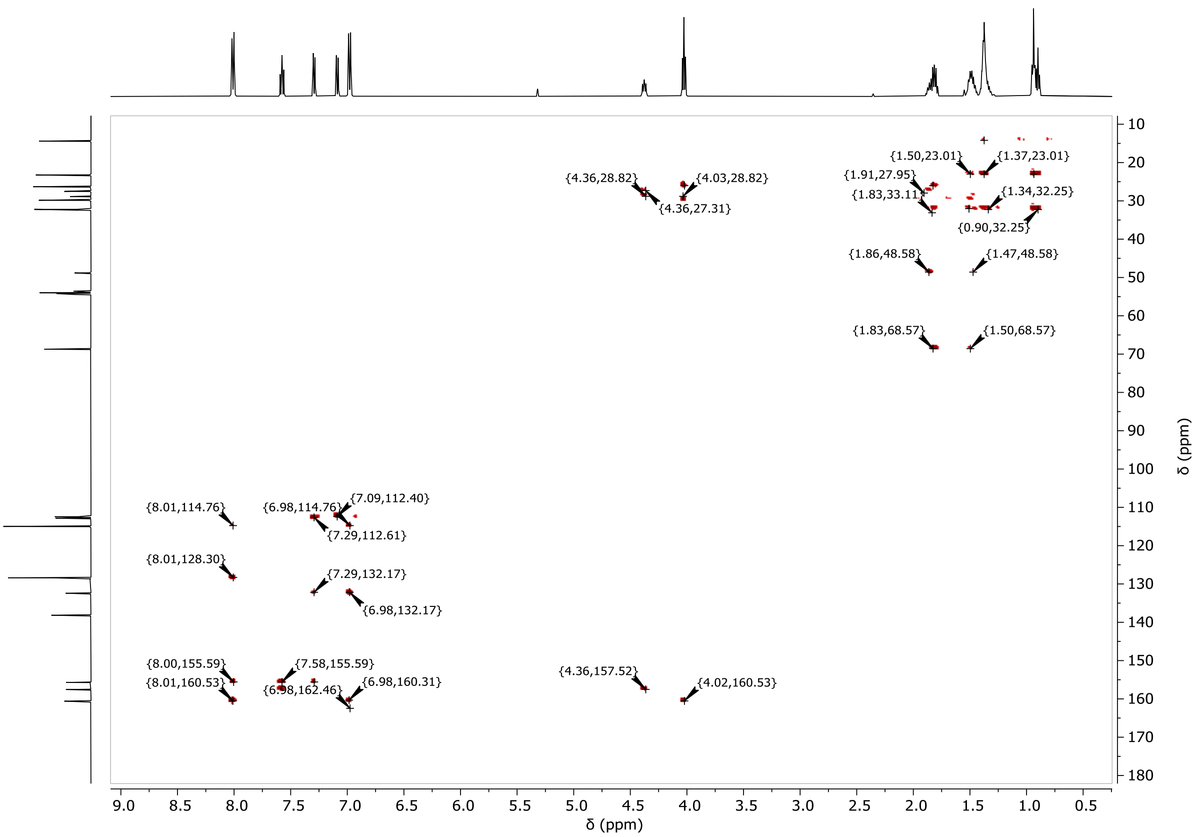

Figure S14. $^{1}H/^{13}C$-gHMBC-NMR spectrum (500 MHz/126 MHz, CD_2Cl_2) of **L^a-6**.

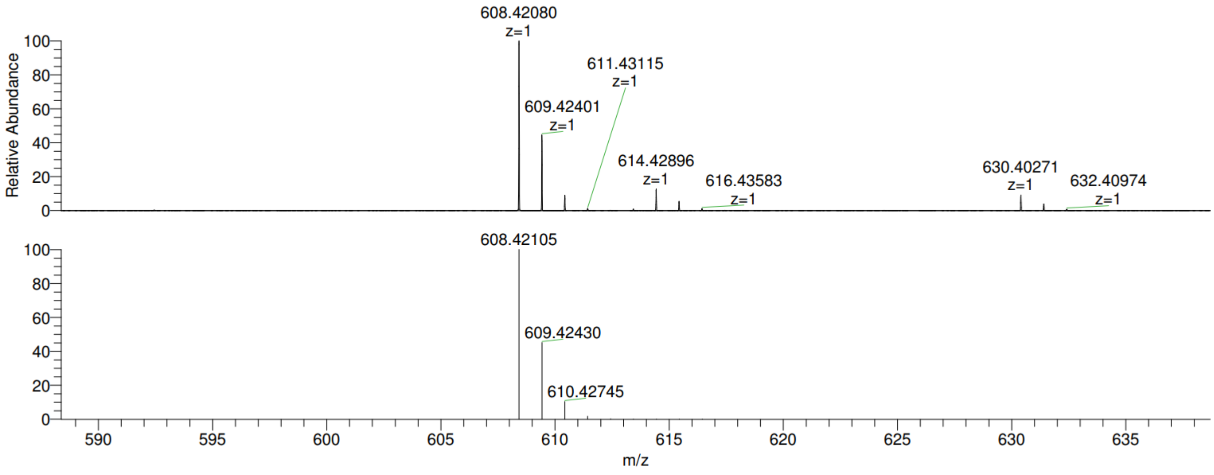

Figure S15. ESI-MS (MeOH) of **L^a-6**.

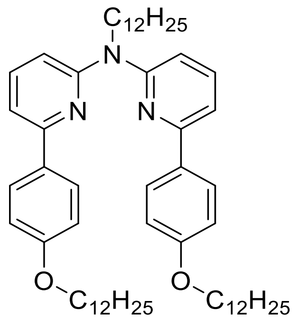

N-dodecyl-N,N-bis(6-(4-(dodecyloxy)phenyl)pyridine-2-yl)-amine (L^a-12)

2-12 (1.0981 g, 2.21 mmol), $Pd(PPh_3)_4$ (0.306 g, 0.26 mmol,) and 4-hydroxyphenylboronic acid (0.786 g, 5.70 mmol) were placed in a 250 mL two-necked flask and dissolved in 100 mL of THF. The solution was purged by bubbling with Ar and heated to 75 °C. Then, aqueous K_2CO_3 (2 M, 10 mL, 20 mmol) was added with a syringe. The resulting yellow solution was stirred at 75 °C overnight. THF was removed under reduced pressure. The residue was transferred to a separation funnel with diluted HCl (25 mL) and extracted with CH_2Cl_2 (25 mL x 3). The combined organic layers were washed with brine (25 mL) and dried with Na_2SO_4. The solution was concentrated and then filtered through a SiO_2 plug, washed with CH_2Cl_2/MeOH (100:1) while collecting the yellow solution. CH_2Cl_2 was evaporated and the crude product combined with K_2CO_3 (0.916 g, 6.63 mmol) in 75 mL of butanone. The mixture was purged by bubbling with Ar and heated up to reflux. Then, $C_{12}H_{25}Br$ (1.3 mL, 5.55 mmol) was added and the mixture reacted for 14 h. The mixture was cooled to room temperature and the solvent was removed under reduced pressure. The product was dissolved in CH_2Cl_2 (50 mL) and washed with water (25 mL) and brine (25 mL), dried with Na_2SO_4 and the solvent was evaporated. The product was purified by column chromatography (SiO_2) using cyclohexane/ethyl acetate (100:1 to 50:1) to yield a white solid (0.700 g, 30%).

^{1}H NMR (500 MHz, CD_2Cl_2): δ = 8.00 (d, *J* = 8.9 Hz, 4H), 7.57 (t, *J* = 7.9 Hz, 2H), 7.29 (d, *J* = 7.5 Hz, 2H), 7.08 (d, *J* = 8.1 Hz, 2H), 6.97 (d, *J* = 8.9 Hz, 4H), 4.36 (t, *J* = 7.2 Hz, 2H), 4.02 (t, *J* = 6.6 Hz, 4H), 1.90 – 1.75 (m, 6H), 1.50 – 1.18 (m, 54H), 0.89 (d, *J* = 4.6 Hz, 9H).

^{13}C NMR (126 MHz, CD_2Cl_2): δ = 160.6, 157.6, 155.7, 138.2, 132.4, 128.4, 115.0, 112.8, 112.4, 68.7, 48.8, 32.5, 32.5, 30.3, 30.3, 30.3, 30.2, 30.2, 30.1, 30.0, 30.0, 30.0, 29.9, 28.9, 27.9, 26.6, 23.3, 14.5.

EM-MS (ESI+, MeOH, m/z): calcd. for $[M + H]^+$ = 860.70276, found 860.70329.

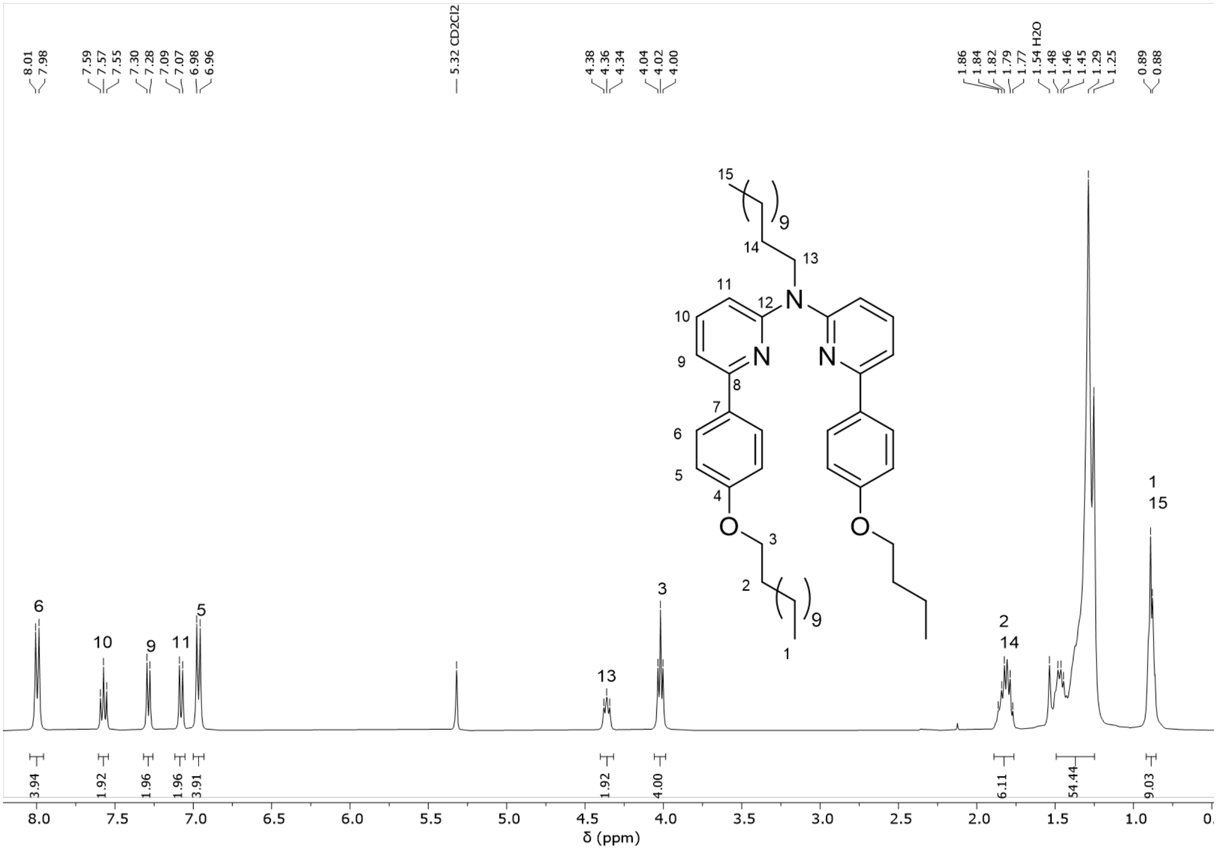

Figure S16. ^{1}H-NMR spectrum (500 MHz, CD_2Cl_2) of **L^a-12**.

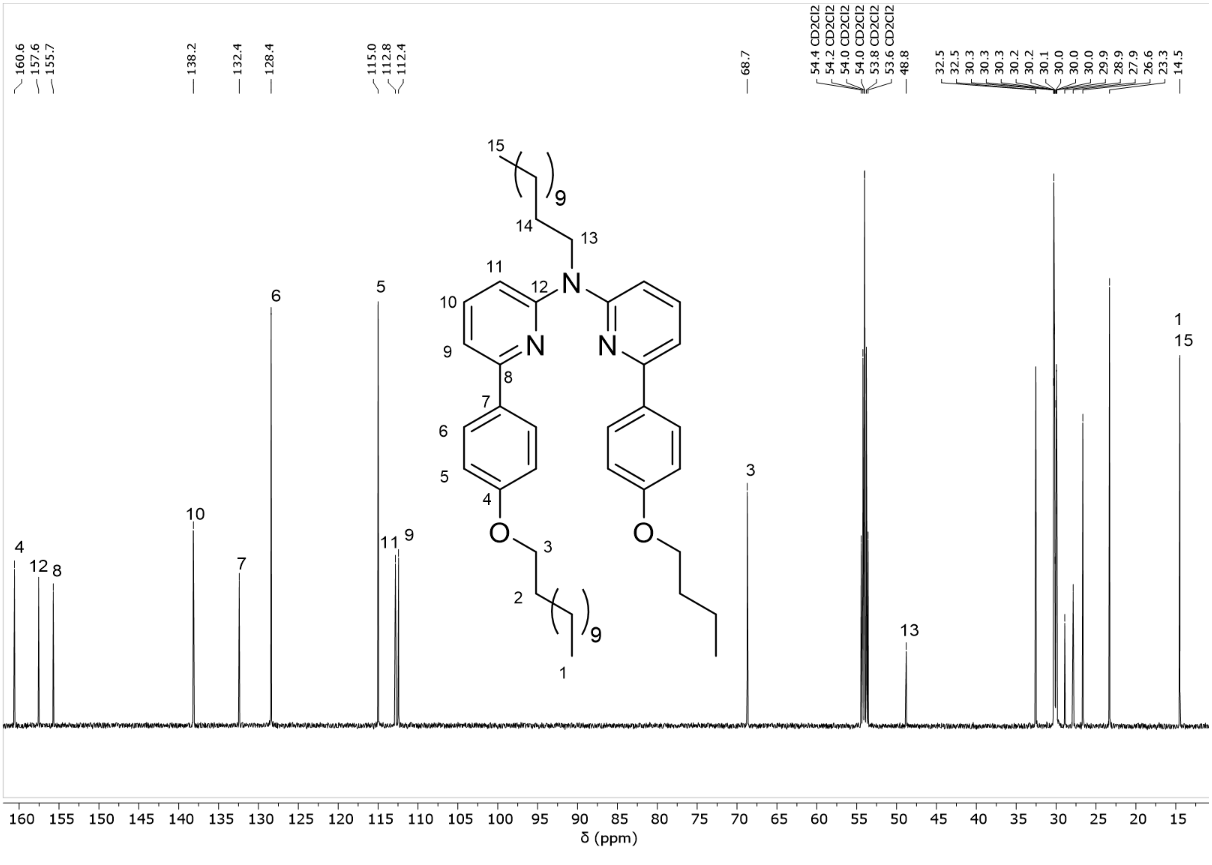

Figure S17. ^{13}C-{^{1}H}-NMR spectrum (126 MHz, CD_2Cl_2) of **L^a-12**.

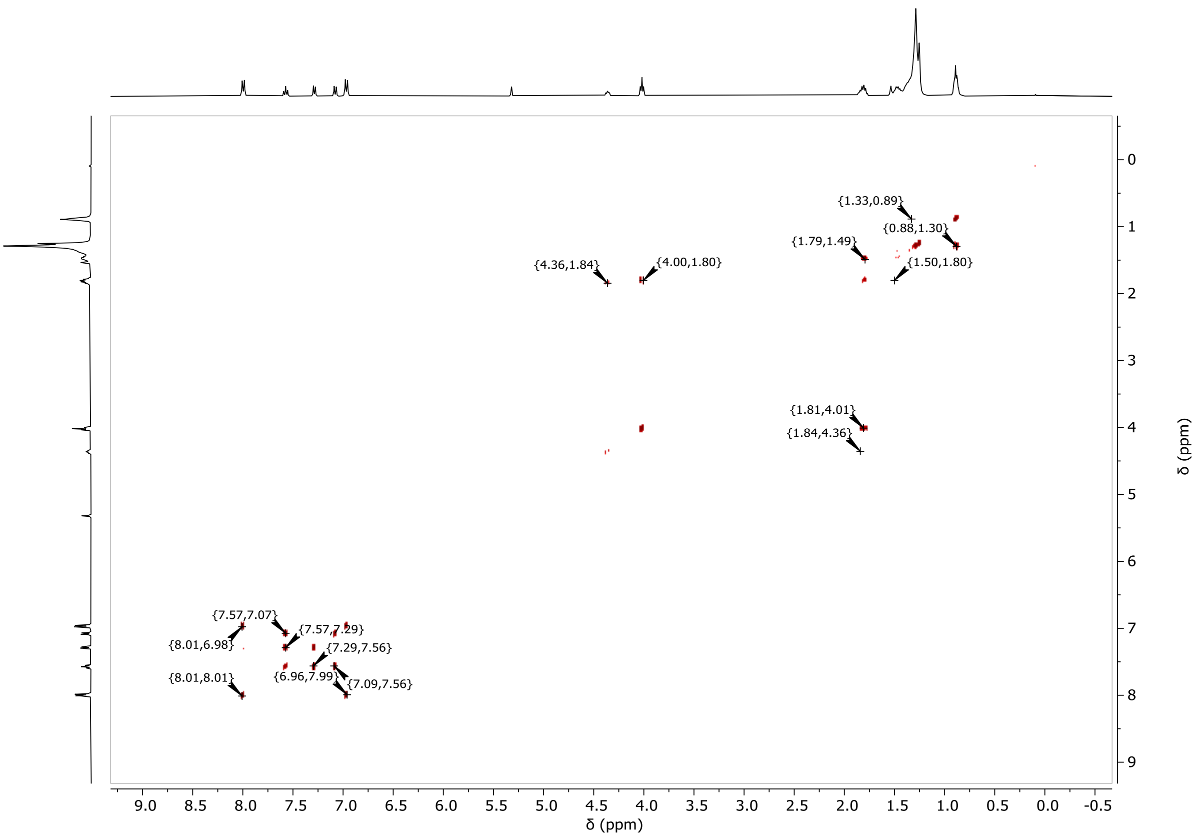

Figure S18. $^{1}H/^{1}H$-COSY-NMR spectrum (500 MHz/500 MHz, CD_2Cl_2) of **L^{a}-12.**

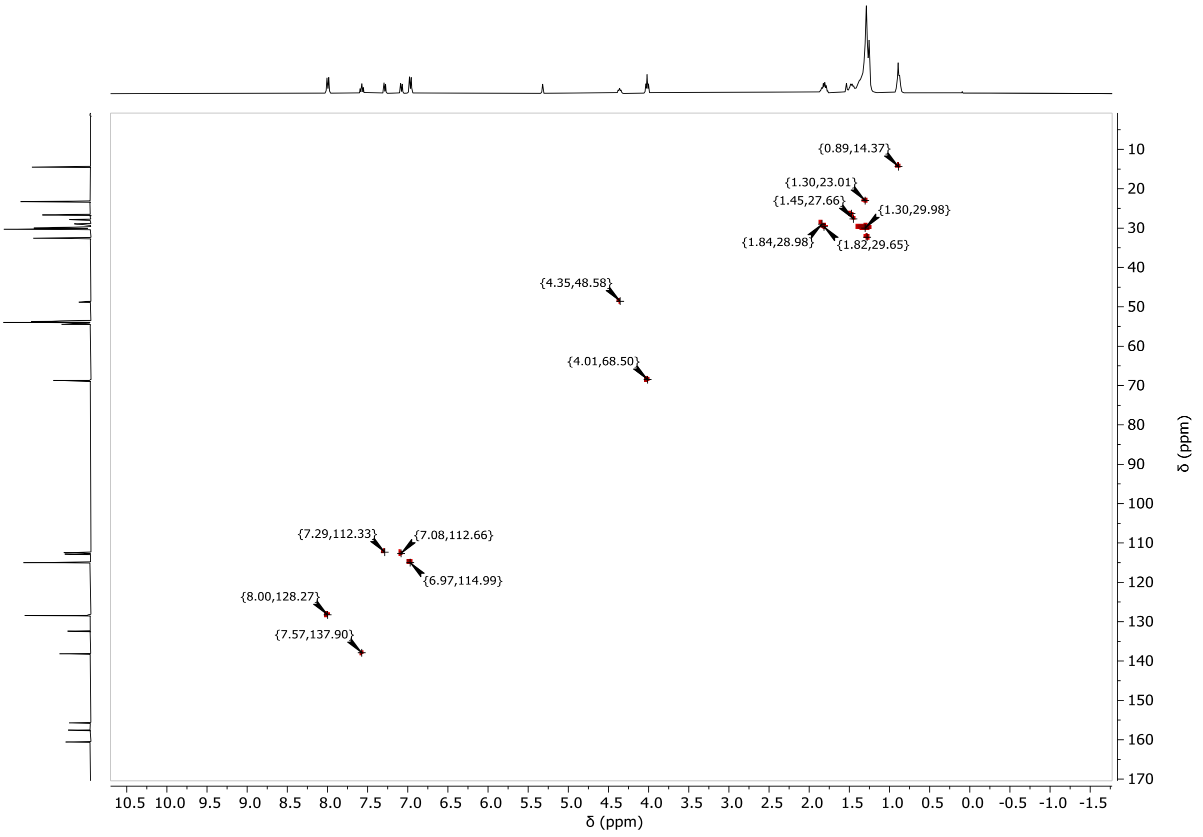

Figure S19. $^{1}H/^{13}C$-gHSQC-NMR spectrum (500 MHz/126 MHZ, CD_2Cl_2) of **L^{a}-12**.

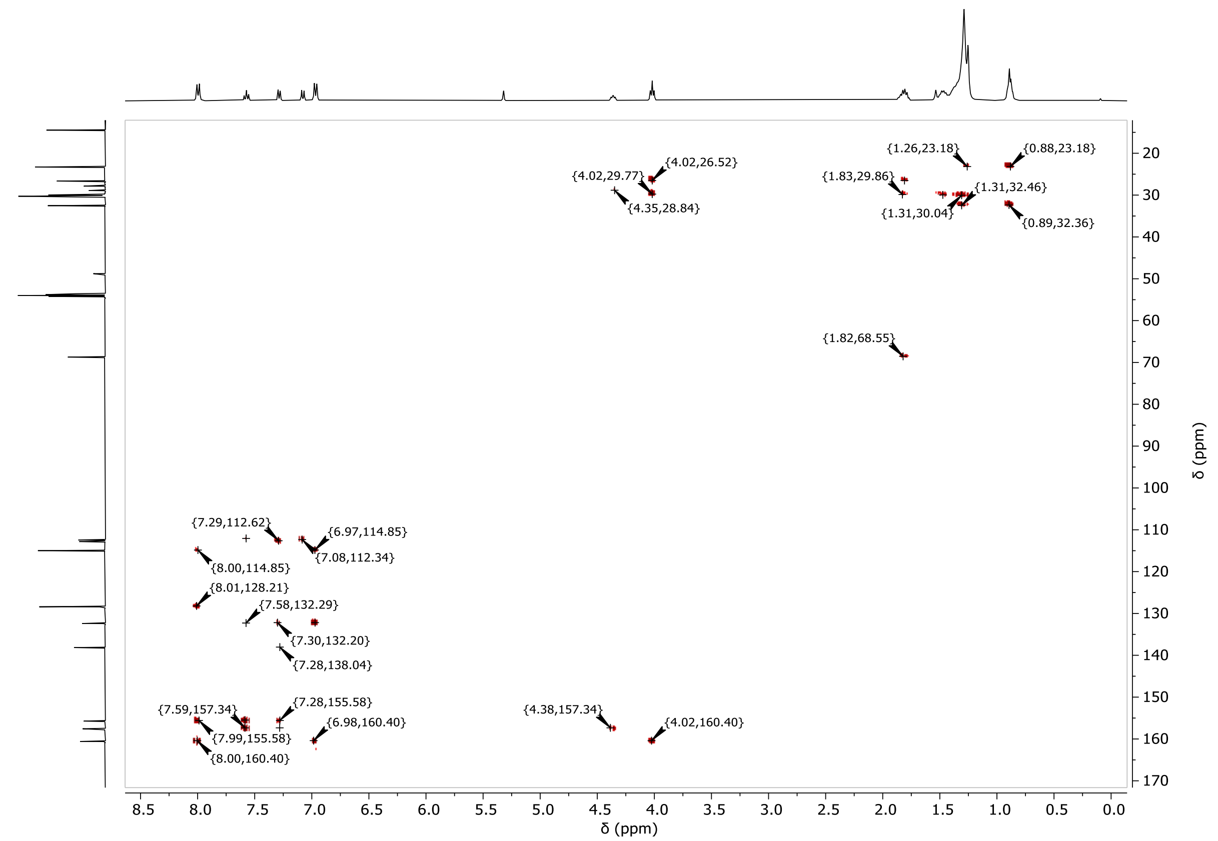

Figure S20. $^{1}H/^{13}C$-gHMBC-NMR spectrum (500 MHz/126 MHz, CD_2Cl_2) of **L^a-12**.

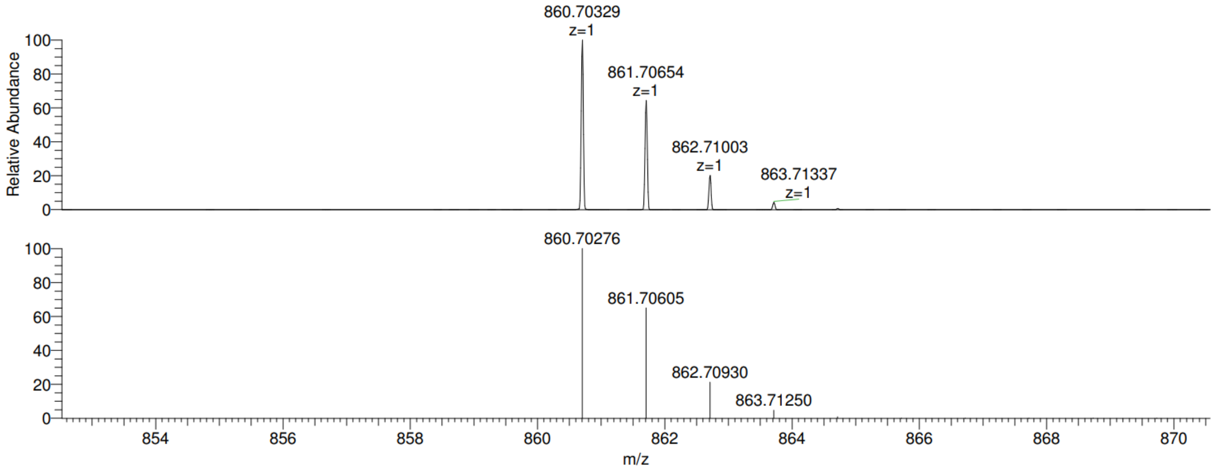

Figure S21. ESI-MS (MeOH) of **L^a-12**.

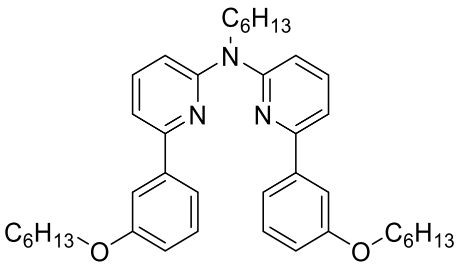

N-hexyl-N,N-bis(6-(3-(hexyloxy)phenyl)pyridine-2-yl)-amine (L^b-6)

2-6 (0.472 g, 1.14 mmol), $Pd(PPh_3)_4$ (0.160 g, 0.14 mmol,) and 3-hydroxyphenylboronic acid (0.474 g, 3.44 mmol) were placed in a 50 mL two-necked flask and dissolved in 25 mL of THF. The solution was purged by bubbling with Ar and heated to reflux. Then, aqueous K_2CO_3 (2 M, 4.5 mL, 9 mmol) was added with a syringe. The resulting yellow solution was reacted overnight. THF was removed under reduced pressure. The residue was transferred to a separation funnel with diluted HCl (5 mL), diluted with water (15 mL) and extracted with CH_2Cl_2 (25 mL x 3). The combined organic layers were washed with brine (15 mL) and dried with Na_2SO_4. The solution was concentrated and then filtered through a SiO_2 plug, washed with CH_2Cl_2/MeOH (100:1 and 100:2) while collecting the yellow solution. The solvent was evaporated and the crude product combined with K_2CO_3 (0.525 g, 3.80 mmol) in 25 mL of butanone in 50 mL two-necked flask. The mixture was purged by bubbling with Ar and heated up to reflux. Then, $C_6H_{13}Br$ (1.3 mL, 5.55 mmol) was added and the mixture reacted for 48 h. The mixture was cooled to room temperature and the solvent was removed under reduced pressure. The product was dissolved in CH_2Cl_2 (50 mL) and washed with water (25 mL) and brine (25 mL), dried with Na_2SO_4 and the solvent was evaporated. The product was purified by column chromatography (SiO_2) using hexane/toluene (100:1 to 50:1) to yield a white solid (0.145 g, 51 %).

^1H NMR (500 MHz, CD_2Cl_2): δ = 7.67 (dd, J = 2.6, 1.6 Hz, 2H), 7.65 – 7.57 (m, 4H), 7.39 – 7.32 (m, 4H), 7.18 (d, J = 8.2 Hz, 2H), 6.94 (dd, J = 8.2, 2.6, 2H), 4.39 (t, J = 8.1, 2H), 4.04 (t, J = 6.6 Hz, 4H), 1.94 – 1.85 (m, 2H), 1.87 – 1.77 (m, 4H), 1.56 – 1.43 (m, 6H), 1.43 – 1.27 (m, 12H), 0.94 (t, J = 7.0 Hz, 6H), 0.89 (t, J = 7.1 Hz, 3H).

^{13}C NMR (126 MHz, CD_2Cl_2): δ = 160.2, 157.5, 155.7, 141.4, 138.2, 130.0, 119.3, 115.9, 113.7, 113.5, 113.0, 68.6, 49.0, 32.4, 32.3, 29.9, 29.0, 27.7, 26.4, 23.4, 23.2, 14.4, 14.4.

EM-MS (ESI+, MeOH, m/z): calcd. for $[M + H]^+$ = 608.42105, found 608.42056

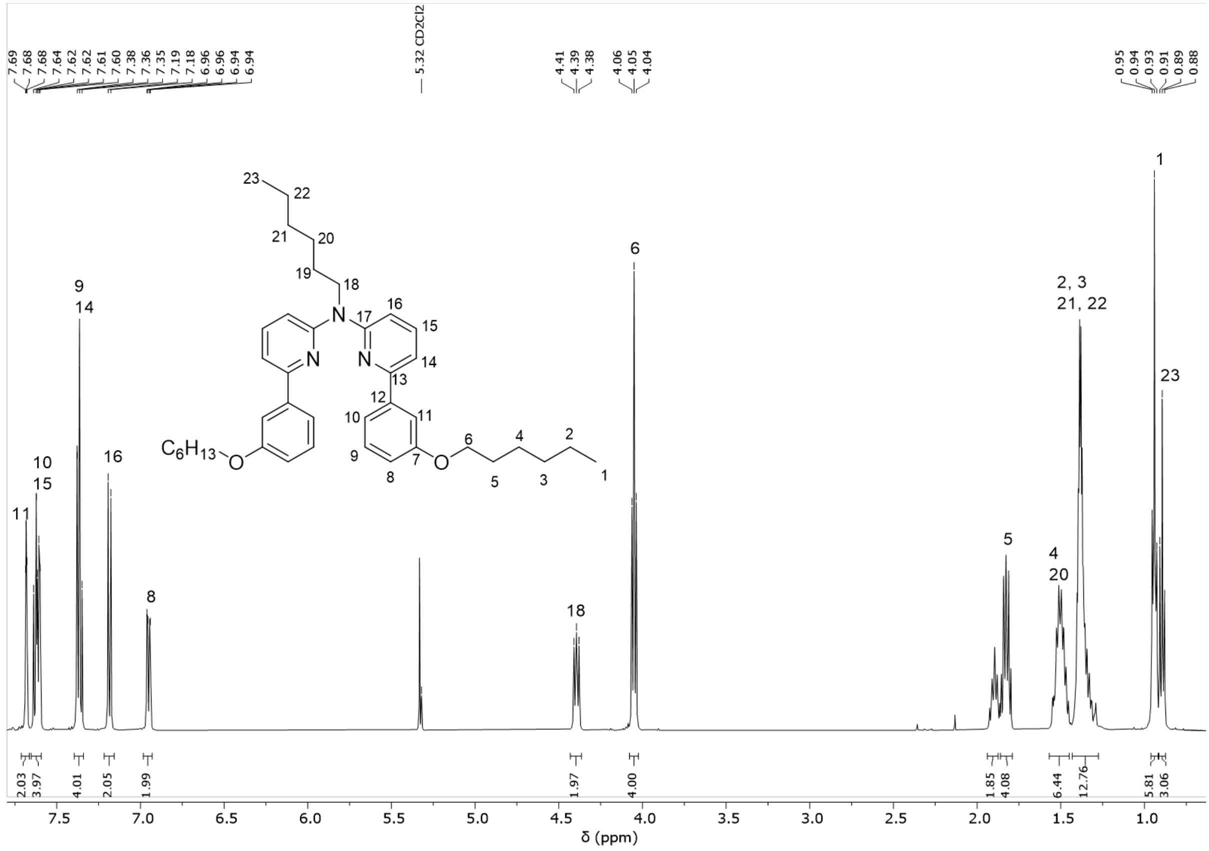

Figure S22. ^{1}H-NMR spectrum (500 MHz, CD_2Cl_2) of **L^b-6**.

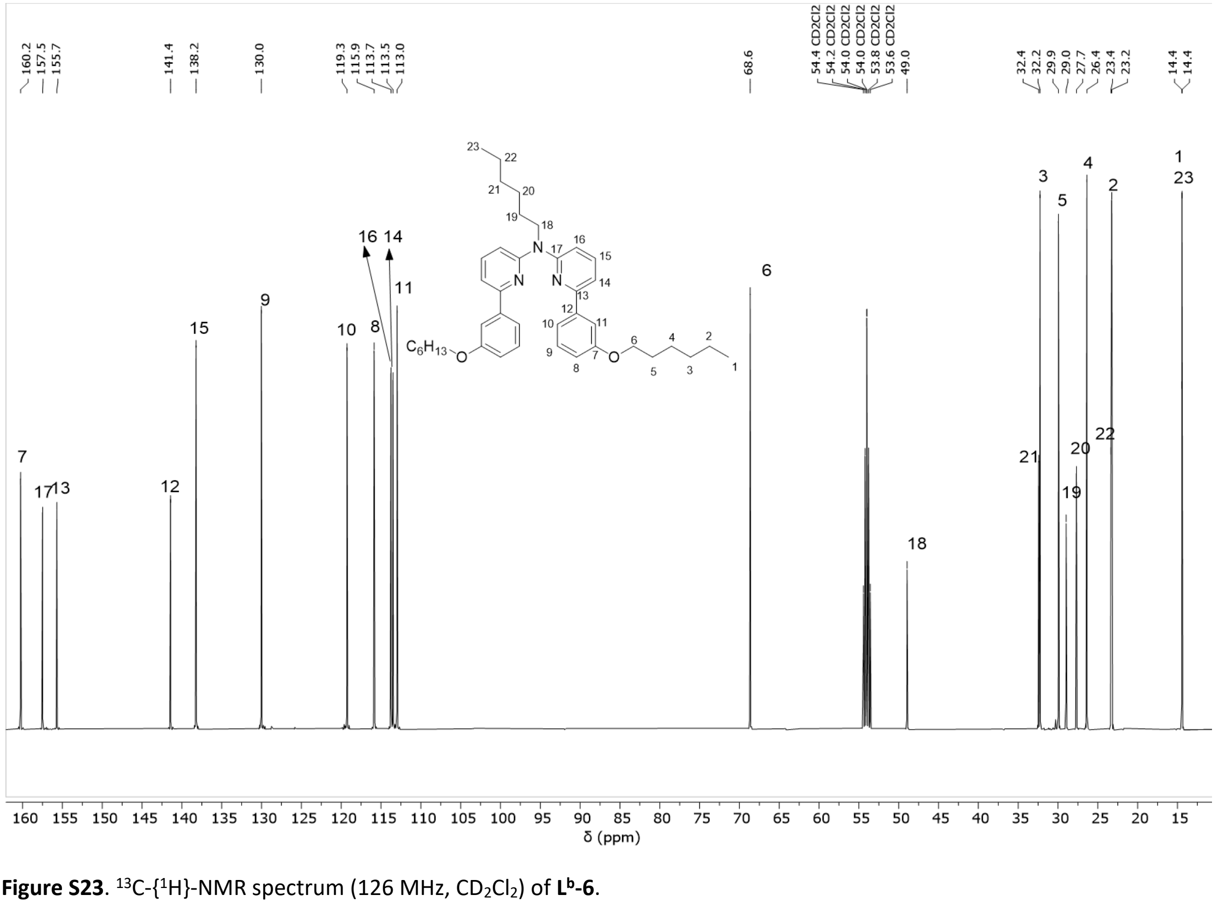

Figure S23. ^{13}C-{^{1}H}-NMR spectrum (126 MHz, CD_2Cl_2) of **L^b-6**.

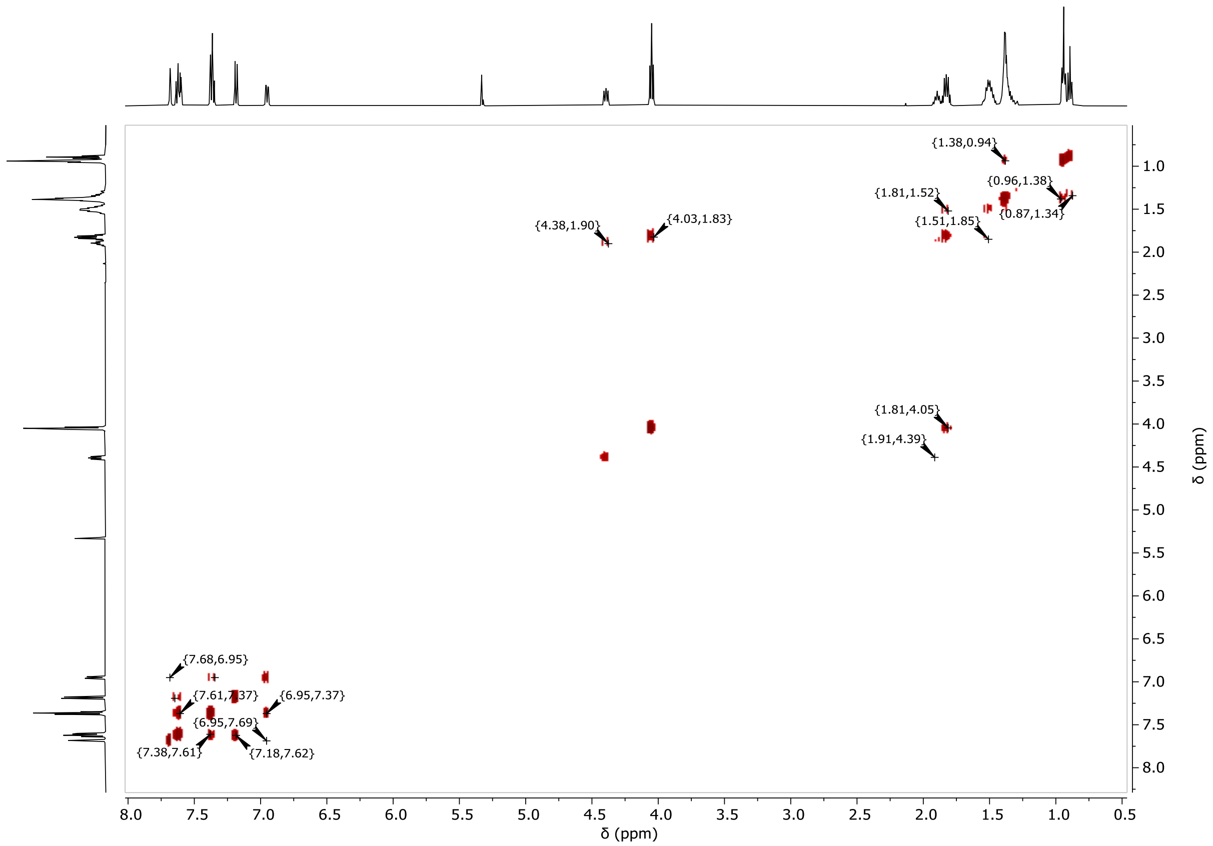

Figure S24. $^1H/^1H$-COSY-NMR spectrum (500 MHz/500 MHz, CD_2Cl_2) of **L^b-6.**

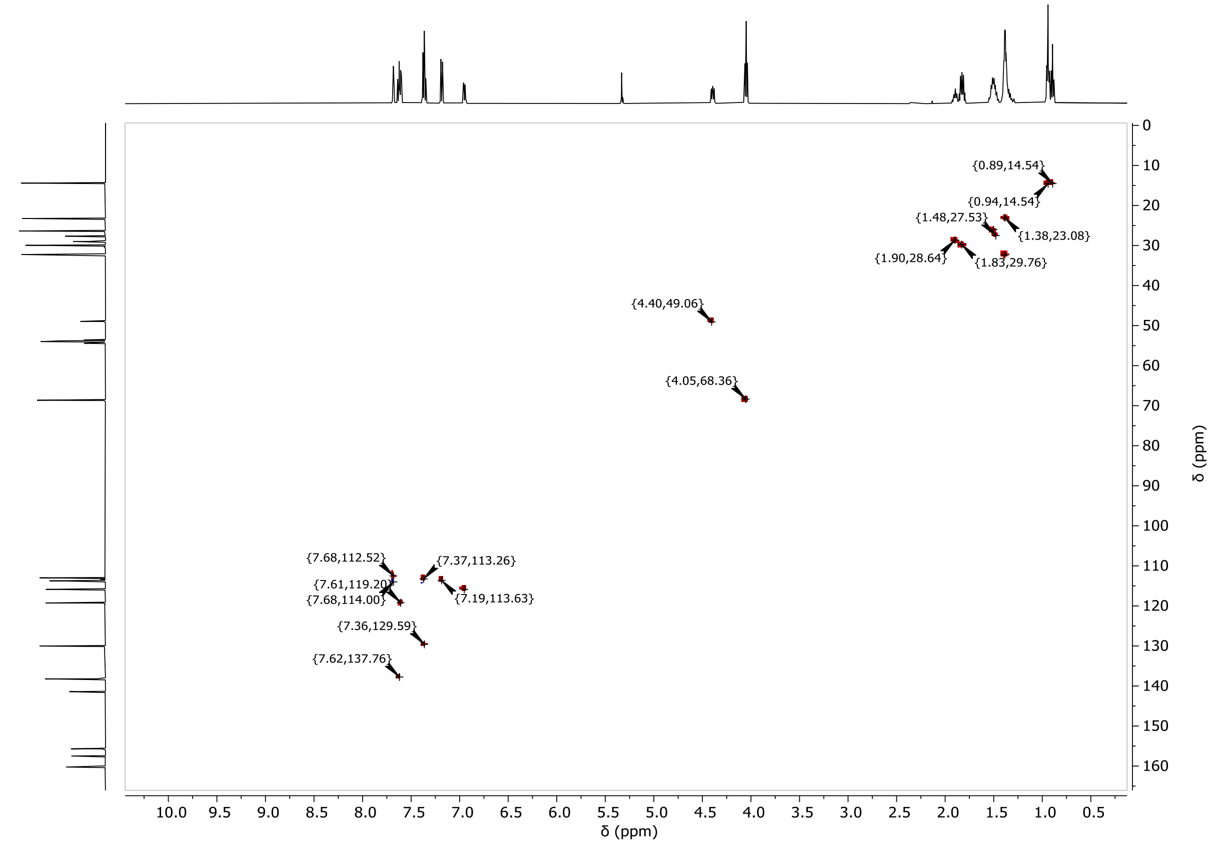

Figure S25. $^1H/^{13}C$-gHSQC-NMR spectrum (500 MHz/126 MHZ, CD_2Cl_2) of **L^b-6**.

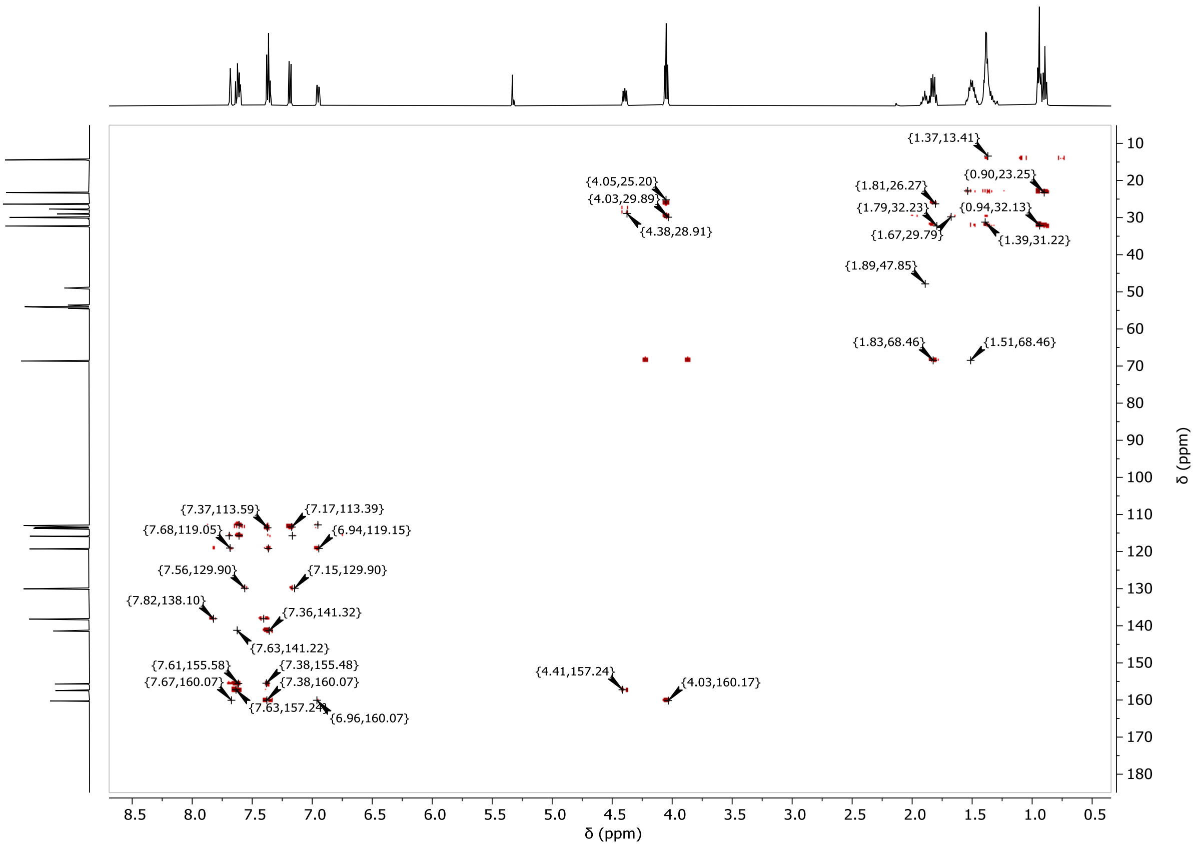

Figure S26. $^{1}H/^{13}C$-gHMBC-NMR spectrum (500 MHz/126 MHz, CD_2Cl_2) of **L^{b}-6**.

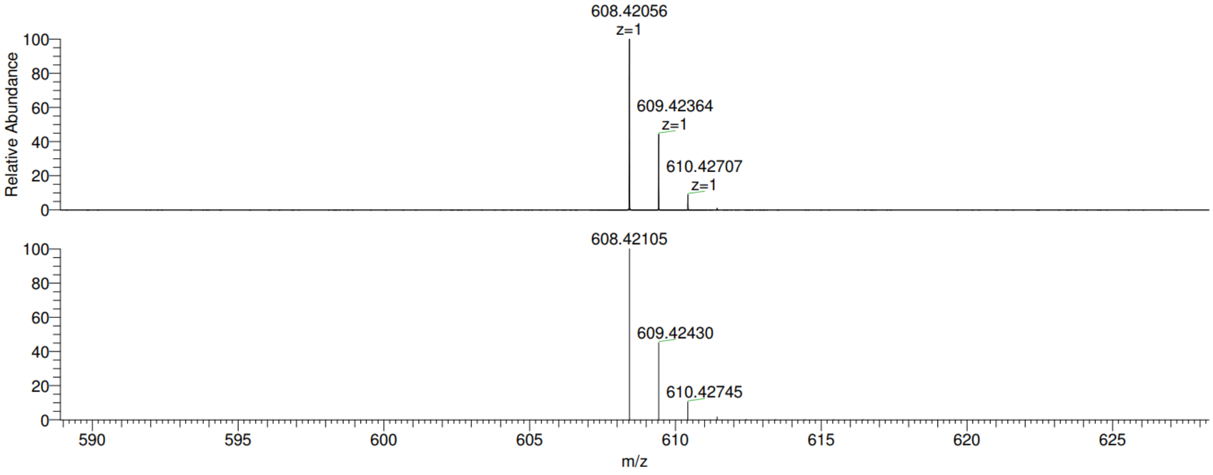

Figure S27. ESI-MS (MeOH) of **L^{b}-6**.

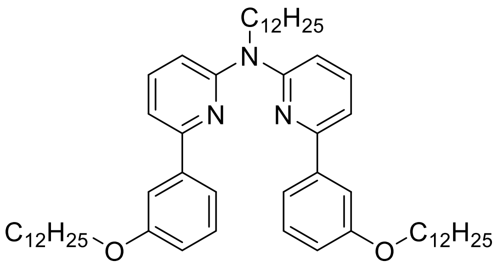

N-dodecyl-N,N-bis(6-(3-(dodeciloxy)phenyl)pyridine-2-yl)-amine (L^b-12)

2-12 (0.625 g, 1.25 mmol), $Pd(PPh_3)_4$ (0.176 g, 0.15 mmol,) and 3-hydroxyphenylboronic acid (0.411 g, 2.94 mmol) were placed in a 50 mL two-necked flask and dissolved in 25 mL THF. The solution was purged by bubbling with Ar and heated to reflux. Then, aqueous K_2CO_3 (2 M, 5 mL, 10 mmol) was added with a syringe. The resulting yellow solution was reacted overnight. THF was removed under reduced pressure. The residue was transferred to a separation funnel neutralized with HCl 1:4 (5mL), diluted with water (15 mL) and extracted with CH_2Cl_2 (25 mL x 3). The combined organic layers were washed with brine (15 mL) and dried with Na_2SO_4. The solution was concentrated and then filtered through a SiO_2 plug, washed with CH_2Cl_2/MeOH (100:1 and 100:2) while collecting the yellow solution. The solvent was evaporated and the crude product combined with K_2CO_3 (0.525 g, 3.80 mmol) in 25 mL of butanone in 50 mL two-necked flask. The mixture was purged by bubbling with Ar and heated up to reflux. Then, $C_{12}H_{25}Br$ (0.8 mL, 3.34 mmol) was added and the mixture reacted for 48 h. The mixture was cooled to room temperature and the solvent was removed under reduced pressure. The product was dissolved in CH_2Cl_2 (25 mL) and washed with water (25 mL) and brine (25 mL), dried with Na_2SO_4 and the solvent was evaporated. The product was purified by column chromatography (SiO_2) using hexane/toluene (3:1 to 1:1) to yield a white solid (0.728 g, 68%)

^{1}H NMR (400 MHz, CD_2Cl_2): δ = 7.67 (t, J = 2.1 Hz, 2H), 7.67 – 7.56 (m, 4H), 7.35 (m, 4H), 7.18 (d, J = 8.2 Hz, 2H), 6.94 (dd, J = 8.1, 2.6 Hz, 2H), 4.38 (t, J = 7.3 Hz, 2H), 4.04 (t, J = 6.6 Hz, 4H), 1.94 – 1.76 (m, 6H), 1.57 – 1.46 (m, 4H), 1.50 – 1.40 (m, 2H), 1.38 (s, 5H), 1.37 (dt, J = 16.5, 6.4 Hz, 4H), 1.25 (s, 9H), 0.89 (m, 9H).

^{13}C NMR (101 MHz, CD_2Cl_2): δ = 160.2, 157.5, 155.7, 141.4, 138.2, 130.0, 119.3, 115.9, 113.4, 113.5, 112.9, 68.7, 49.0, 32.6, 30.4, 30.4, 30.3, 30.3, 30.3, 30.3, 30.1, 30.0, 30.0, 29.0, 28.0, 26.7, 23.3, 14.5.

EM-MS (ESI+, MeOH, m/z) calcd. for $[M + H]^+$ = 860.70276, found 860.70303.

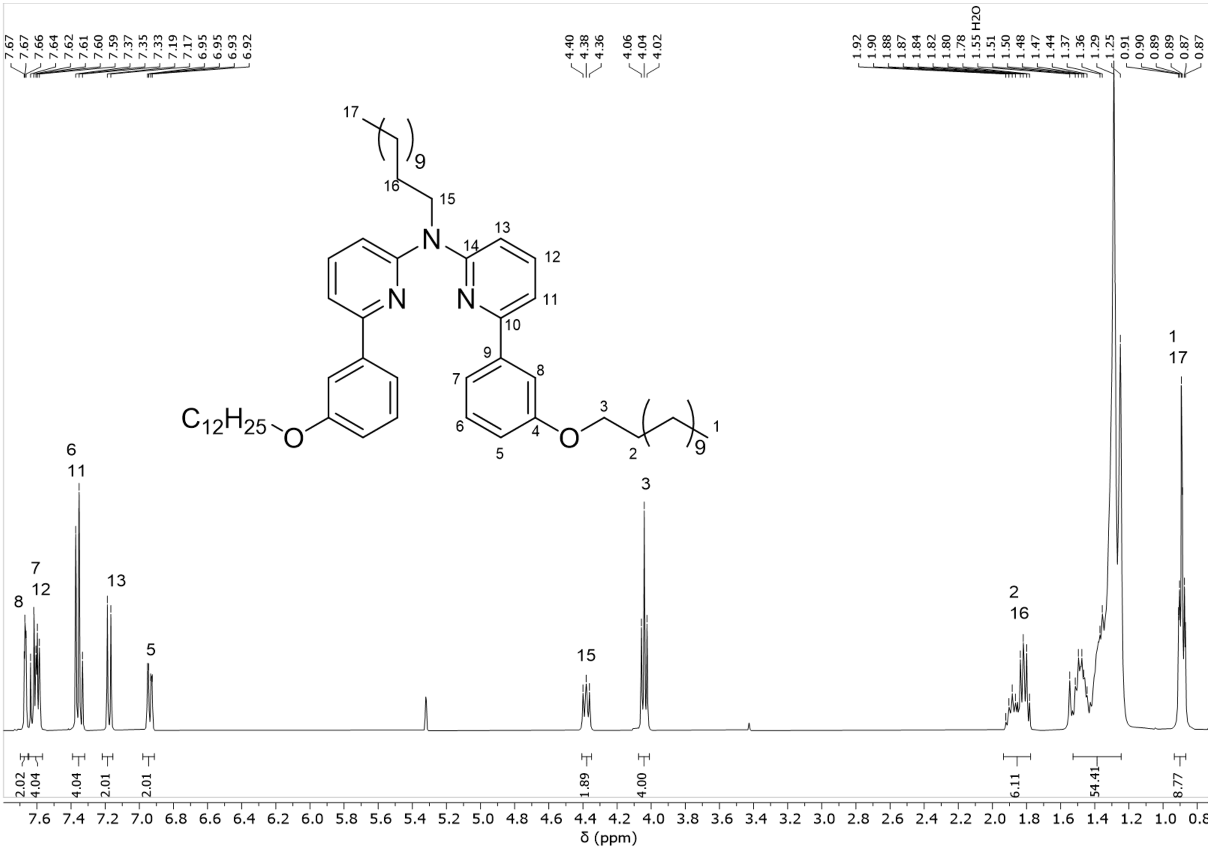

Figure S28. ^{1}H-NMR spectrum (400 MHz, CD_2Cl_2) of **L^{b}-12**.

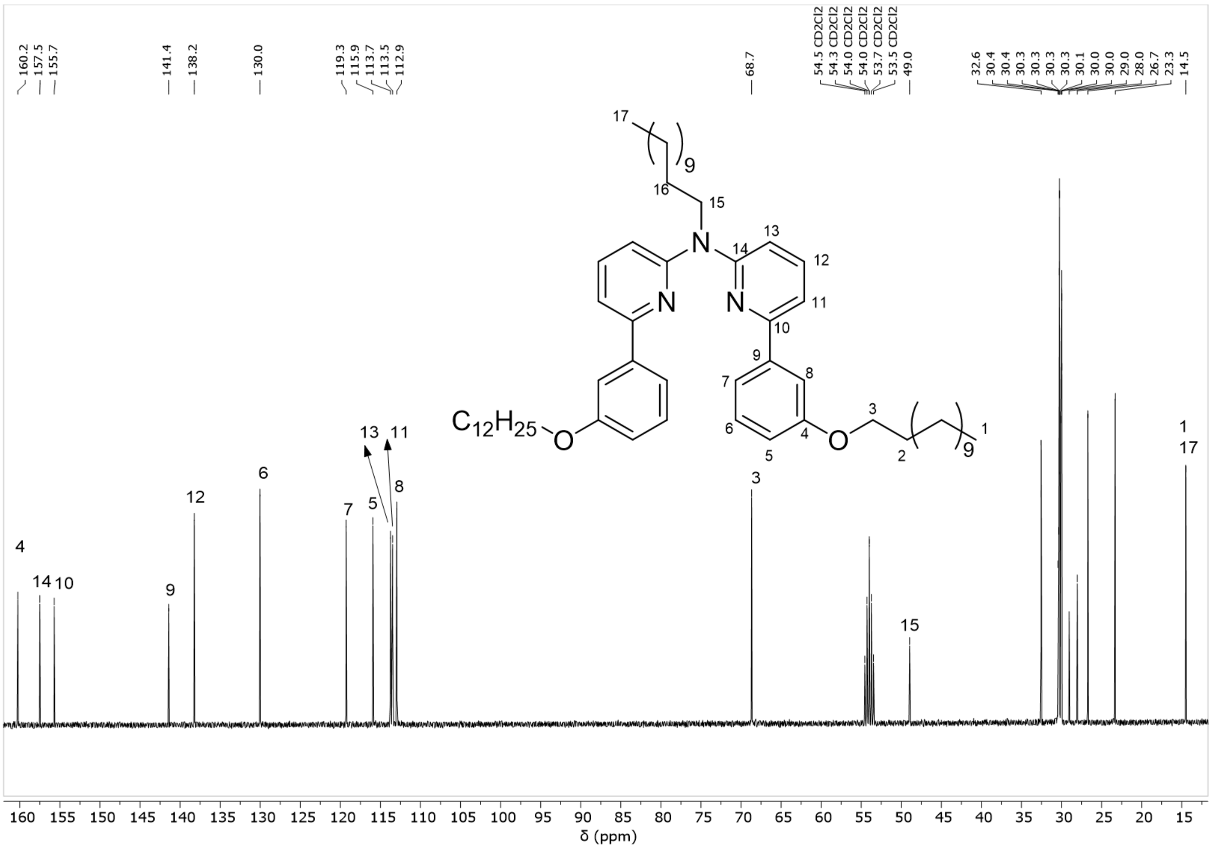

Figure S29. ^{13}C-{^{1}H}-NMR spectrum (101 MHz, CD_2Cl_2) of **L^{b}-12**.

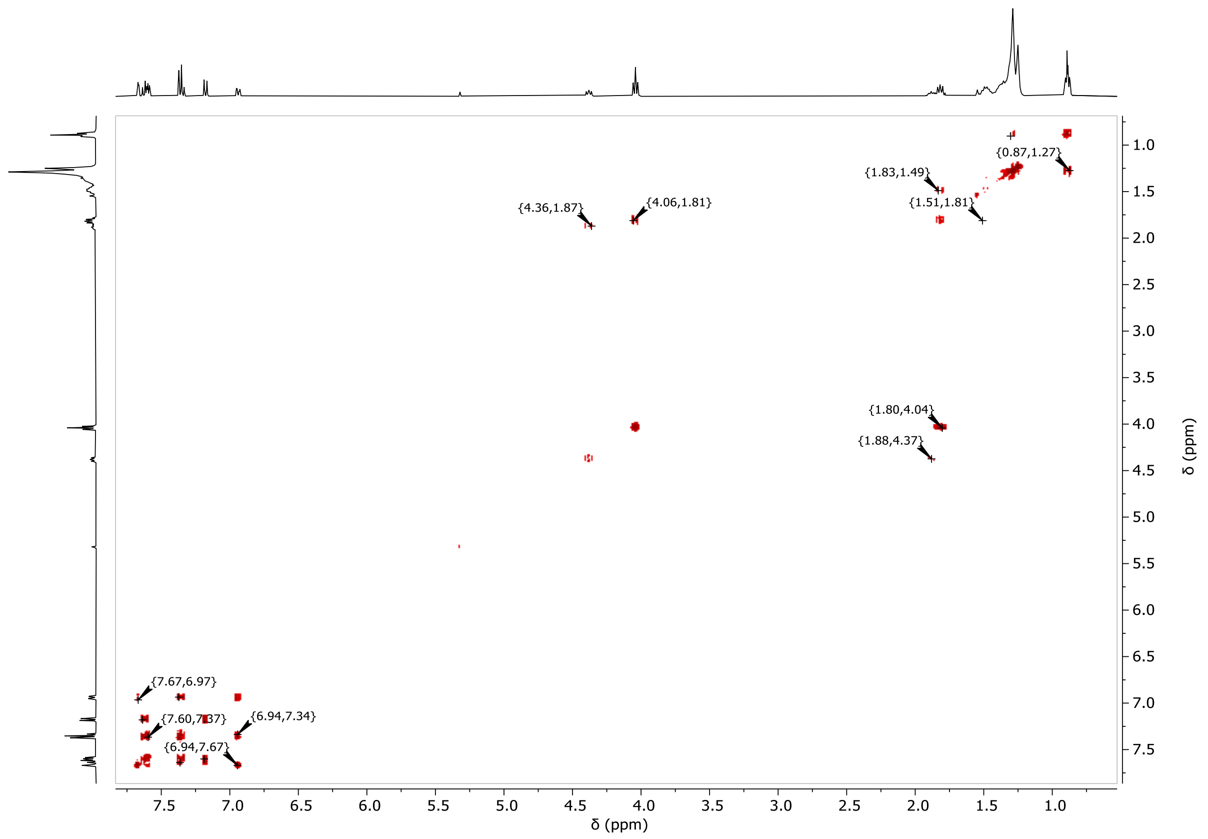

Figure S30. $^{1}H/^{1}H$-COSY-NMR spectrum (400 MHz/400 MHz, CD_2Cl_2) of **L**^{b}**-12.**

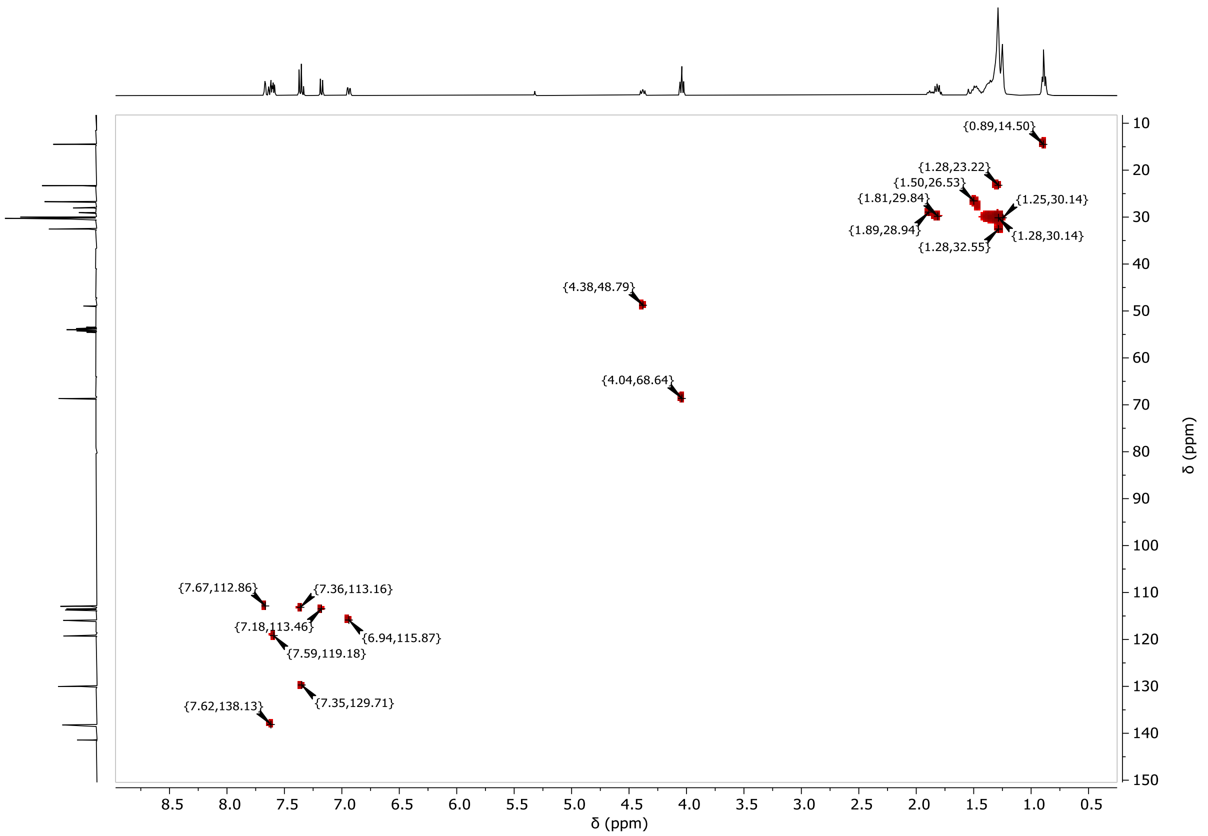

Figure S31. $^{1}H/^{13}C$-gHSQC-NMR spectrum (400 MHz/101 MHZ, CD_2Cl_2) of **L**^{b}**-12**.

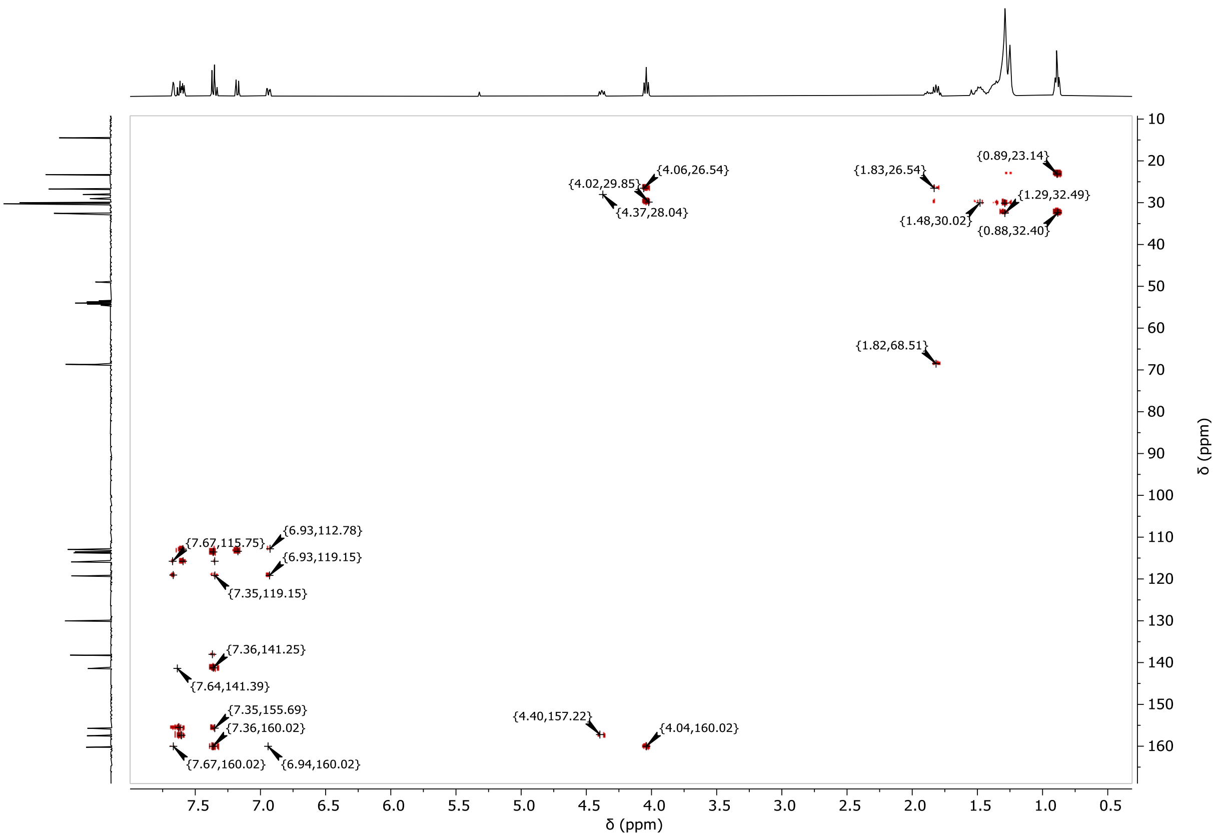

Figure S32. $^1H/^{13}C$-gHMBC-NMR spectrum (400 MHz/101 MHz, CD_2Cl_2) of **L^b-12**.

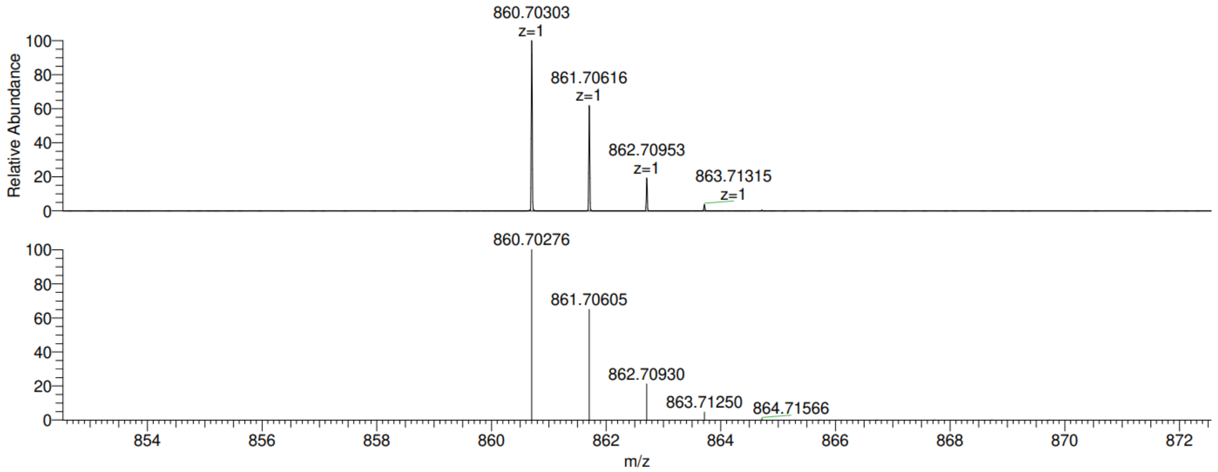

Figure S33. ESI-MS (MeOH) of **L^b-12**.

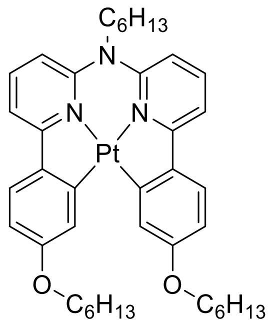

(N-hexyl-N,N-bis(6-(4-(hexyloxy)phenylido)pyridine-2-yl)-amine)-*$\kappa^4_{C,N,N,C}$* platinum(II) (PtL^a-6)

In a 50 mL two-necked flask, **L^a-6** (199 mg, 0.33 mmol) and K_2PtCl_4 (138 mg, 0.33 mmol) were suspended using 15 mL of glacial acetic acid. The mixture was purged by bubbling with Ar at room temperature for 15 min and the heated up to 130 °C under Ar flux. The mixture was reacted for 72 h. Then, the reaction mixture was cooled down to room temperature and the solvent removed under reduced pressure. The crude product was adsorbed onto silica gel and purified by column chromatography using cyclohexane/CH_2Cl_2 as eluent (3:1 to 1:1) to yield a yellow solid (103 mg, 39 %).

^{1}H NMR (400 MHz, CD_2Cl_2): δ = 7.90 (t, *J* = 8.1 Hz, 2H), 7.73 (d, *J* = 2.6 Hz, 2H), 7.67 (d, *J* = 8.6 Hz, 2H), 7.48 (dd, *J* = 7.9, 0.8 Hz, 2H), 7.17 (dd, *J* = 8.3, 0.8 Hz, 2H), 6.70 (dd, *J* = 8.5, 2.5 Hz, 2H), 4.21 (t, *J* = 7.3 Hz, 2H), 4.16 (t, *J* = 6.6 Hz, 4H), 1.90 – 1.82 (m, 4H), 1.80 – 1.70 (m, 2H), 1.43 – 1.35 (m, 8H), 1.31 – 1.24 (m, 2H), 1.22 – 1.17 (m, 4H), 0.93 (t, *J* = 7.1 Hz, 6H), 0.80 (t, *J* = 7.0 Hz, 3H).

^{13}C NMR (101 MHz, CD_2Cl_2): δ = 164.4, 160.4, 152.2, 151.4, 140.5, 138.1, 125.8, 120.2, 112.8, 112.0, 110.6, 68.2, 55.8, 54.4, 54.1, 53.8, 53.6, 53.3, 32.1, 31.6, 30.0, 28.9, 26.5, 26.3, 23.1, 22.8, 14.3, 14.1.

^{195}Pt NMR (86 MHz, CD_2Cl_2): δ -3546.

EM-MS (ESI+, MeOH, m/z) calcd. for $[M + H]^+$ = 801.37052, found 801.37091; $[M + Na]^+$ = 823.35247, found 823.35278.

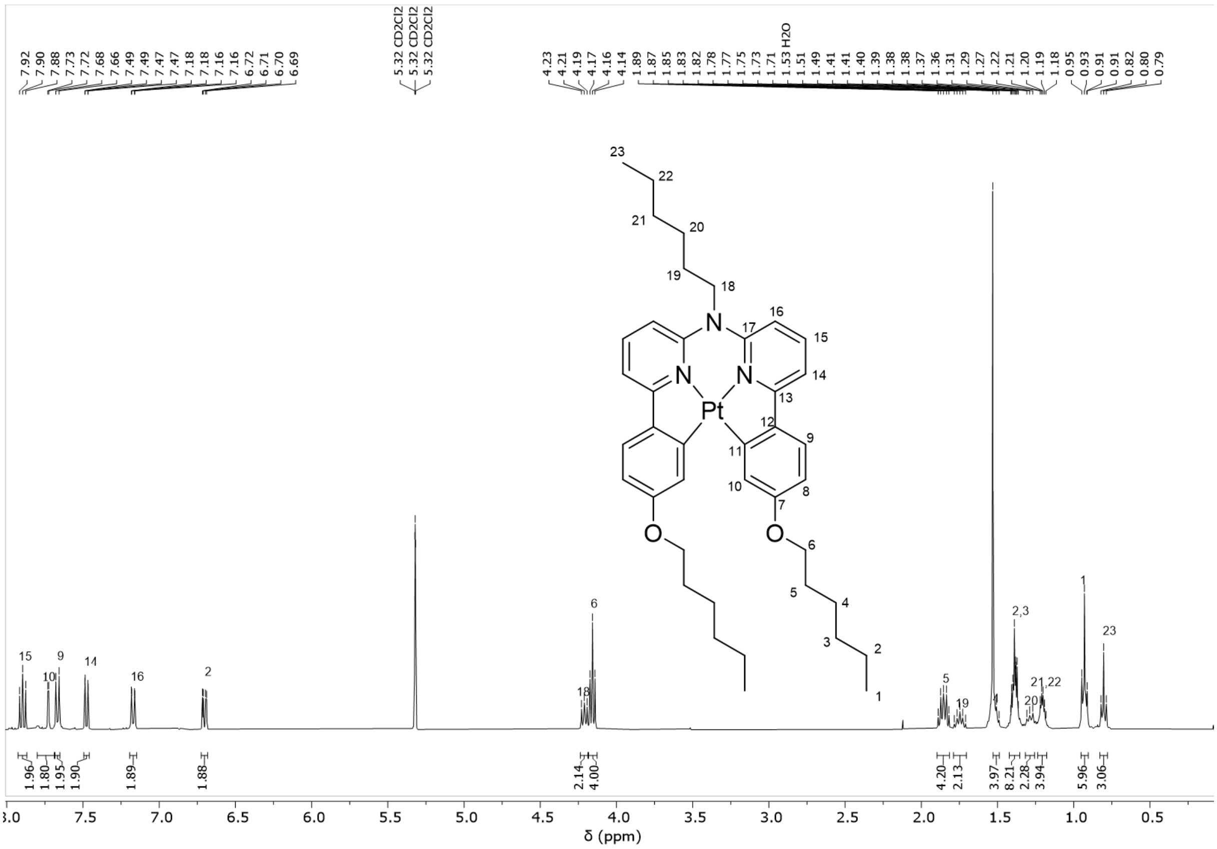

Figure S34. ^{1}H-NMR spectrum (400 MHz, CD_2Cl_2) of **PtL^{a}-6**.

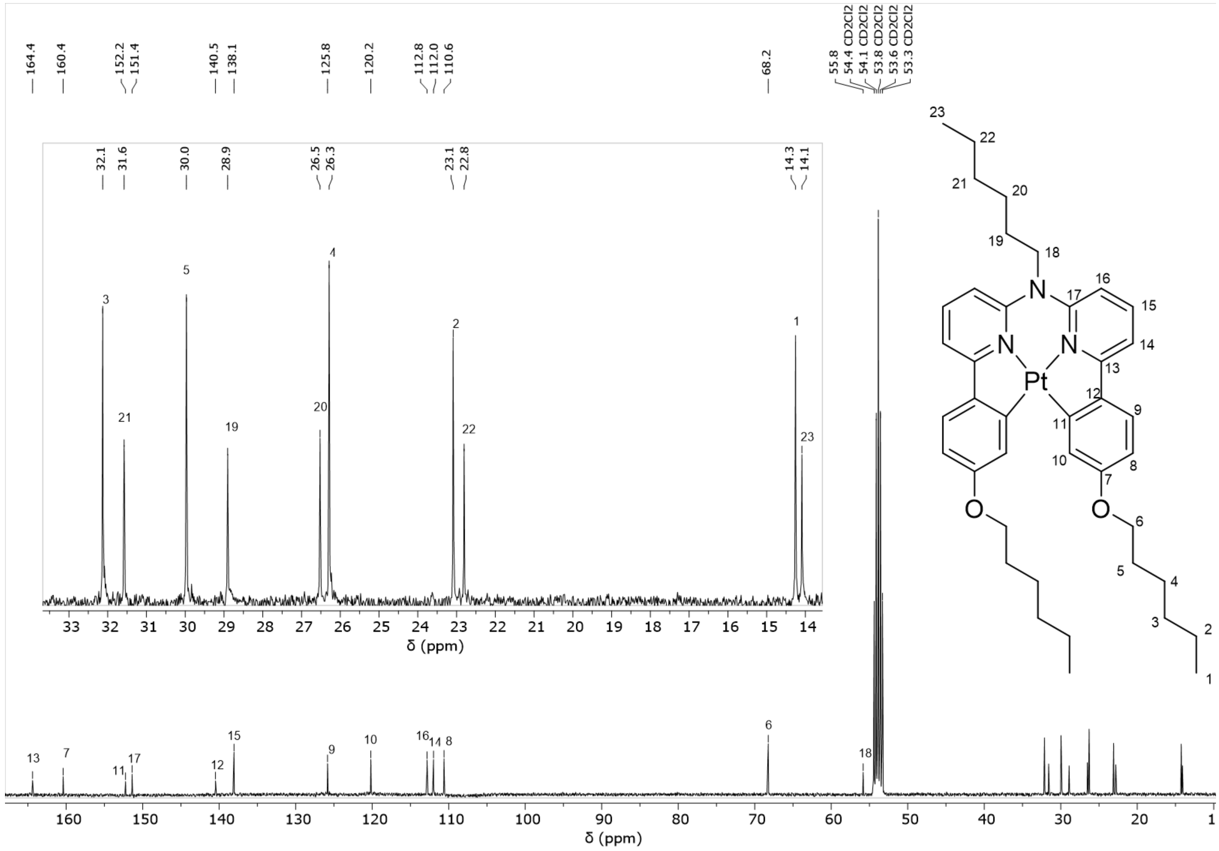

Figure S35. ^{13}C-{^{1}H}-NMR spectrum (101 MHz, CD_2Cl_2) of **PtL^{a}-6**.

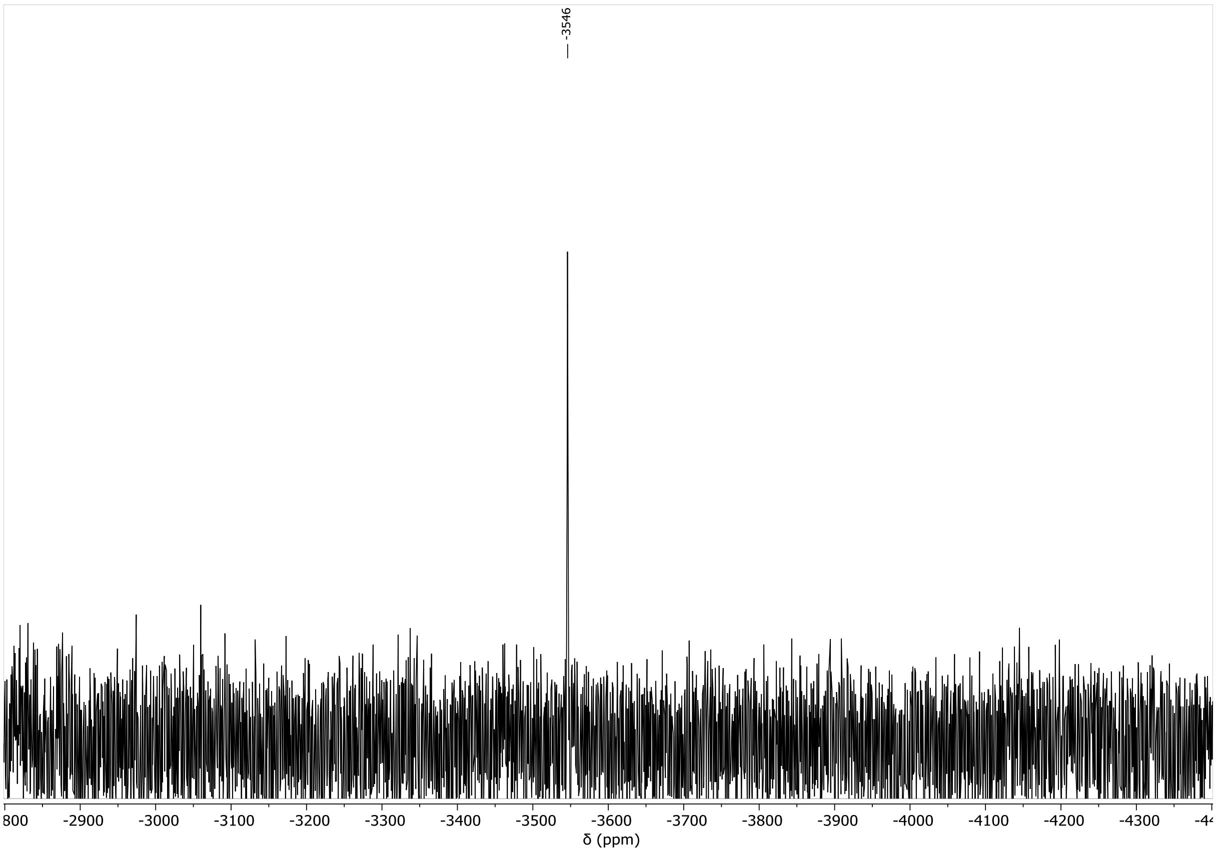

Figure S36. ^{195}Pt-NMR spectrum (86 MHz, CD_2Cl_2) of **PtL[a]-6**.

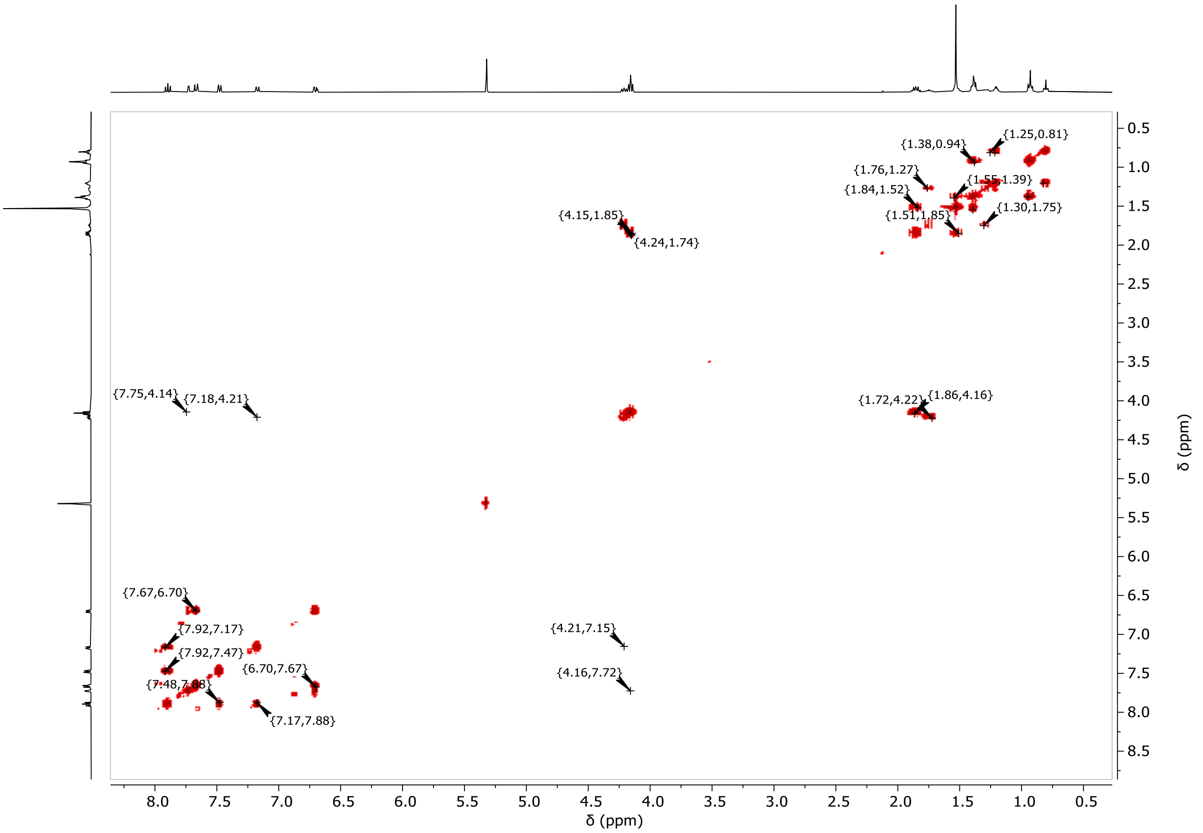

Figure S37. ^{1}H/^{1}H-COSY-NMR spectrum (400 MHz/400 MHz, CD_2Cl_2) of **PtL[a]-6.**

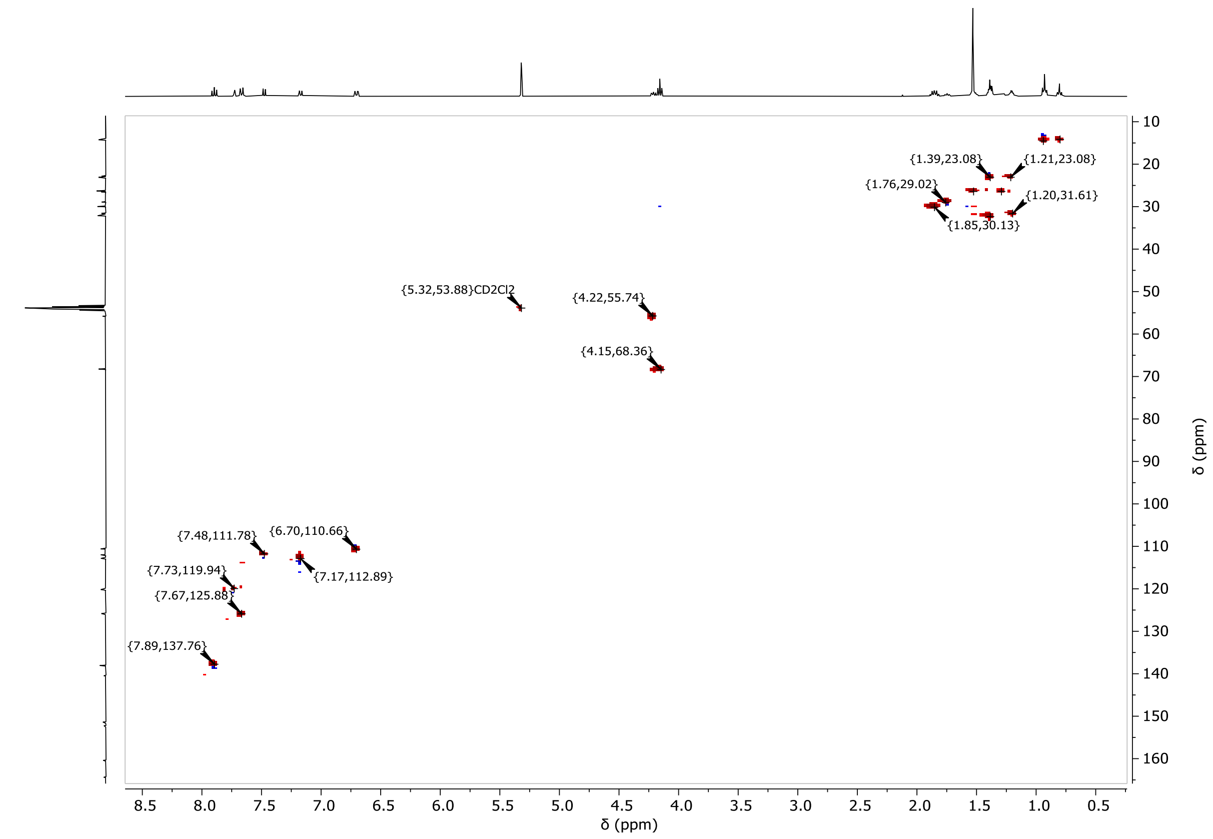

Figure S38. $^{1}H/^{13}C$-gHSQC-NMR spectrum (400 MHz/101 MHZ, CD_2Cl_2) of **PtL^a-6**.

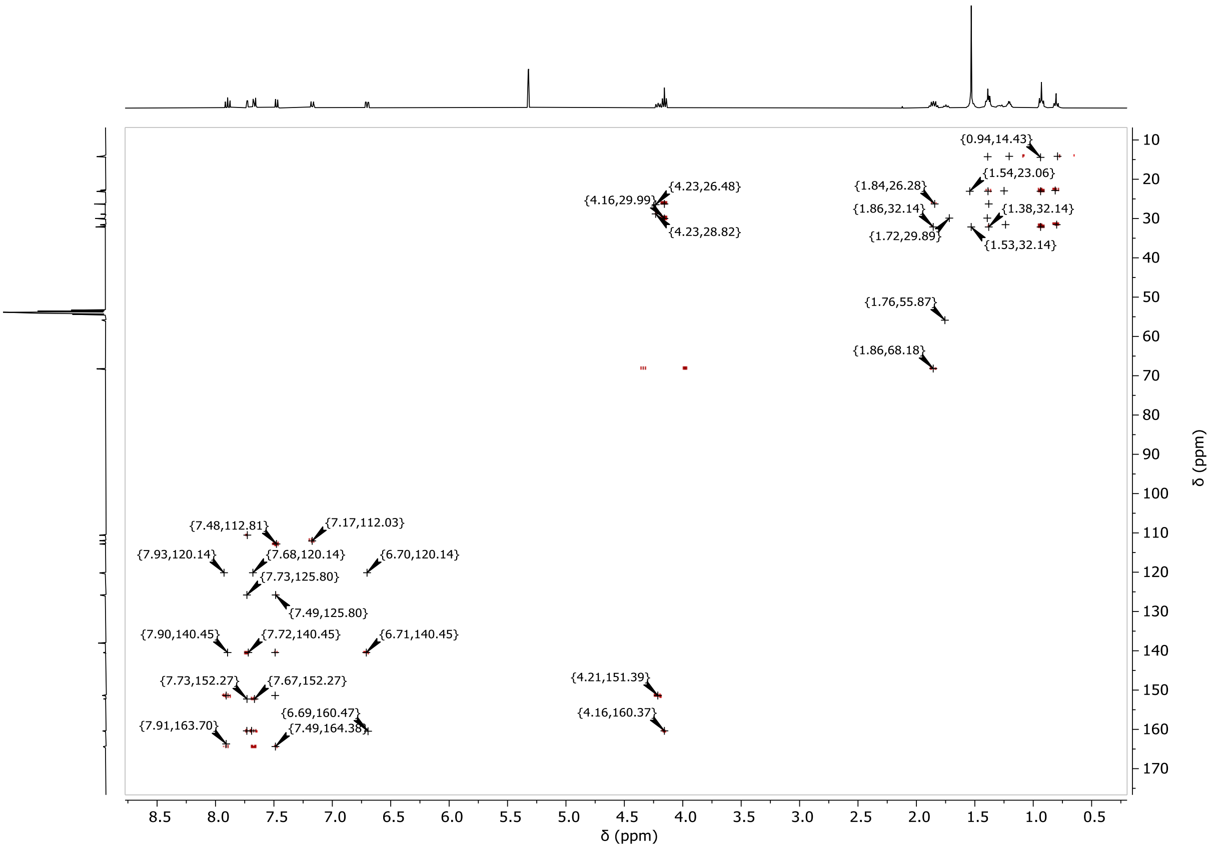

Figure S39. $^{1}H/^{13}C$-gHMBC-NMR spectrum (400 MHz/101 MHz, CD_2Cl_2) of **PtL^a-6**.

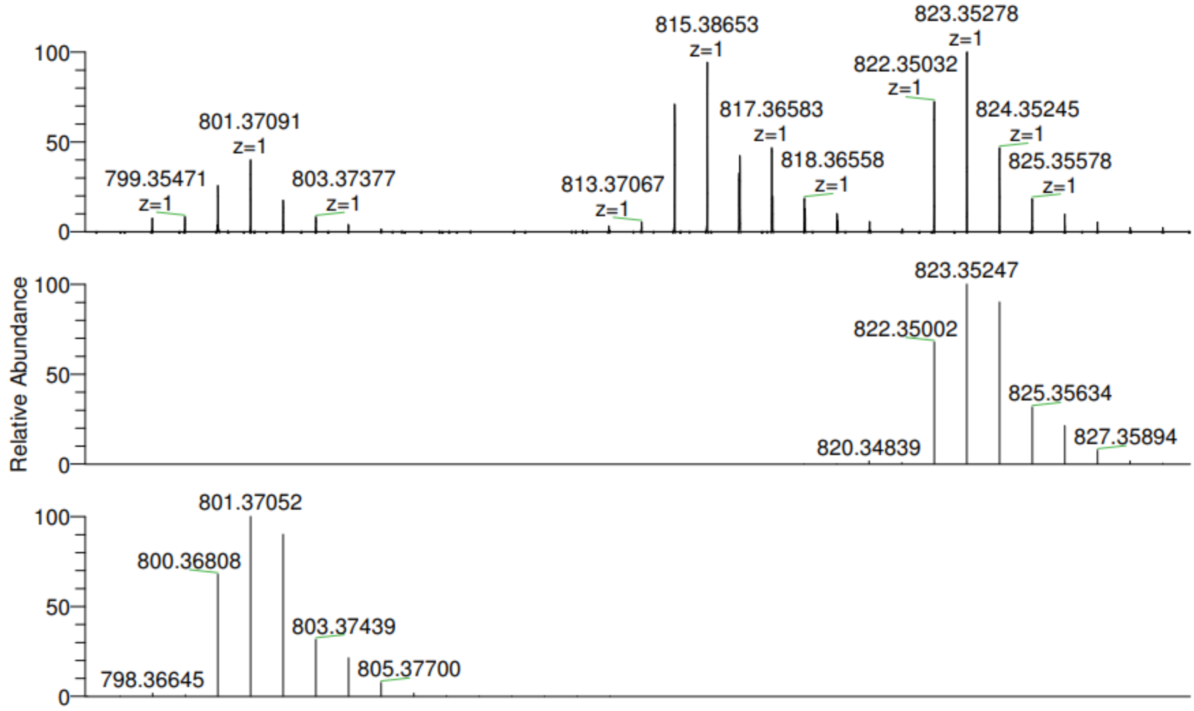

Figure S40. ESI-MS (MeOH) of **PtLª-16**.

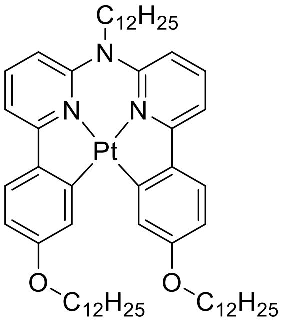

(N-dodecyl-N,N-bis(6-(4-(dodeciloxy)phenylido)pyridine-2-yl)-amine)-*κ*^{4}*$_{C,N,N,C}$* platinum(II) (PtLª-12)

In a 50 mL two-necked flask, **Lª-12** (203 mg, 024 mmol) and K_2PtCl_4 (99 mg, 0.24 mmol) were suspended using 15 mL of glacial acetic acid. The mixture was purged by bubbling with Ar at room temperature for 15 min and then heated up to 130 °C under Ar flux. The mixture was reacted for 72 h. Then, the mixture was cooled down to room temperature and the solvent removed under reduced pressure. The crude product was adsorbed onto silica gel and purified by column chromatography using cyclohexane/CH_2Cl_2 as eluent (3:1 to 1:1) to yield a yellow solid (107 mg, 43 %).

^{1}H NMR (500 MHz, CD_2Cl_2): δ = 7.89 (t, *J* = 8.1 Hz, 2H), 7.73 (d, *J* = 2.5 Hz, 2H), 7.66 (d, *J* = 8.6 Hz, 2H), 7.47 (d, *J* = 7.4 Hz, 2H), 7.16 (d, *J* = 7.9 Hz, 2H), 6.70 (dd, *J* = 8.5, 2.5 Hz, 2H), 4.20 (t, *J* = 7.3 Hz, 2H), 4.15 (t, *J* = 6.6 Hz, 4H), 1.90 – 1.82 (m, 2H), 1.77 – 1.71 (m, 2H), 1.53 – 1.47 (m, 6H), 1.44 – 1.14 (m, 50H), 0.88 (m, 9H).

^{13}C NMR (126 MHz, CD_2Cl_2): δ = 164.5, 160.5, 152.4, 151.5, 140.6, 138.2, 126., 120.2, 113.0, 112.1, 110.8, 68.4, 56.0, 32.5, 32.5, 30.3, 30.3, 30.3, 30.2, 30.2, 30.1, 30.0, 29.9, 29.5, 29.0, 26.9, 26.8, 23.3, 23.3, 14.5.

^{195}Pt NMR (107 MHz, CD_2Cl_2): δ = -3547.

EM-MS (ESI+, MeOH, m/z) calcd. for $[M + H]^+$ = 1053.65234, found 1053.65474.

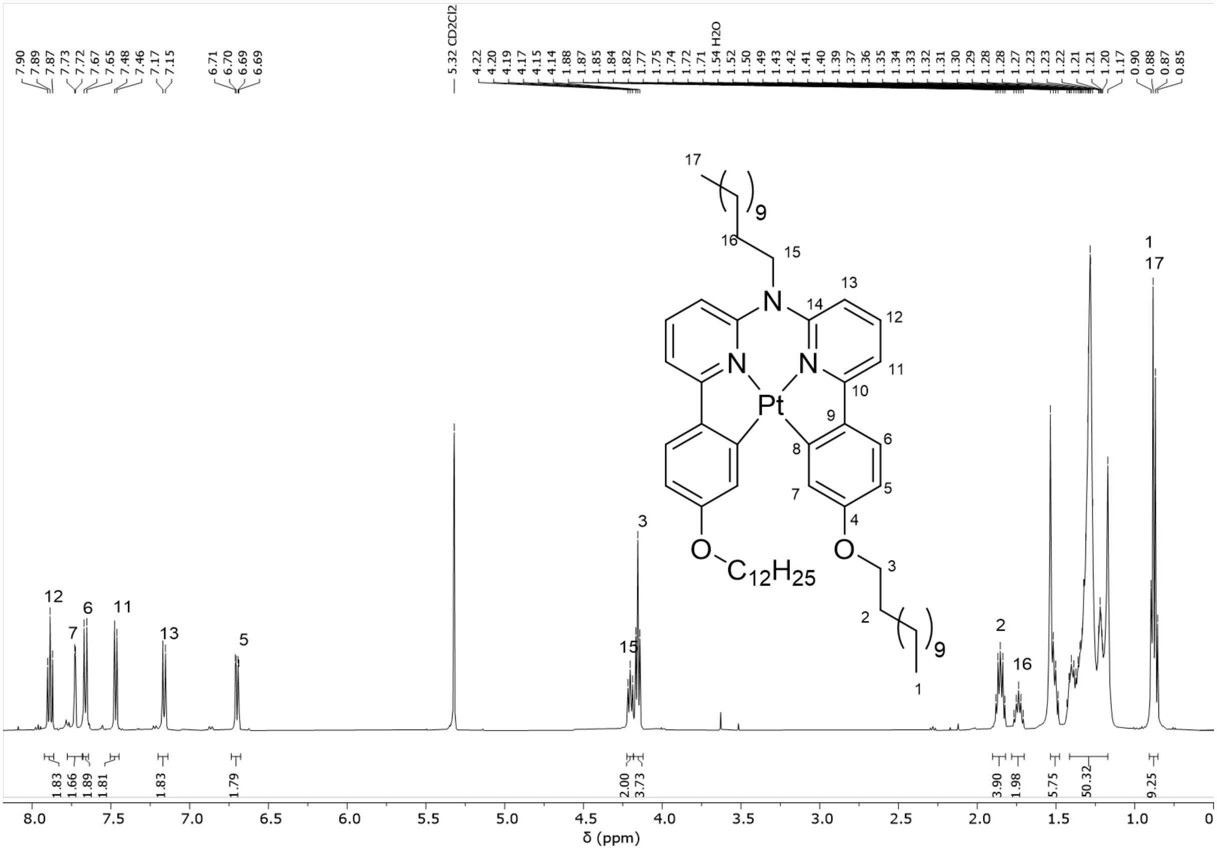

Figure S41. ^{1}H-NMR spectrum (500 MHz, CD_2Cl_2) of **PtL^{a}-12**.

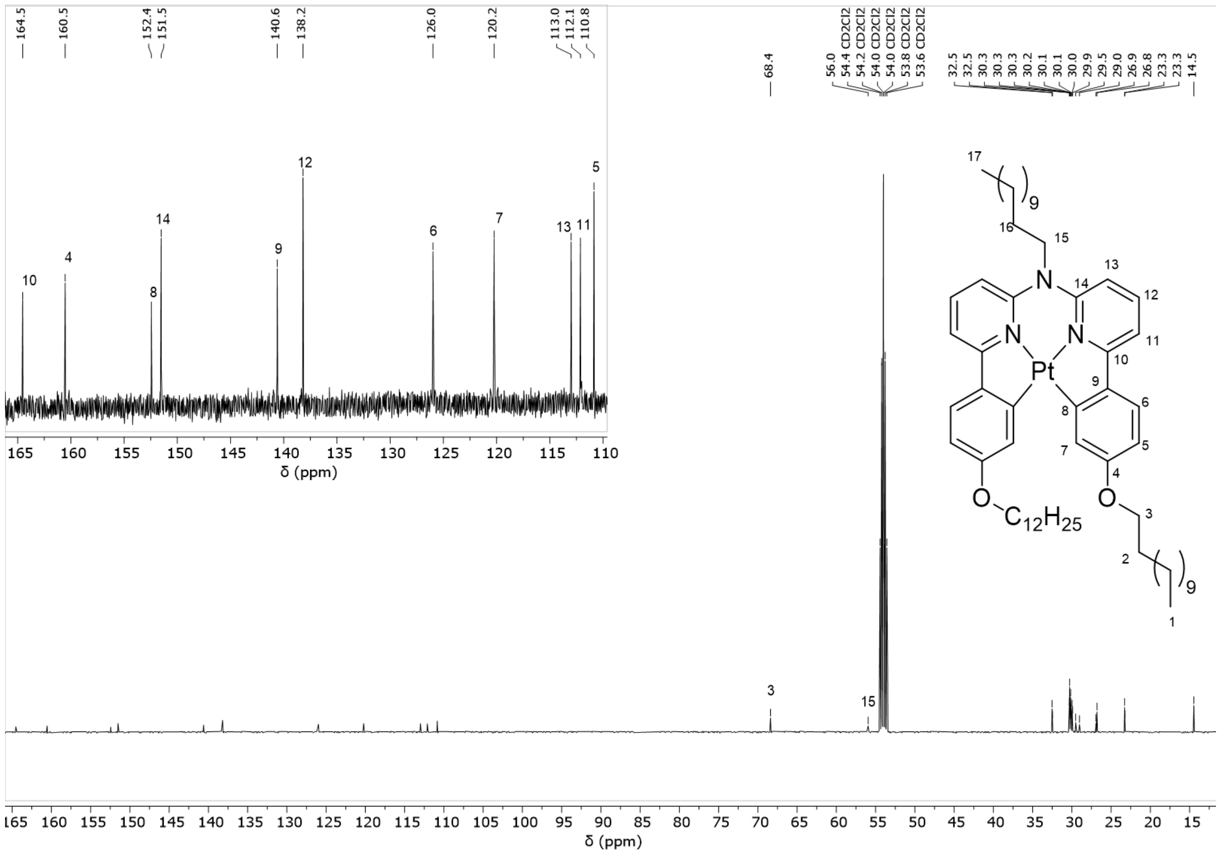

Figure S42. ^{13}C-{^{1}H}-NMR spectrum (126 MHz, CD_2Cl_2) of **PtL^{a}-12**.

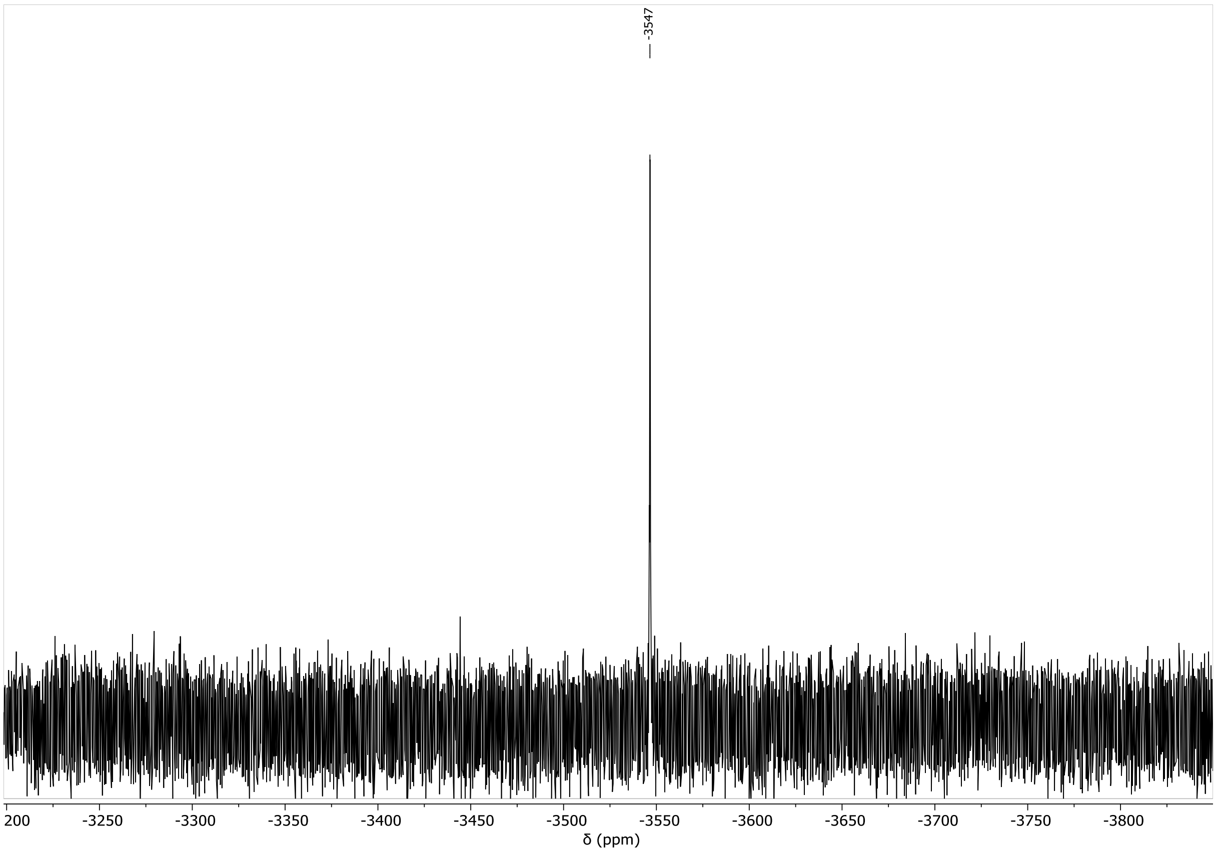

Figure S43. ^{195}Pt-NMR spectrum (107 MHz, CD_2Cl_2) of **PtL^a-12**.

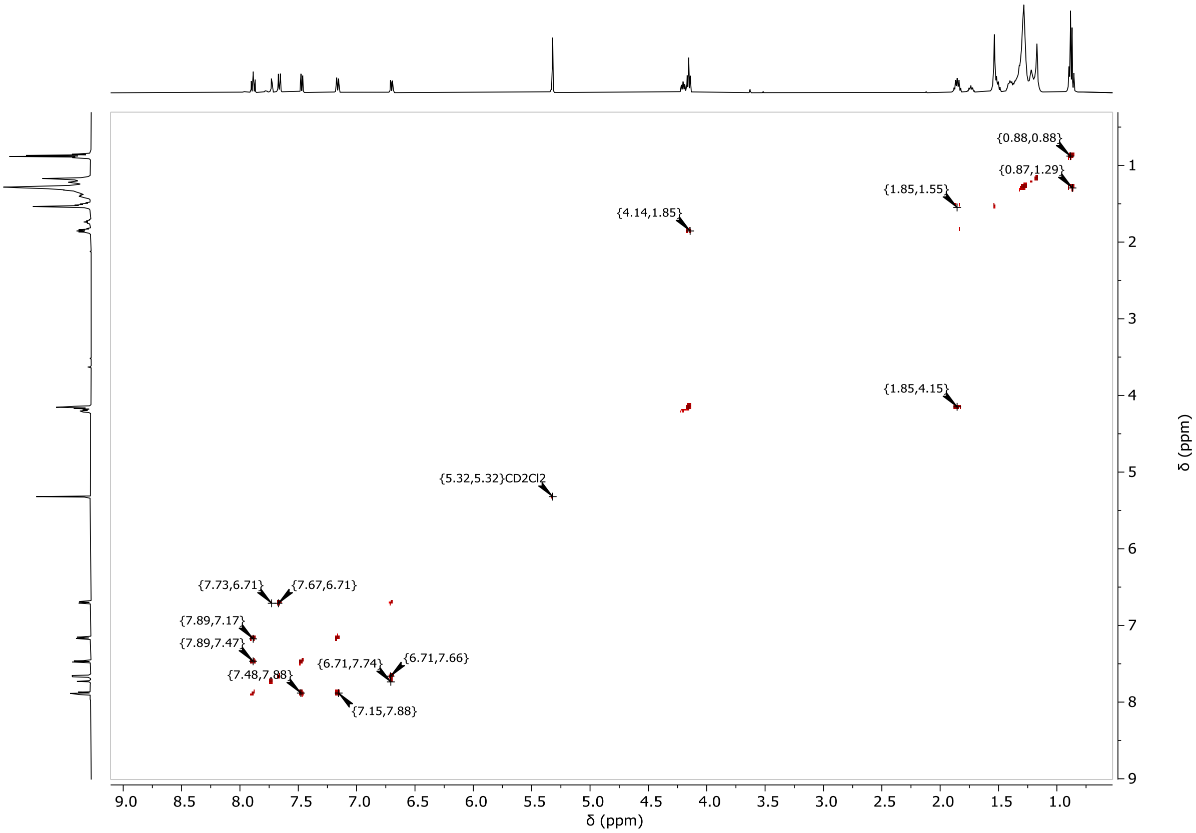

Figure S44. $^1H/^1H$-COSY-NMR spectrum (500 MHz/500 MHz, CD_2Cl_2) of **PtL^a-12.**

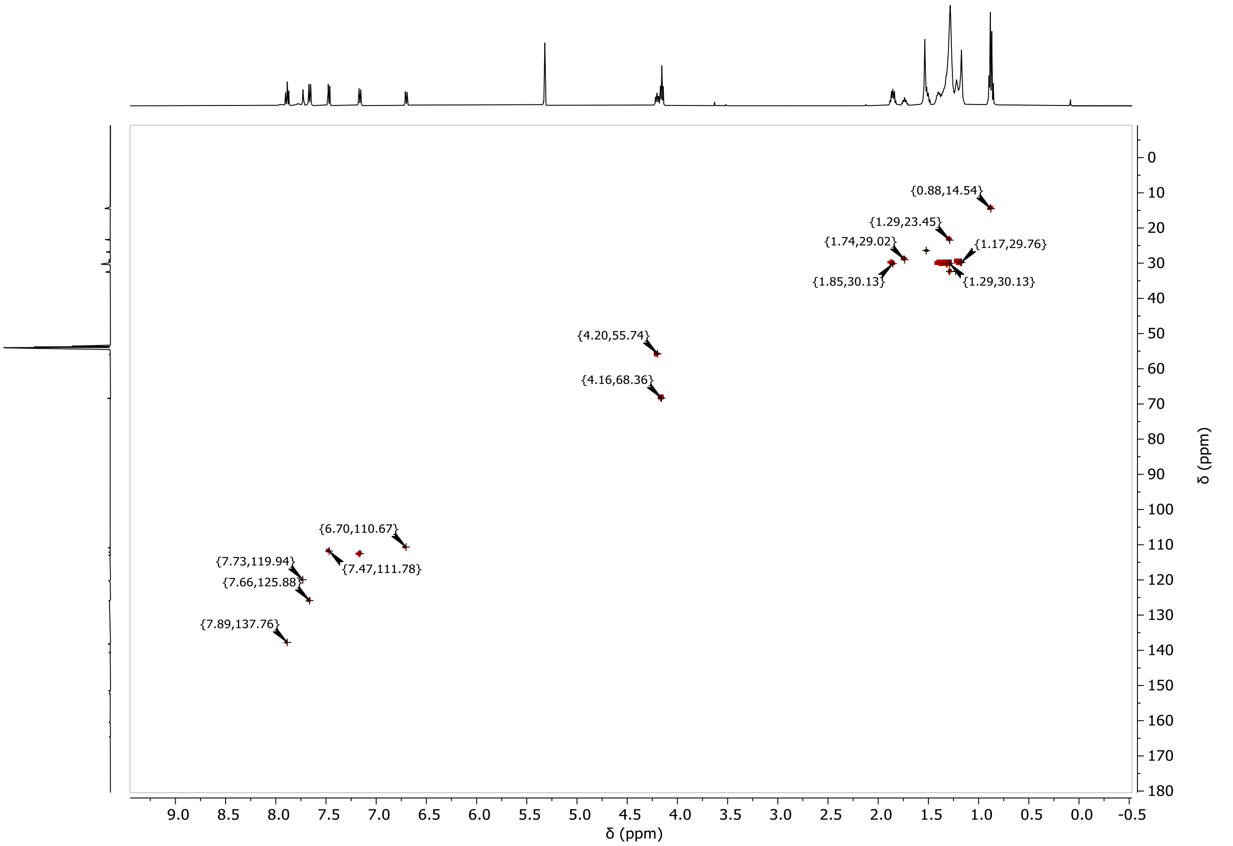

Figure S45. $^{1}H/^{13}C$-gHSQC-NMR spectrum (500 MHz/126 MHZ, CD_2Cl_2) of **PtL^{a}-12**.

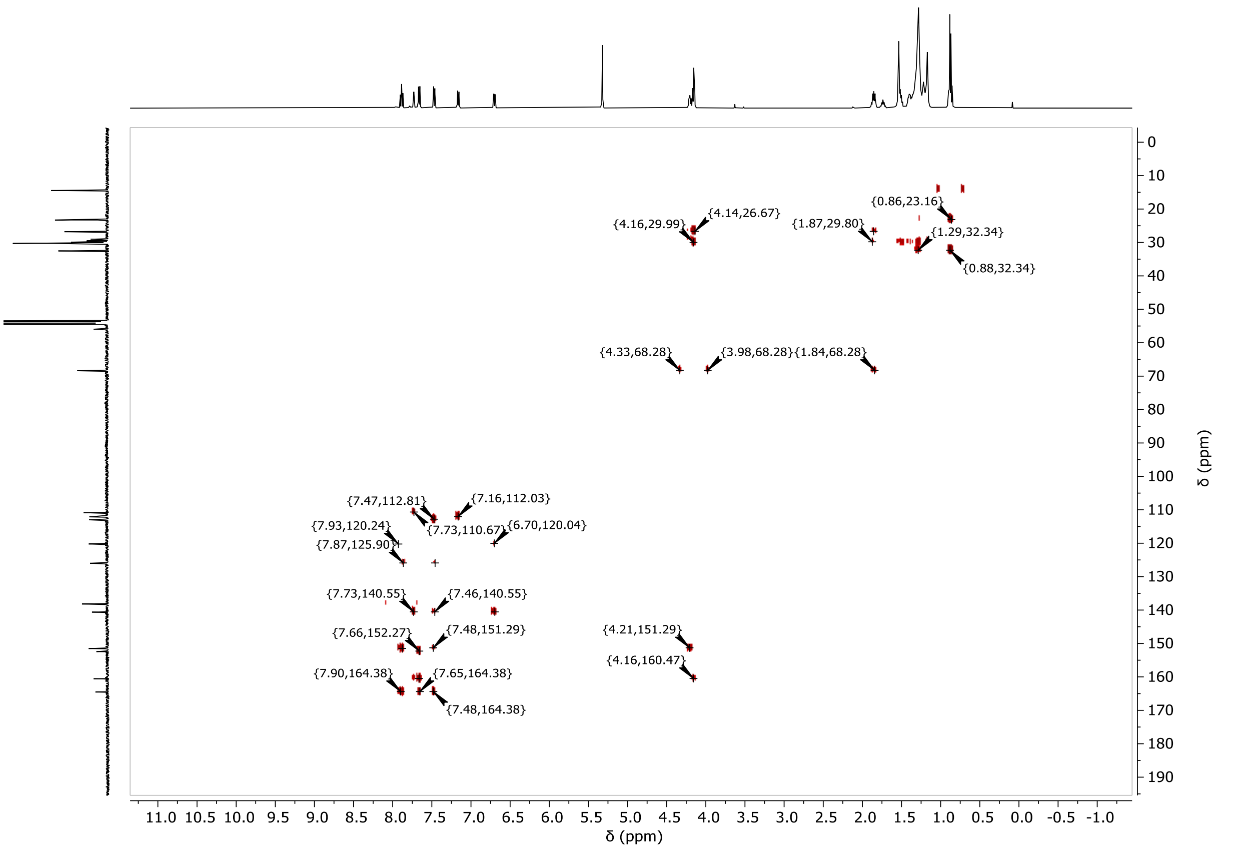

Figure S46. $^{1}H/^{13}C$-gHMBC-NMR spectrum (500 MHz/126 MHz, CD_2Cl_2) of **PtL^{a}-12**.

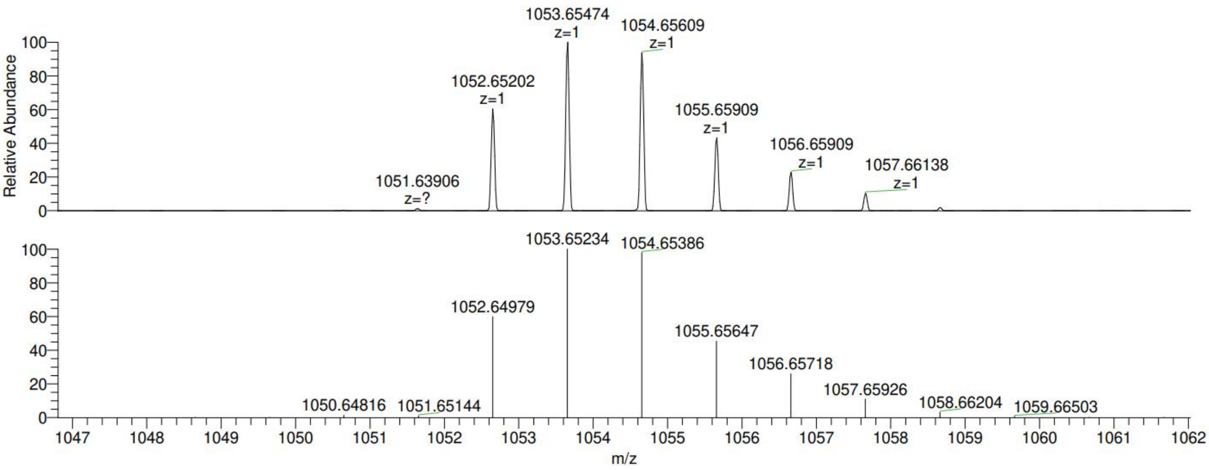

Figure S47. ESI-MS (MeOH) of **PtL^a-12**.

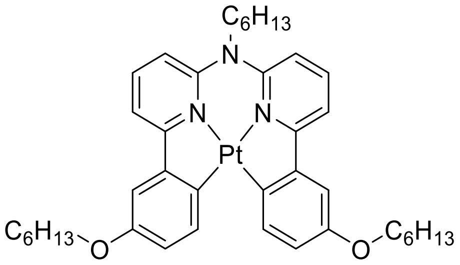

(N-hexyl-N,N-bis(6-(3-(hexyloxy)phenylido)pyridine-2-yl)-amine)-$\kappa^4_{C,N,N,C}$ platinum(II) PtL^b-6

In a 50 mL two-necked flask, **L^b-6** (208 mg, 0.34 mmol) and K_2PtCl_4 (144 mg, 0.34 mmol) were suspended using 15 mL of glacial acetic acid. The mixture was purged by bubbling with Ar at room temperature for 15 min and then heated up to 130 °C under Ar flux. The mixture was reacted for 72 h. The mixture was cooled down to room temperature and the solvent was removed under reduced pressure. The crude product was adsorbed onto silica gel and purified by column chromatography using cyclohexane/CH_2Cl_2 as eluent (3:1 to 1:1) to yield a yellow plastic solid (80 mg, 30 %).

^{1}H NMR (500 MHz, CD_2Cl_2): δ = 8.05 (d, J = 8.3 Hz, 2H), 7.94 (t, J = 8.1 Hz, 2H), 7.55 (d, J = 7.7 Hz, 2H), 7.30 (d, J = 2.8 Hz, 2H), 7.23 (d, J = 8.5 Hz, 2H), 7.00 (dd, J = 8.3, 2.6 Hz, 2H), 4.20 (t, J = 7.4 Hz, 2H), 4.05 (t, J = 6.6 Hz, 4H), 1.91 – 1.77 (m, 4H), 1.72 (p, J = 7.6 Hz, 2H), 1.56 – 1.47 (m, 4H), 1.43 – 1.33 (m, 8H), 1.32 – 1.24 (m, 2H), 1.23 – 1.17 (m, 4H), 0.97 – 0.89 (t, J = 6.9 Hz, 6H), 0.80 (t, J = 6.8 Hz, 3H).

^{13}C NMR (126 MHz, CD_2Cl_2): δ = 164.2, 156.8, 151.3, 148.8, 139.8, 138.0, 136.5, 116.6, 114.7, 113.1, 111.3, 68.8, 56.1, 32.3, 31.7, 30.1, 29.1, 26.6, 26.4, 23.3, 23.0, 14.4, 14.2.

^{195}Pt NMR (107 MHz, CD_2Cl_2): δ = -3549.

EM-MS (ESI+, MeOH, m/z) calcd. for $[M + H]^+$, 801.37052; found, 801.37181

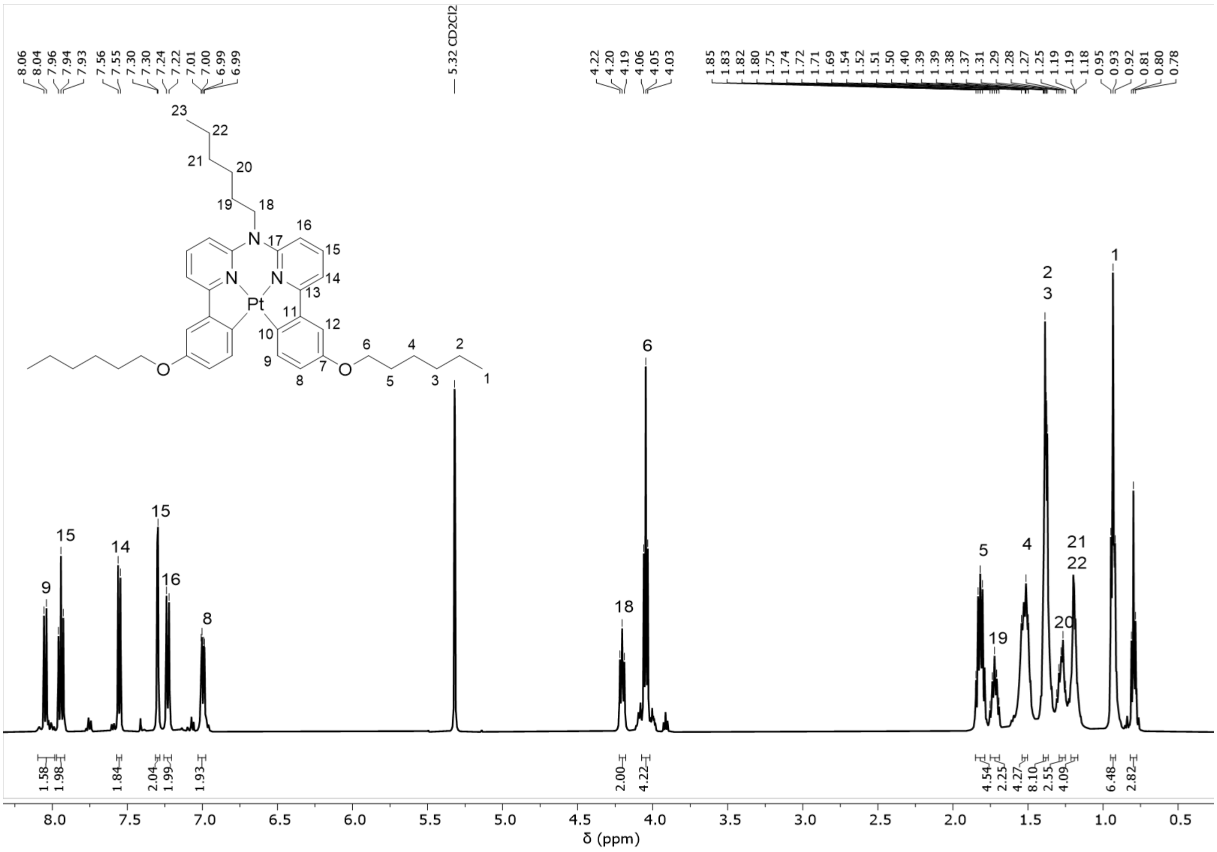

Figure S48. ^{1}H-NMR spectrum (500 MHz, CD_2Cl_2) of **PtL^{b}-6**.

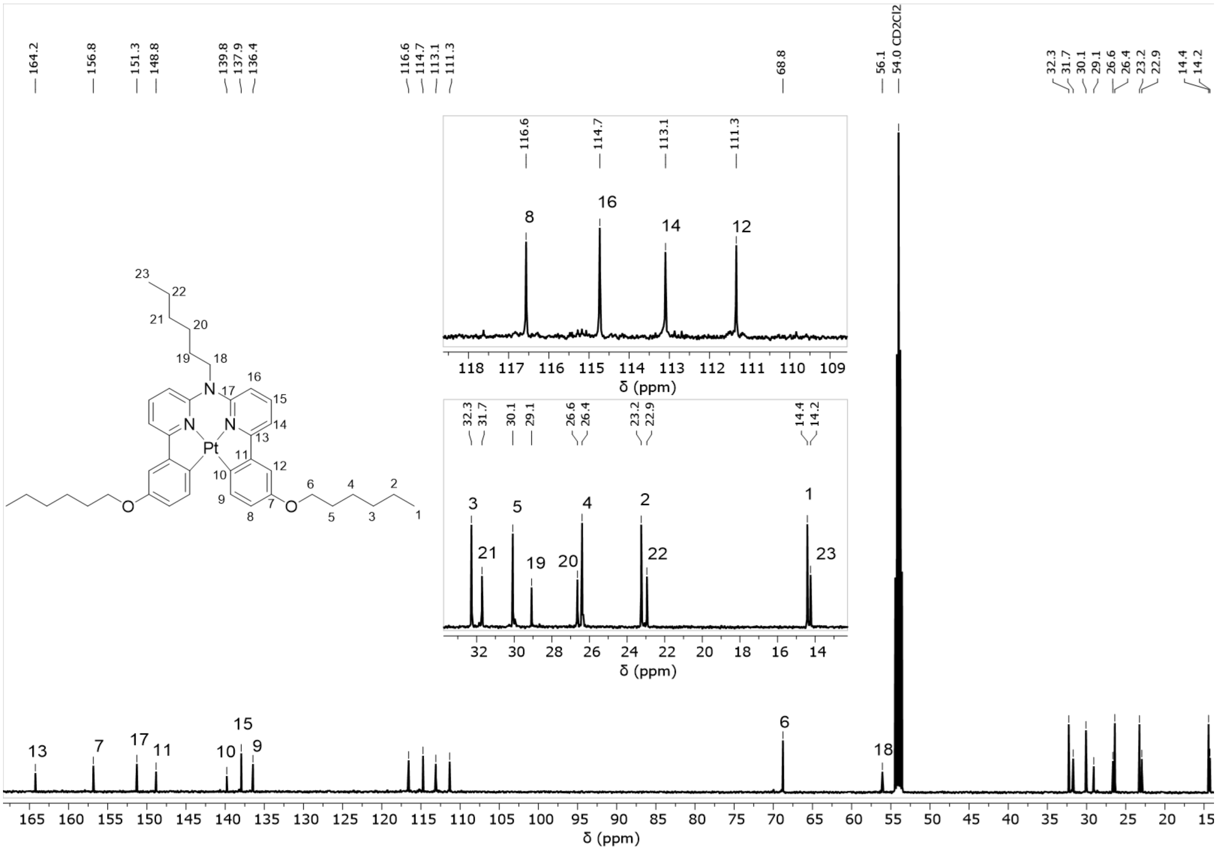

Figure S49. ^{13}C-{^{1}H}-NMR spectrum (126 MHz, CD_2Cl_2) of **PtL^{b}-6**.

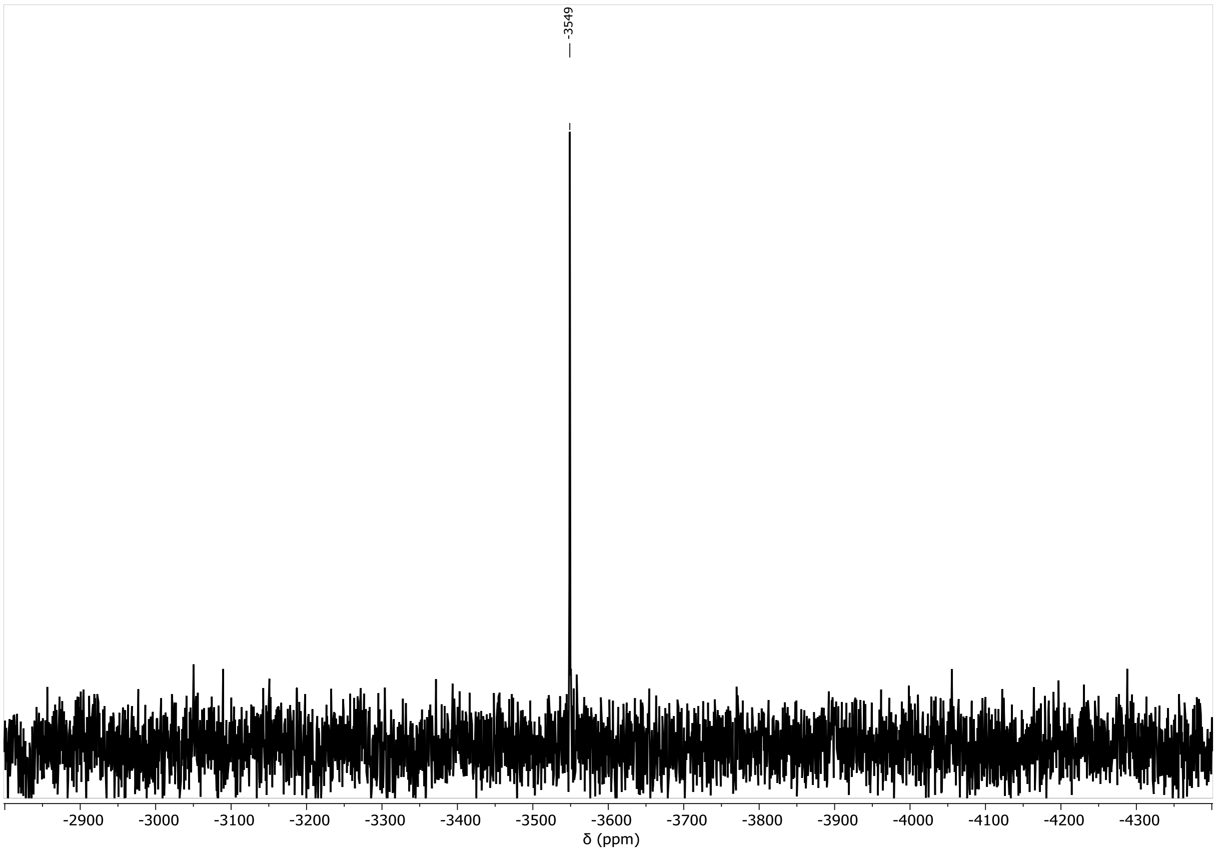

Figure S50. ^{195}Pt-NMR spectrum (107 MHz, CD_2Cl_2) of **PtL^b-6**.

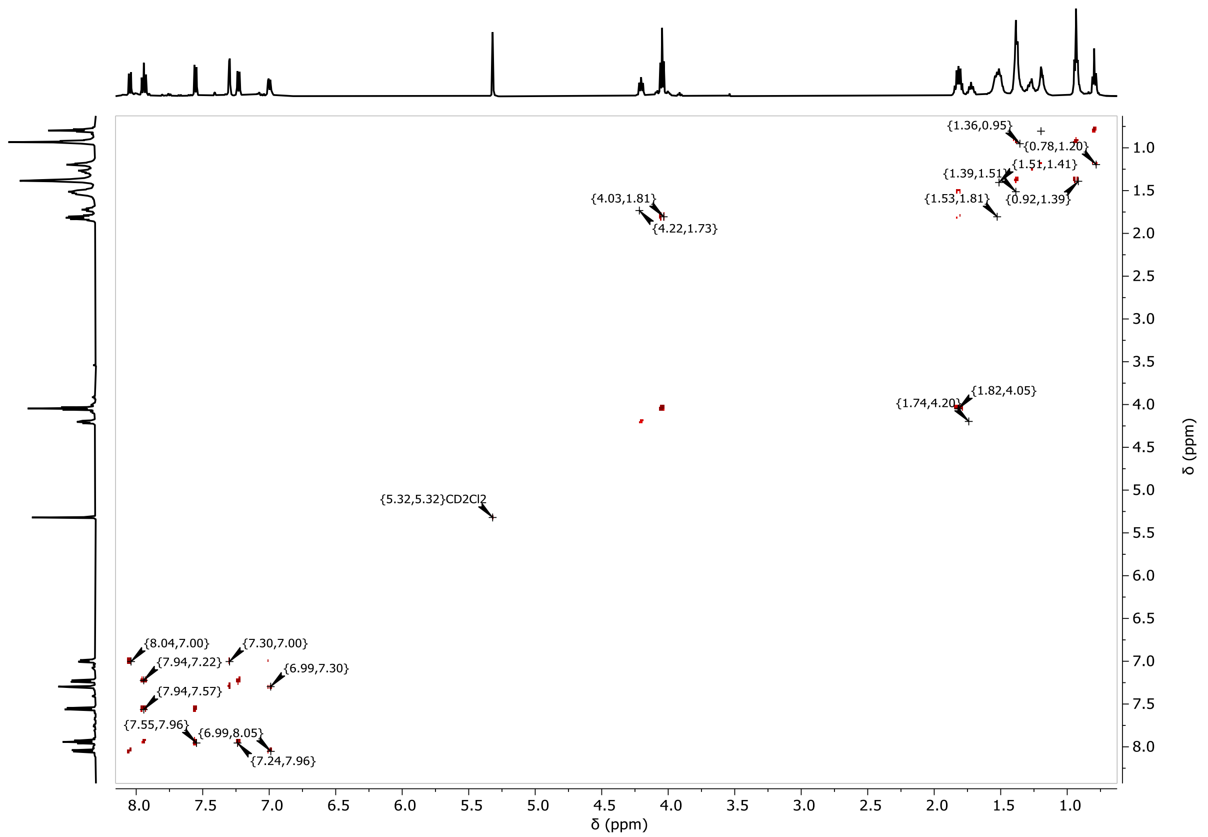

Figure S51. ^{1}H/^{1}H-COSY-NMR spectrum (500 MHz/500 MHz, CD_2Cl_2) of **PtL^b-6.**

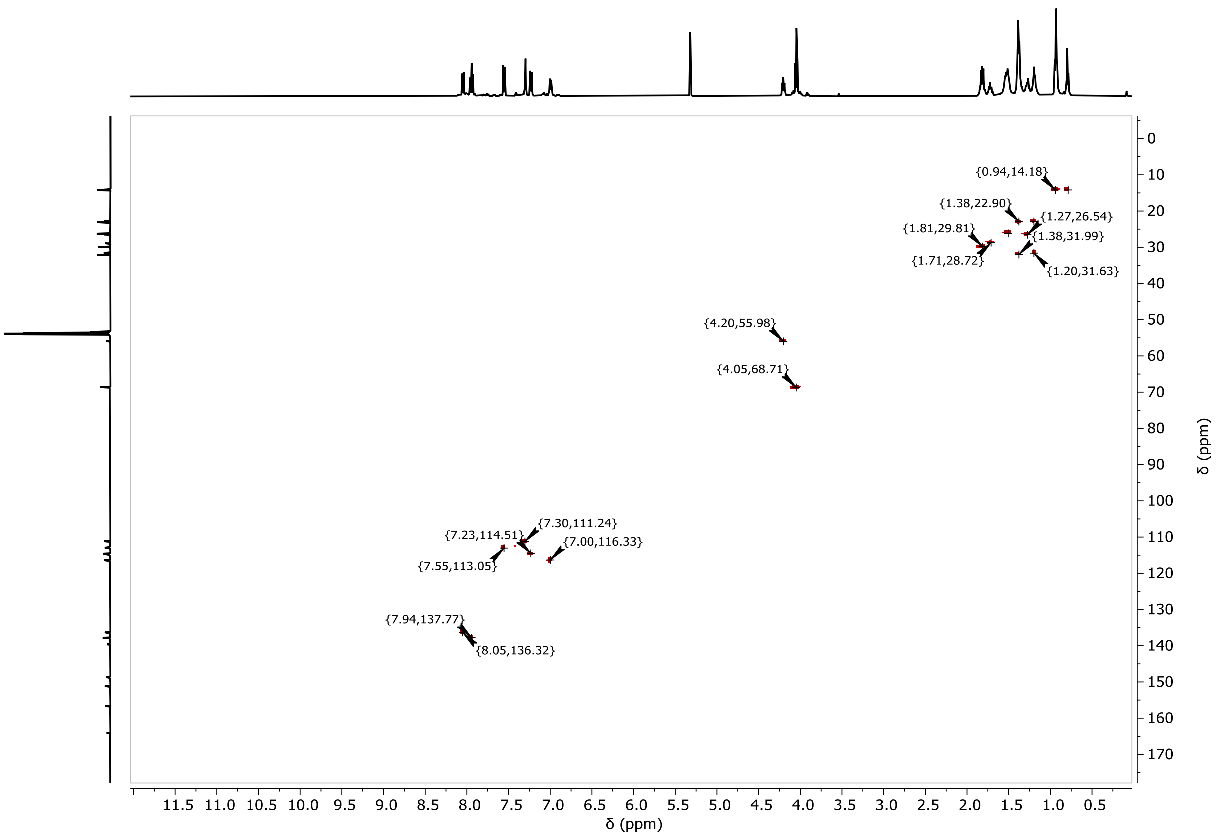

Figure S52. $^{1}H/^{13}C$-gHSQC-NMR spectrum (500 MHz/126 MHZ, CD_2Cl_2) of **PtL^{b}-6**.

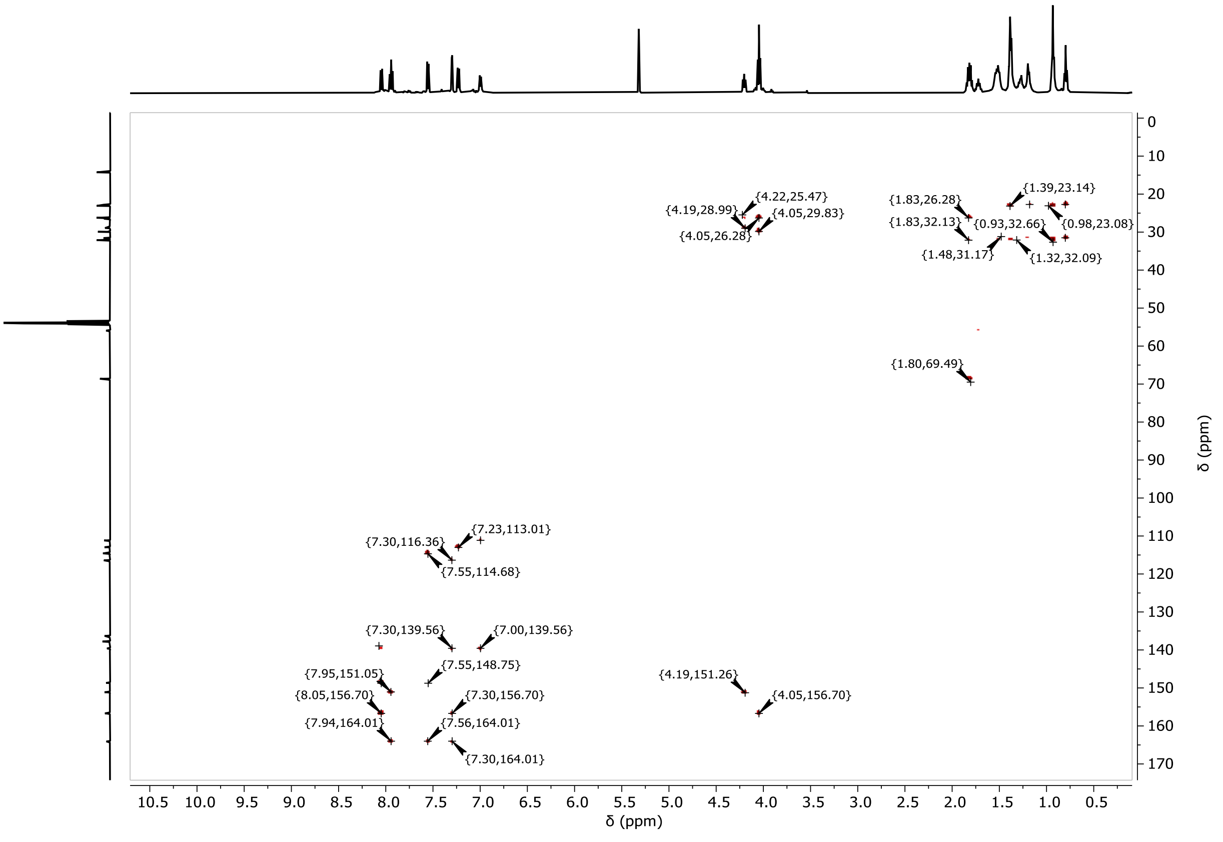

Figure S53. $^{1}H/^{13}C$-gHMBC-NMR spectrum (500 MHz/126 MHz, CD_2Cl_2) of **PtL^{b}-6**.

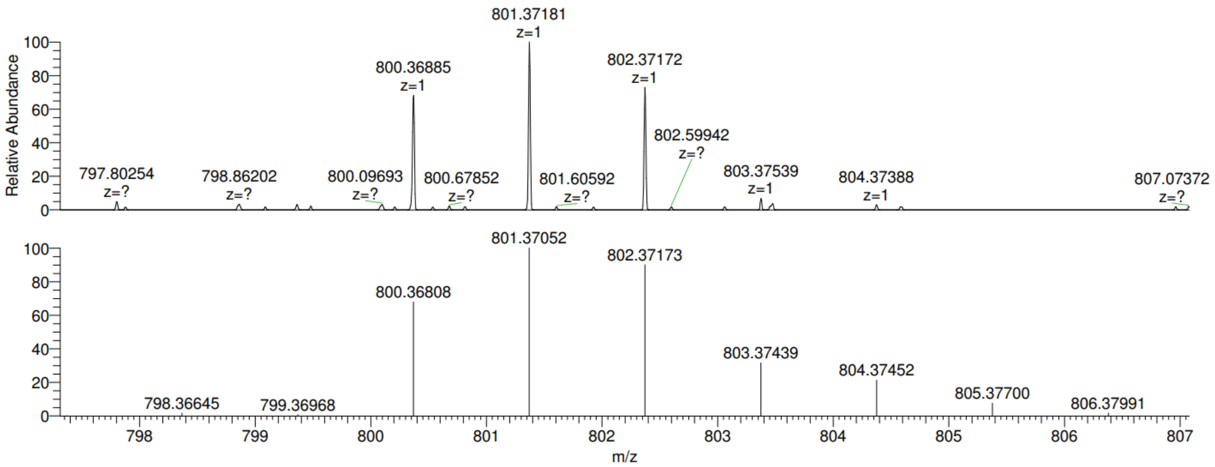

Figure S54. ESI-MS (MeOH) of **PtL^b-6**.

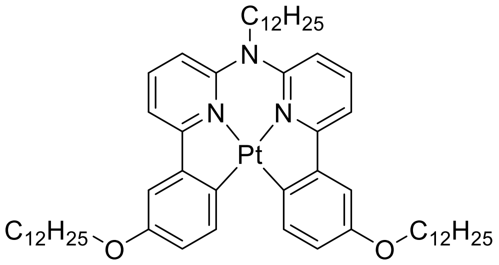

(N-dodecyl-N,N-bis(6-(3-(dodeciloxy)phenylido)pyridine-2-yl)-amine)-$\kappa^4_{C,N,N,C}$ platinum(II) (PtL^b-12)

In a 50 mL two-necked flask, **L^b-12** (192 mg, 0.22 mmol) and K_2PtCl_4 (93 mg, 0.22 mmol) were suspended using 15 mL of glacial acetic acid. The mixture was purged by bubbling with Ar at room temperature for 15 min and then heated up to 130 °C under Ar flux. The mixture was reacted for 72 h. The mixture was cooled down to room temperature and the solvent removed under reduced pressure. The crude product was adsorbed onto silica gel and purified by column chromatography using cyclohexane/CH_2Cl_2 as eluent (3:1 to 1:1) to yield a yellow plastic solid (103 mg, 44 %).

^{1}H NMR (500 MHz, CD_2Cl_2): δ = 8.06 (d, J = 8.4 Hz, 2H), 7.85 (t, J = 8.0 Hz, 2H), 7.49 (d, J = 7.7 Hz, 2H), 7.28 (d, J = 2.7 Hz, 2H), 7.13 (d, J = 8.4 Hz, 2H), 7.00 (dd, J = 8.3, 2.7 Hz, 2H), 4.10 (t, J = 7.3 Hz, 2H), 4.05 (t, J = 6.6 Hz, 4H), 1.88 – 1.78 (m, 4H), 1.67 (m, 2H), 1.70 – 1.64 (m, 6H), 1.44 – 1.13 (m, 54H), 0.89 (dt, J = 8.7, 6.9 Hz, 9H).

^{13}C NMR (126 MHz, CD_2Cl_2): δ = 164.1, 156.8, 151.1, 148.9, 140.0, 137.8, 136.5, 116.6, 114.6, 113.0, 111.3, 68.8, 56.0, 32.56, 30.3, 30.2, 30.1, 30.1, 30.0, 30.0, 29.5, 29.0, 26.9, 26.8, 23.3, 14.5.

^{195}Pt NMR (107 MHz, CD_2Cl_2): δ = -3548.

EM-MS (ESI+, MeOH, m/z): calcd. for $[M + H]^+$ = 1053.65234, found 1053.65359.

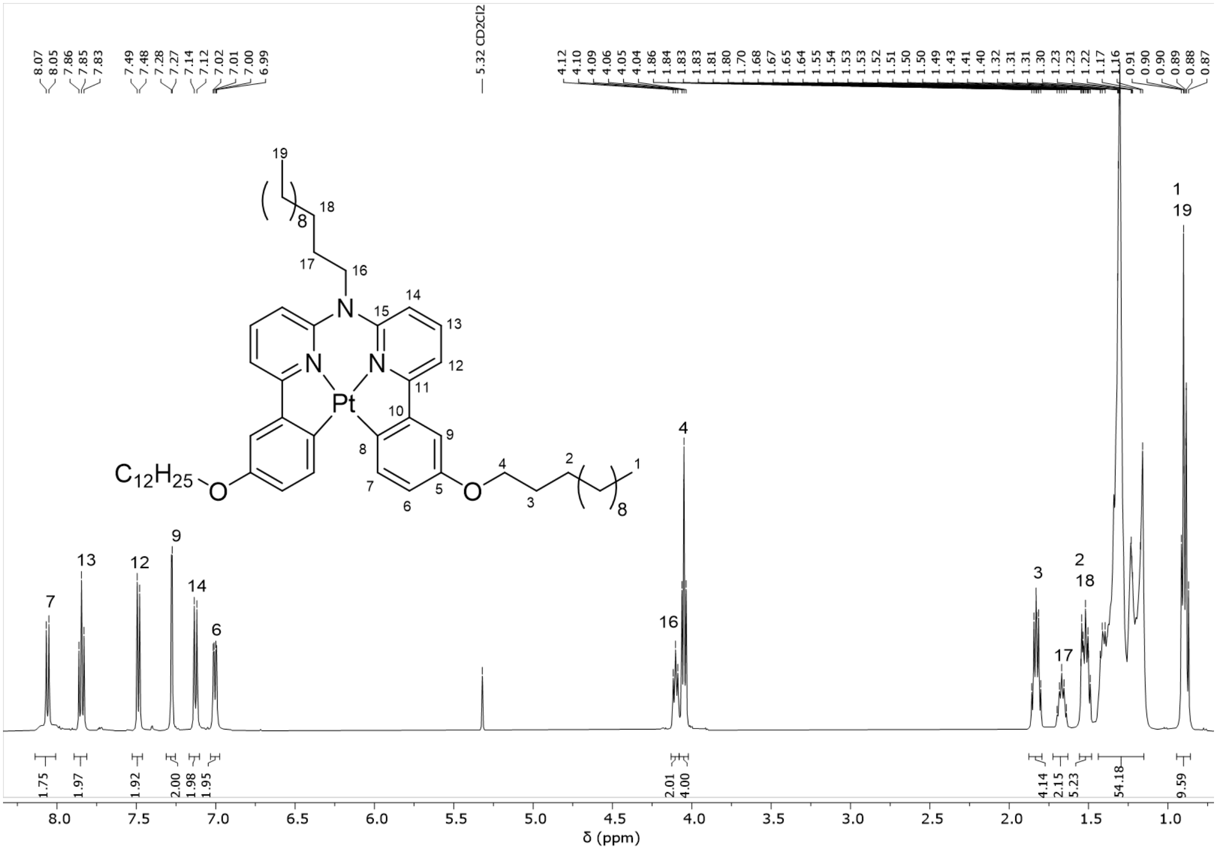

Figure S55. ^{1}H-NMR spectrum (500 MHz, CD_2Cl_2) of **PtL^b-12**.

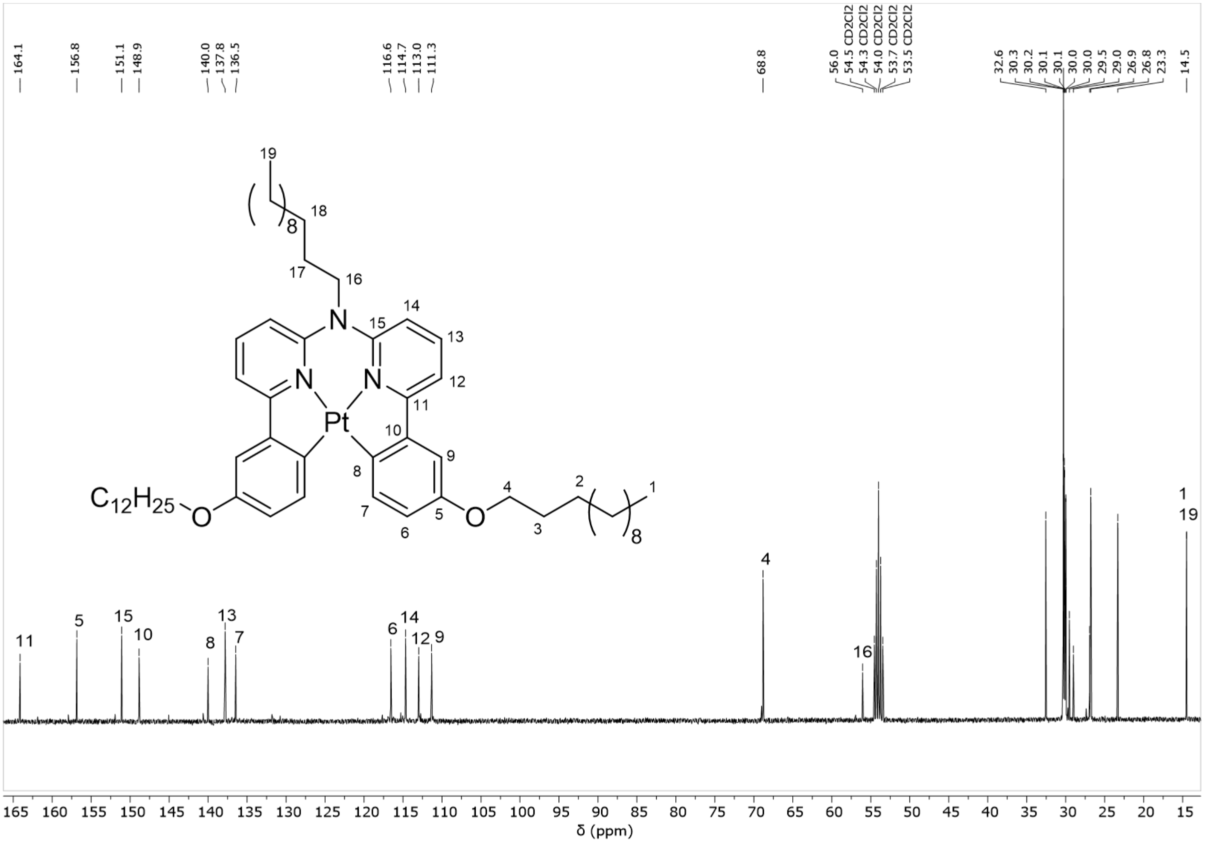

Figure S56. ^{13}C-{^{1}H}-NMR spectrum (126 MHz, CD_2Cl_2) of **PtL^b-12**.

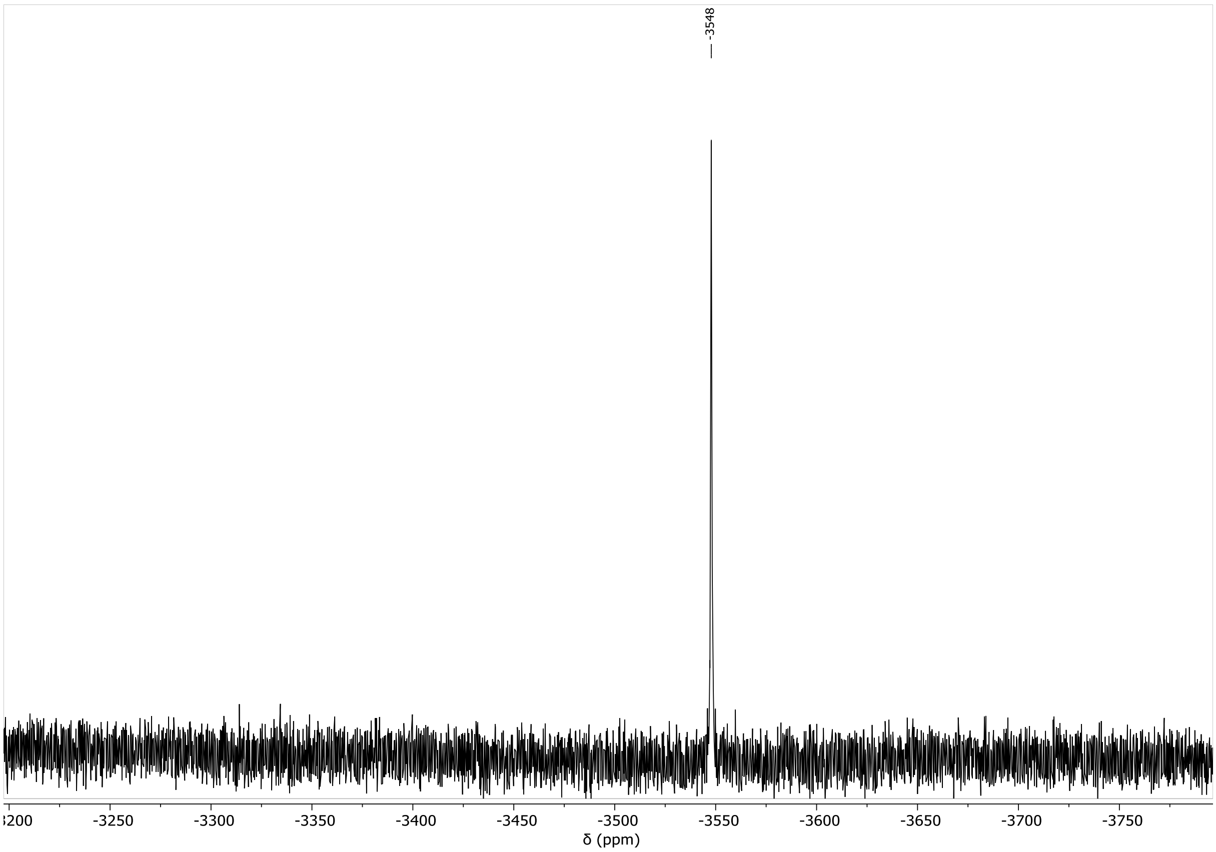

Figure S57. ^{195}Pt-NMR spectrum (107 MHz, CD_2Cl_2) of **PtL[a]-12**.

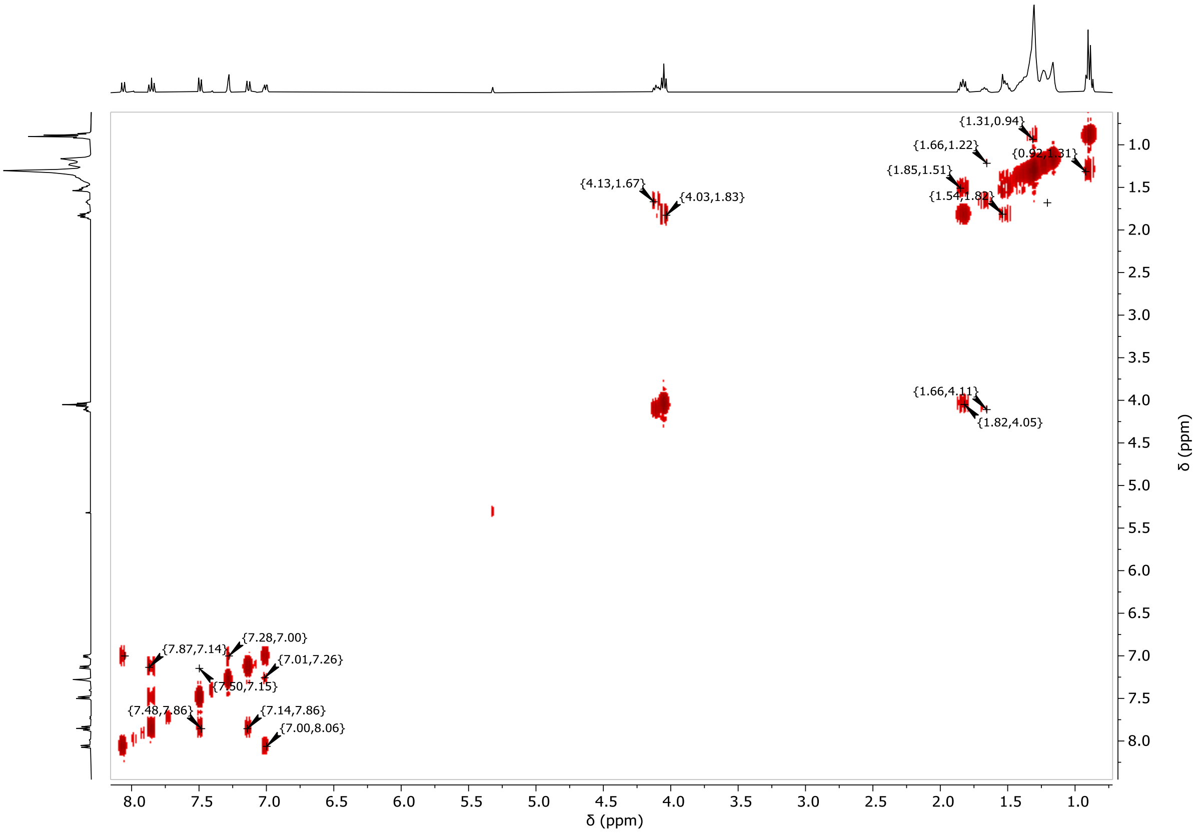

Figure S58. ^{1}H/^{1}H-COSY-NMR spectrum (500 MHz/500 MHz, CD_2Cl_2) of **PtL[b]-12.**

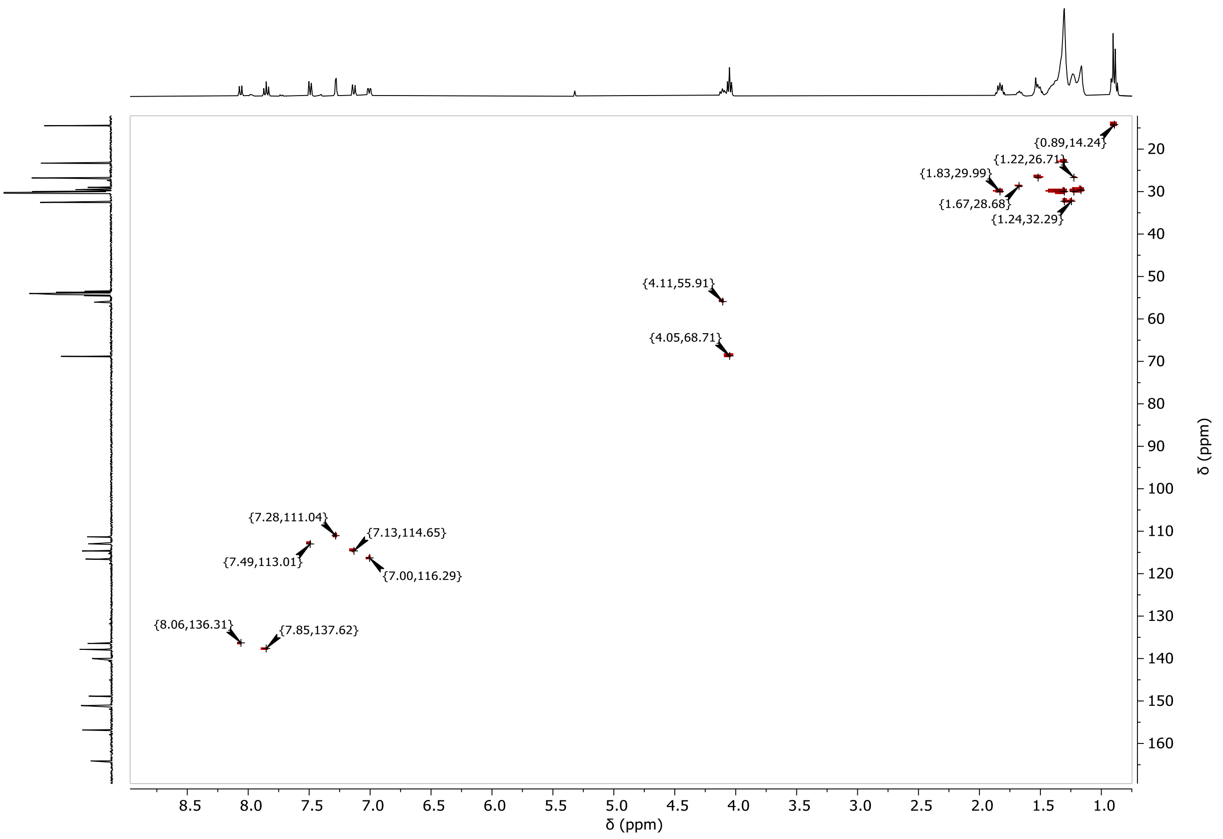

Figure S59. $^{1}H/^{13}C$-gHSQC-NMR spectrum (500 MHz/126 MHZ, CD_2Cl_2) of **PtL^{b}-12**.

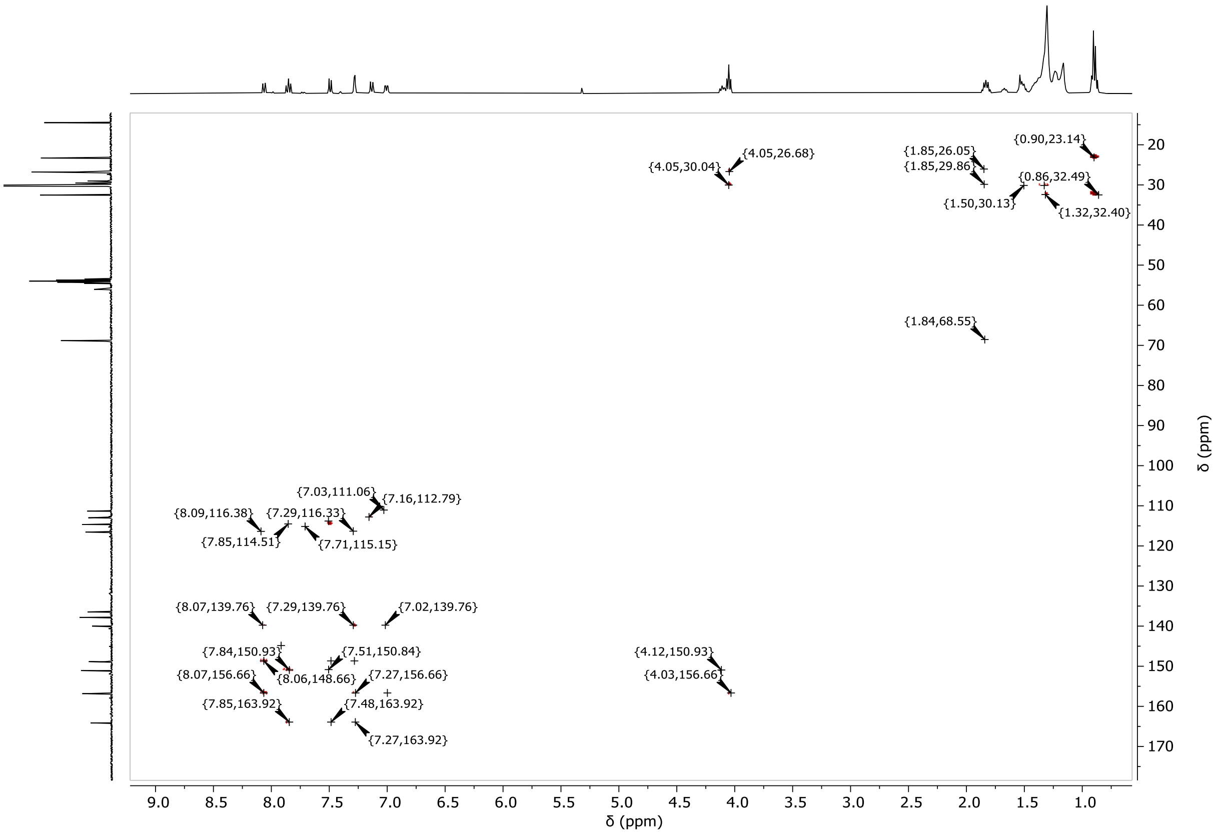

Figure S60. $^{1}H/^{13}C$-gHMBC-NMR spectrum (500 MHz/126 MHz, CD_2Cl_2) of **PtL^{b}-12**.

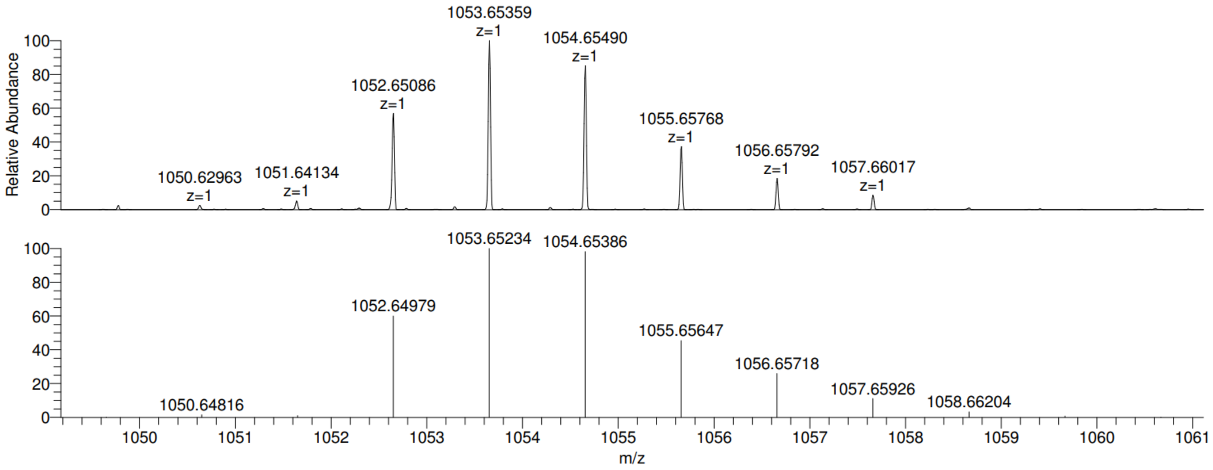

Figure S61. ESI-MS (MeOH) of **PtL^b-12**.

2) Photophysical characterization

All solvents used were of spectrometric grade (Merck Uvasol®).

Absorption spectra were measured with a Shimadzu UV-3600 I plus UV-VIS-NIR spectrophotometer. For all measurements, matched quartz (Hellma®) cuvettes were used.

Steady-state excitation and emission spectra were recorded on a FluoTime 300 spectrometer from PicoQuant equipped with a 300 W ozone-free Xe lamp (250-900 nm), a 10 W Xe flash-lamp (250-900 nm, pulse width < 10 µs) with repetition rates of 0.1 – 300 Hz, an excitation monochromator (Czerny-Turner 2.7 nm/mm dispersion, 1200 grooves/mm, blazed at 300 nm), diode lasers (pulse width < 80 ps) operated by a computer-controlled laser driver PDL-820 (repetition rate up to 80 MHz, burst mode for slow and weak decays), two emission monochromators (Czerny-Turner, selectable gratings blazed at 500 nm with 2.7 nm/mm dispersion and 1200 grooves/mm, or blazed at 1250 nm with 5.4 nm/mm dispersion and 600 grooves/mm), Glan-Thompson polarizers for excitation (Xe-lamps) and emission, a Peltier-thermostatized sample holder from Quantum Northwest (-40 °C – 105 °C), and two detectors, namely a PMA Hybrid 40 (transit time spread FWHM < 120 ps, 300 – 720 nm) and a R5509-42 NIR-photomultiplier tube (transit time spread FWHM 1.5 ns, 300- 1400 nm) with external cooling (-80 °C) from Hamamatsu. Steady-state spectra and fluorescence lifetimes were recorded in the TCSPC mode by a PicoHarp 300 (time-correlated single photon counting, minimum base resolution 4 ps). Phosphorescence lifetimes were recorded by a NanoHarp 250 (minimum base resolution 32 ns) in the multi-channel scaling (MCS) mode. Emission and excitation spectra were corrected for source intensity (lamp and grating) by standard correction curves. The lifetime analysis was performed using the commercial FluoFit software. The quality of the fit was assessed by minimizing the reduced chi squared function (χ2) and visual inspection of the weighted residuals and their autocorrelation. The values obtained from the analysis were rounded to the first significative figure.

Luminescence quantum yields were measured with a Hamamatsu Photonics absolute PL quantum yield measurement system (C9920-02) equipped with a L9799-01 CW xenon light source (150 W), monochromator, C7473 photonic multi-channel analyzer, integrating sphere and employing U6039-05 PLQY measurement software (Hamamatsu Photonics, Ltd., Shizuoka, Japan).

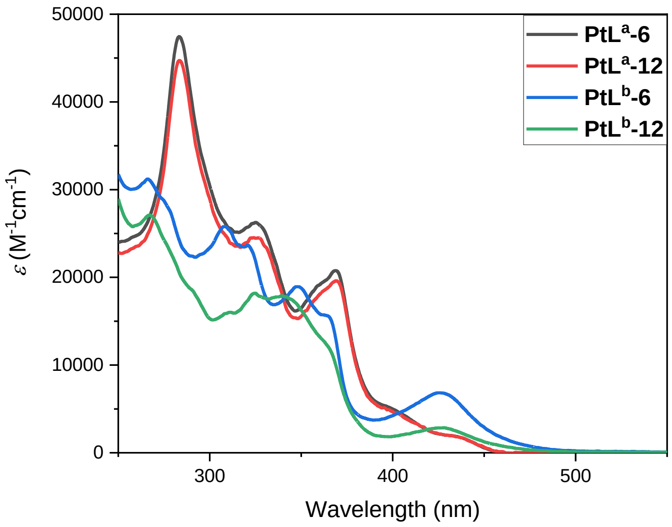

Figure S62. Absorption spectra of the four complexes in DCM at room temperature.

Table S1. Absorption coefficients of the four complexes at selected wavelengths.

Complex	λ (nm) / ε ($M^{-1}cm^{-1}$)
**PtL^a-6**	283 / (47 ± 1) x 10^3; 325 / (26.3 ± 0.7) x 10^3; 369 / (20.8 ± 0.5) x 10^3
**PtL^a-12**	283 / (44.8 ± 0.4) x 10^3; 322 / (24.6 ± 0.5) x 10^3; 366 / (19.7 ± 0.2) x 10^3
**PtL^b-6**	266 / (31.3 ± 0.1) x 10^3; 308 / (25.80 ± 0.06) x 10^3; 347 / (18.95 ± 0.05) x 10^3; 426 / (6.82 ± 0.02) x 10^3
**PtL^b-12**	267 / (27.1 ± 0.2) x 10^3; 339 / (17.9 ± 0.1) x 10^3; 428 / (2.88 ± 0.04) x 10^3

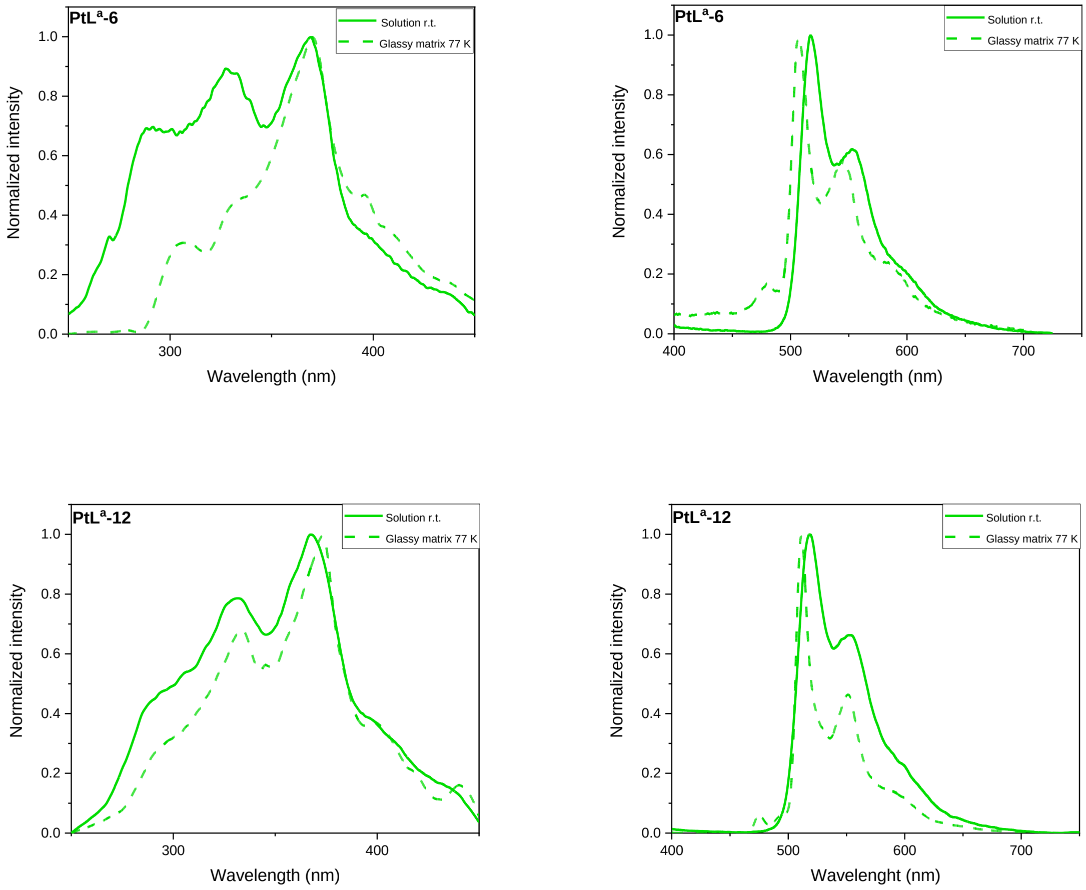

Figure S63. Excitation (left, at the corresponding emission maximum) and emission (right, λ_{exc} = 350 nm) spectra of **PtL^a-6** and **PtL^a-12**.

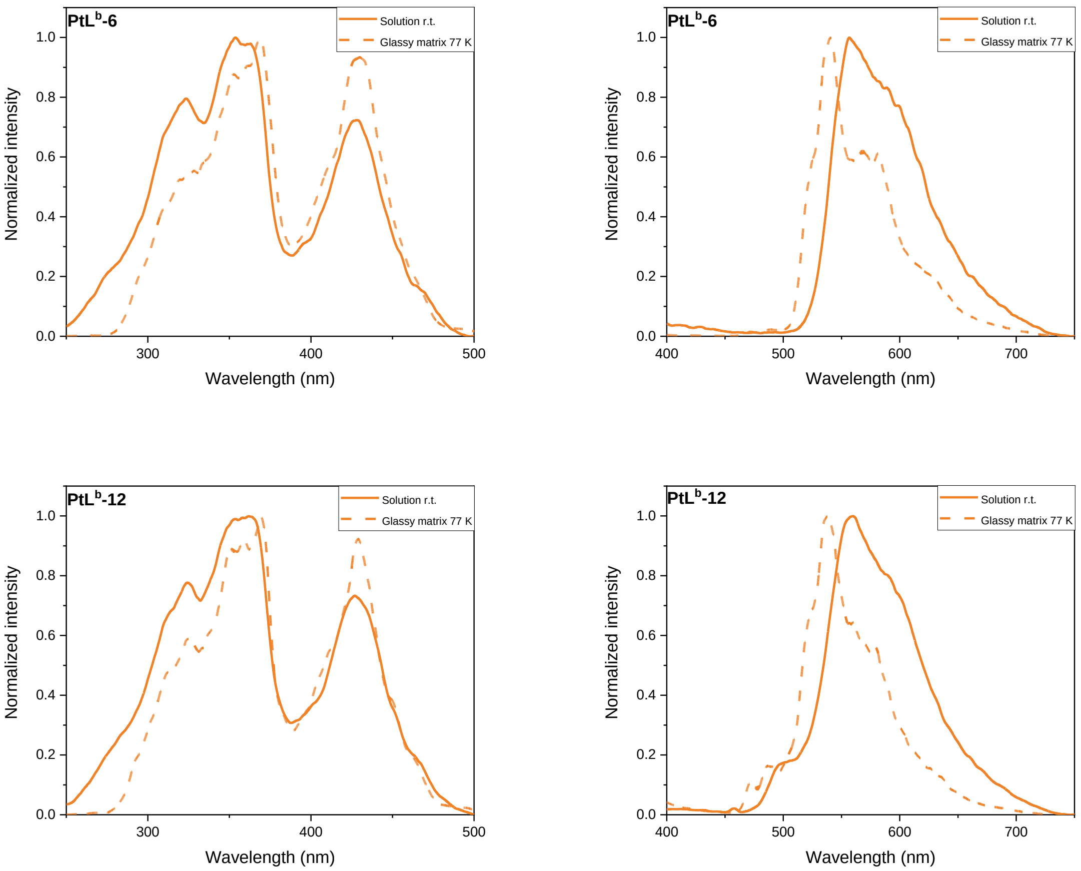

Figure S64. Excitation (left, at the corresponding emission maximum) and emission (right, λ_{exc} = 350 nm) spectra of **PtL^b-6** and **PtL^b-12**.

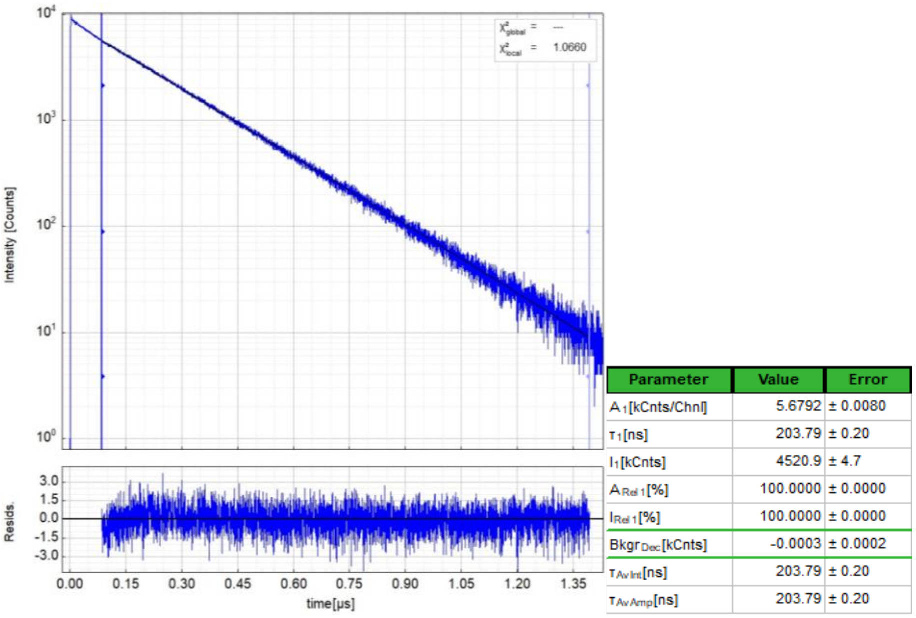

Parameter	Value	Error
A_1[kCnts/Chnl]	5.6792	± 0.0080
τ_1[ns]	203.79	± 0.20
I_1[kCnts]	4520.9	± 4.7
$A_{Rel\ 1}$[%]	100.0000	± 0.0000
$I_{Rel\ 1}$[%]	100.0000	± 0.0000
$Bkgr_{Dec}$[kCnts]	-0.0003	± 0.0002
$\tau_{Av\ Int}$[ns]	203.79	± 0.20
$\tau_{Av\ Amp}$[ns]	203.79	± 0.20

Figure S65. Left: Raw time-resolved photoluminescence decay of **PtL^a-6** ($c \approx 10^{-5}$ M) in fluid air-equilibrated DCM at r.t., including the residuals (λ_{exc} = 376.7 nm, λ_{em} = 515 nm). Right: Fitting parameters including pre-exponential factors and confidence limits.

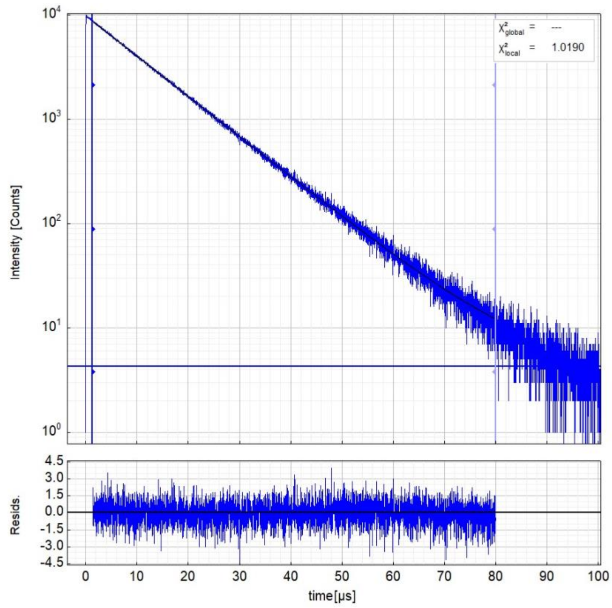

Parameter	Value	Error
A_1[kCnts/Chnl]	8.6924	± 0.0078
τ_1[ns]	11226	± 16
I_1[kCnts]	6098.9	± 4.4
$A_{Rel\,1}$[%]	100.0000	± 0.0000
$I_{Rel\,1}$[%]	100.0000	± 0.0000
$Bkgr_{Dec}$[kCnts]	0.0043	± 0.0003
$\tau_{Av\,Int}$[ns]	11226	± 16
$\tau_{Av\,Amp}$[ns]	11226	± 16

Figure S66. Left: Raw time-resolved photoluminescence decay of **PtL^a-6** (*c* ≈ 10^{-5} M) in fluid Ar-purged DCM at r.t., including the residuals (λ_{exc} = 376.7 nm, λ_{em} = 515 nm). Right: Fitting parameters including pre-exponential factors and confidence limits.

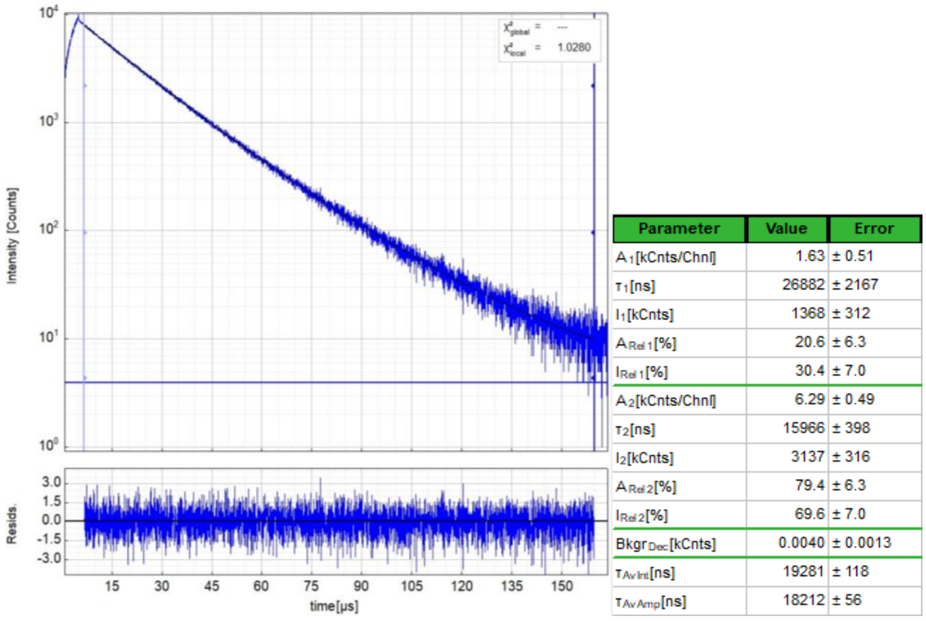

Parameter	Value	Error
A_1[kCnts/Chnl]	1.63	± 0.51
τ_1[ns]	26882	± 2167
I_1[kCnts]	1368	± 312
$A_{Rel\,1}$[%]	20.6	± 6.3
$I_{Rel\,1}$[%]	30.4	± 7.0
A_2[kCnts/Chnl]	6.29	± 0.49
τ_2[ns]	15966	± 398
I_2[kCnts]	3137	± 316
$A_{Rel\,2}$[%]	79.4	± 6.3
$I_{Rel\,2}$[%]	69.6	± 7.0
$Bkgr_{Dec}$[kCnts]	0.0040	± 0.0013
$\tau_{Av\,Int}$[ns]	19281	± 118
$\tau_{Av\,Amp}$[ns]	18212	± 56

Figure S67. Left: Raw time-resolved photoluminescence decay of **PtL^a-6** (c ≈ 10^{-5} M) in a frozen glassy matrix of 2Me-THF at 77 K, including the residuals (λ_{exc} = 376.7 nm, λ_{em} = 505 nm). Right: Fitting parameters including pre-exponential factors and confidence limits.

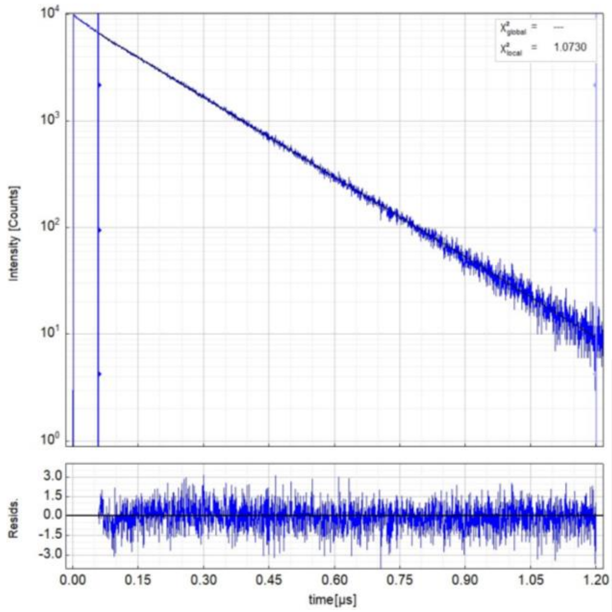

Parameter	Value	Error
A_1[kCnts/Chnl]	6.628	± 0.011
τ_1[ns]	174.45	± 0.28
I_1[kCnts]	2258.2	± 3.6
$A_{Rel\,1}$[%]	100.0000	± 0.0000
$I_{Rel\,1}$[%]	100.0000	± 0.0000
$Bkgr_{Dec}$[kCnts]	-0.0003	± 0.0004
$\tau_{Av\,Int}$[ns]	174.45	± 0.28
$\tau_{Av\,Amp}$[ns]	174.45	± 0.28

Figure S68. Left: Raw time-resolved photoluminescence decay of **PtL^a-12** (c ≈ 10^{-5} M) in fluid air-equilibrated DCM at r.t., including the residuals (λ_{exc} = 376.7 nm, λ_{em} = 515 nm). Right: Fitting parameters including pre-exponential factors and confidence limits.

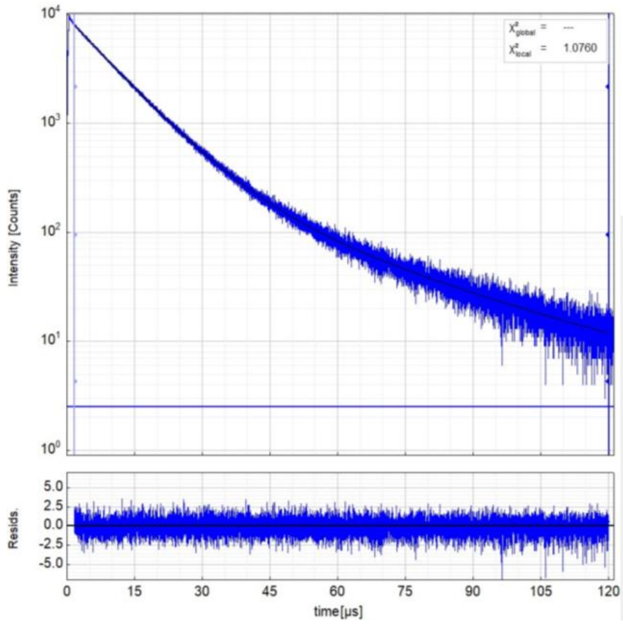

Parameter	Value	Error
A_1[kCnts/Chnl]	0.422	± 0.024
τ_1[ns]	31129	± 943
I_1[kCnts]	1640	± 41
$A_{Rel\,1}$[%]	5.26	± 0.30
$I_{Rel\,1}$[%]	15.47	± 0.41
A_2[kCnts/Chnl]	7.585	± 0.019
τ_2[ns]	9455	± 34
I_2[kCnts]	8964	± 54
$A_{Rel\,2}$[%]	94.74	± 0.30
$I_{Rel\,2}$[%]	84.53	± 0.41
$Bkgr_{Dec}$[kCnts]	0.0025	± 0.0010
$\tau_{Av\,Int}$[ns]	12807	± 111
$\tau_{Av\,Amp}$[ns]	10596	± 20

Figure S69. Left: Raw time-resolved photoluminescence decay of **PtL^a-12** (c ≈ 10^{-5} M) in fluid Ar-purged DCM at r.t., including the residuals (λ_{exc} = 376.7 nm, λ_{em} = 515 nm). Right: Fitting parameters including pre-exponential factors and confidence limits.

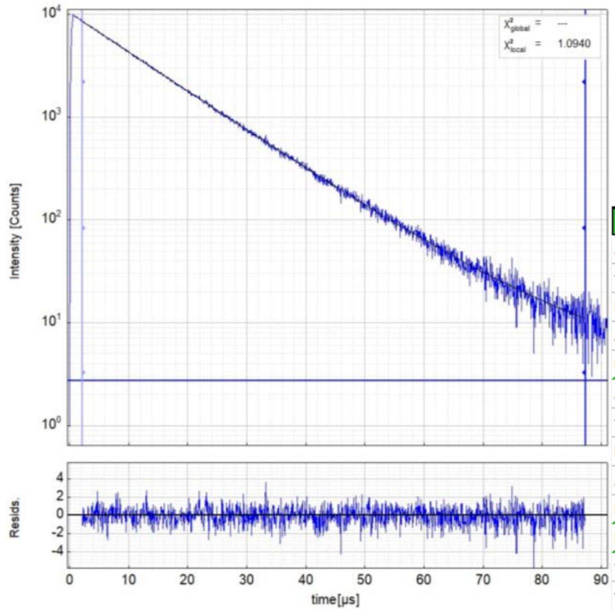

Parameter	Value	Error
A_1[kCnts/Chnl]	0.2	± 5.2
τ_1[ns]	22806	± 124503
I_1[kCnts]	61	± 974
$A_{Rel\,1}$[%]	2	± 61
$I_{Rel\,1}$[%]	4	± 65
A_2[kCnts/Chnl]	8.4	± 5.2
τ_2[ns]	11199	± 1461
I_2[kCnts]	1469	± 982
$A_{Rel\,2}$[%]	98	± 61
$I_{Rel\,2}$[%]	96	± 65
$Bkgr_{Dec}$[kCnts]	0.003	± 0.031
$\tau_{Av\,Int}$[ns]	11659	± 11034
$\tau_{Av\,Amp}$[ns]	11429	± 837

Figure S70. Left: Raw time-resolved photoluminescence decay of **PtL^a-12** (c ≈ 10^{-5} M) in a frozen glassy matrix of 2Me-THF at 77 K, including the residuals (λ_{exc} = 376.7 nm, λ_{em} = 505 nm). Right: Fitting parameters including pre-exponential factors and confidence limits.

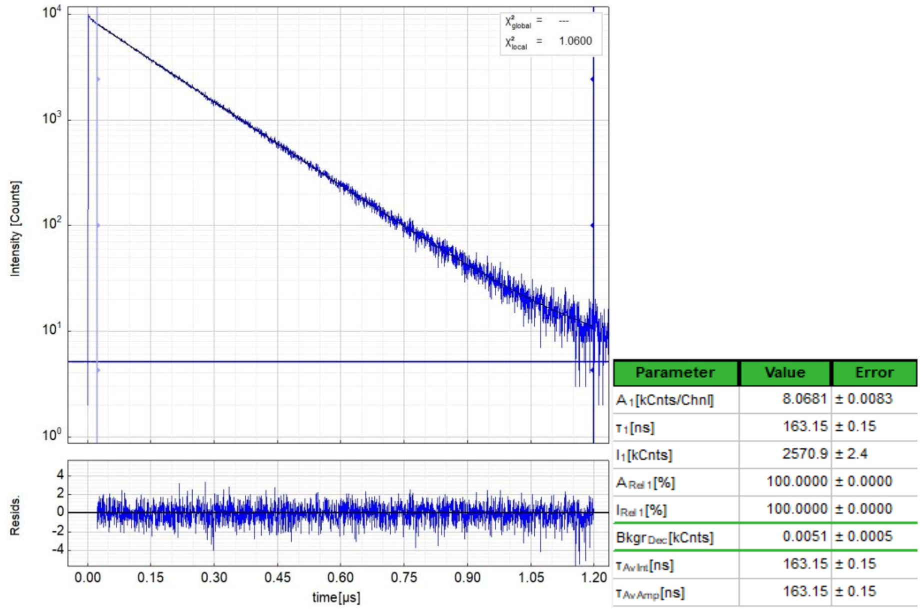

Parameter	Value	Error
A_1[kCnts/Chnl]	8.0681	± 0.0083
τ_1[ns]	163.15	± 0.15
I_1[kCnts]	2570.9	± 2.4
$A_{Rel\,1}$[%]	100.0000	± 0.0000
$I_{Rel\,1}$[%]	100.0000	± 0.0000
$Bkgr_{Dec}$[kCnts]	0.0051	± 0.0005
$\tau_{Av\,Int}$[ns]	163.15	± 0.15
$\tau_{Av\,Amp}$[ns]	163.15	± 0.15

Figure S71. Left: Raw time-resolved photoluminescence decay of **PtL^b-6** (c ≈ 10^{-5} M) in fluid air-equilibrated DCM at r.t., including the residuals (λ_{exc} = 376.7 nm, λ_{em} = 555 nm). Right: Fitting parameters including pre-exponential factors and confidence limits.

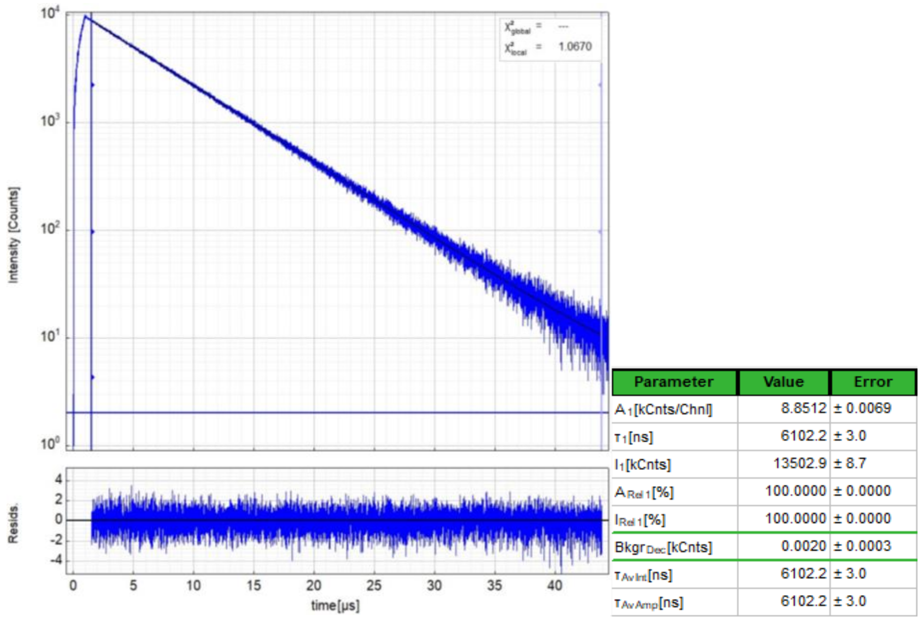

Parameter	Value	Error
A_1[kCnts/Chnl]	8.8512	± 0.0069
τ_1[ns]	6102.2	± 3.0
I_1[kCnts]	13502.9	± 8.7
$A_{Rel\,1}$[%]	100.0000	± 0.0000
$I_{Rel\,1}$[%]	100.0000	± 0.0000
$Bkgr_{Dec}$[kCnts]	0.0020	± 0.0003
$\tau_{Av\,Int}$[ns]	6102.2	± 3.0
$\tau_{Av\,Amp}$[ns]	6102.2	± 3.0

Figure S72. Left: Raw time-resolved photoluminescence decay of **PtL^b-6** (c ≈ 10^{-5} M) in fluid Ar-purged DCM at r.t., including the residuals (λ_{exc} = 376.7 nm, λ_{em} = 555 nm). Right: Fitting parameters including pre-exponential factors and confidence limits.

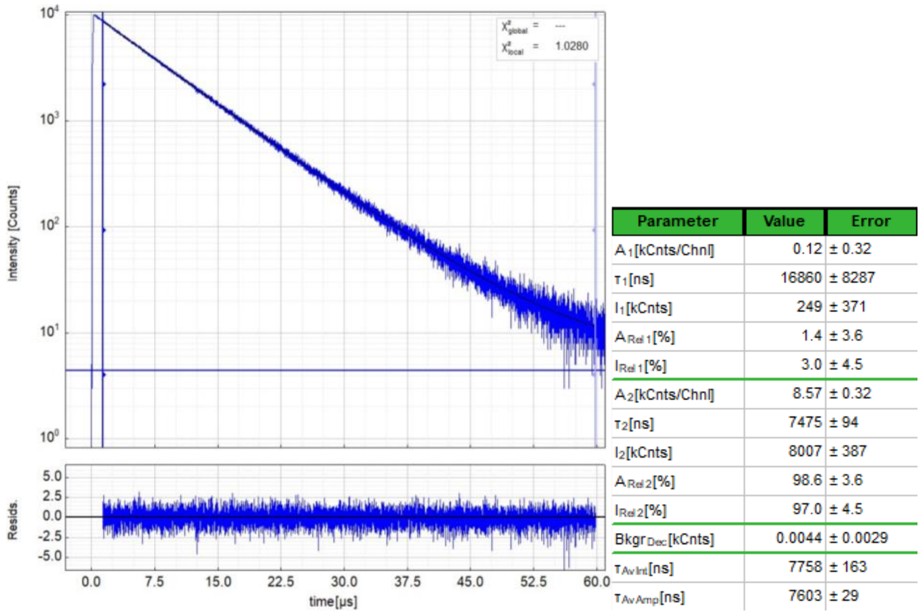

Parameter	Value	Error
A_1[kCnts/Chnl]	0.12	± 0.32
τ_1[ns]	16860	± 8287
I_1[kCnts]	249	± 371
$A_{Rel\,1}$[%]	1.4	± 3.6
$I_{Rel\,1}$[%]	3.0	± 4.5
A_2[kCnts/Chnl]	8.57	± 0.32
τ_2[ns]	7475	± 94
I_2[kCnts]	8007	± 387
$A_{Rel\,2}$[%]	98.6	± 3.6
$I_{Rel\,2}$[%]	97.0	± 4.5
$Bkgr_{Dec}$[kCnts]	0.0044	± 0.0029
$\tau_{Av\,Int}$[ns]	7758	± 163
$\tau_{Av\,Amp}$[ns]	7603	± 29

Figure S73. Left: Raw time-resolved photoluminescence decay of **PtL^b-6** (c ≈ 10^{-5} M) in a frozen glassy matrix of 2Me-THF at 77 K, including the residuals (λ_{exc} = 376.7 nm, λ_{em} = 540 nm). Right: Fitting parameters including pre-exponential factors and confidence limits.

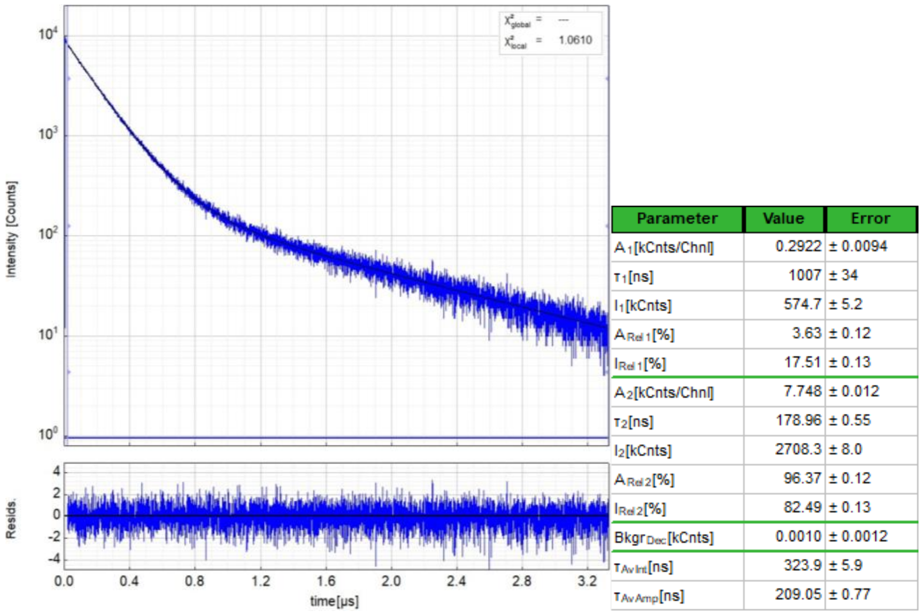

Parameter	Value	Error
A_1[kCnts/Chnl]	0.2922	± 0.0094
τ_1[ns]	1007	± 34
I_1[kCnts]	574.7	± 5.2
$A_{Rel\,1}$[%]	3.63	± 0.12
$I_{Rel\,1}$[%]	17.51	± 0.13
A_2[kCnts/Chnl]	7.748	± 0.012
τ_2[ns]	178.96	± 0.55
I_2[kCnts]	2708.3	± 8.0
$A_{Rel\,2}$[%]	96.37	± 0.12
$I_{Rel\,2}$[%]	82.49	± 0.13
$Bkgr_{Dec}$[kCnts]	0.0010	± 0.0012
$\tau_{Av\,Int}$[ns]	323.9	± 5.9
$\tau_{Av\,Amp}$[ns]	209.05	± 0.77

Figure S74. Left: Raw time-resolved photoluminescence decay of **PtL^b-12** (c ≈ 10^{-5} M) in fluid air-equilibrated DCM at r.t., including the residuals (λ_{exc} = 376.7 nm, λ_{em} = 555 nm). Right: Fitting parameters including pre-exponential factors and confidence limits.

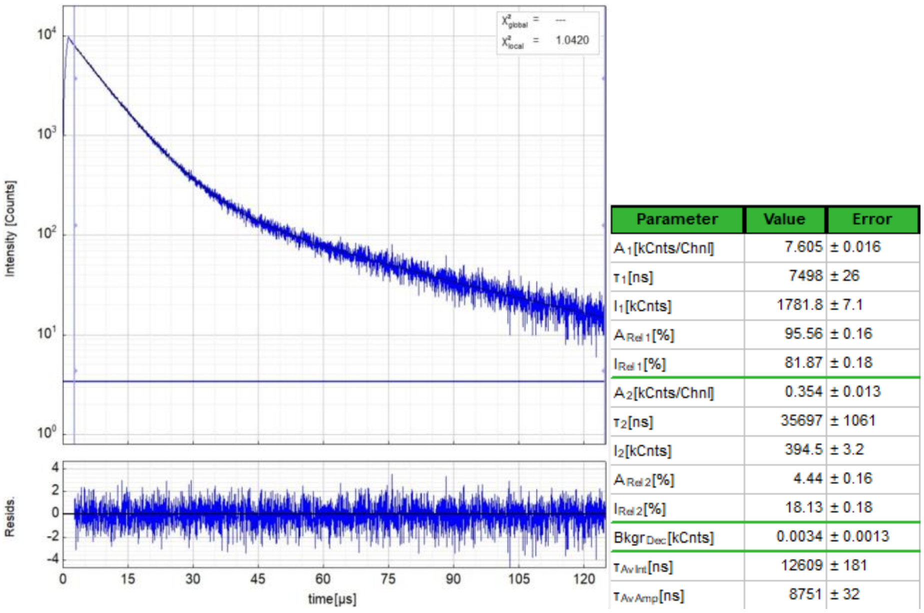

Parameter	Value	Error
A_1[kCnts/Chnl]	7.605	± 0.016
τ_1[ns]	7498	± 26
I_1[kCnts]	1781.8	± 7.1
$A_{Rel\,1}$[%]	95.56	± 0.16
$I_{Rel\,1}$[%]	81.87	± 0.18
A_2[kCnts/Chnl]	0.354	± 0.013
τ_2[ns]	35697	± 1061
I_2[kCnts]	394.5	± 3.2
$A_{Rel\,2}$[%]	4.44	± 0.16
$I_{Rel\,2}$[%]	18.13	± 0.18
$Bkgr_{Dec}$[kCnts]	0.0034	± 0.0013
$\tau_{Av\,Int}$[ns]	12609	± 181
$\tau_{Av\,Amp}$[ns]	8751	± 32

Figure S75. Left: Raw time-resolved photoluminescence decay of **PtL^b-12** (c ≈ 10^{-5} M) in fluid Ar-purged DCM at r.t., including the residuals (λ_{exc} = 376.7 nm, λ_{em} = 555 nm). Right: Fitting parameters including pre-exponential factors and confidence limits.

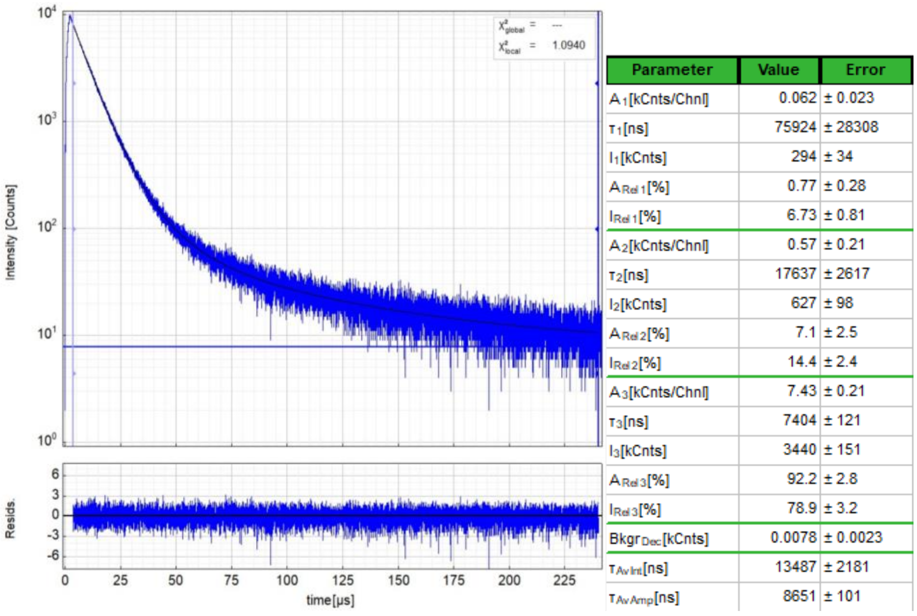

Parameter	Value	Error
A_1[kCnts/Chnl]	0.062	± 0.023
τ_1[ns]	75924	± 28308
I_1[kCnts]	294	± 34
$A_{Rel\,1}$[%]	0.77	± 0.28
$I_{Rel\,1}$[%]	6.73	± 0.81
A_2[kCnts/Chnl]	0.57	± 0.21
τ_2[ns]	17637	± 2617
I_2[kCnts]	627	± 98
$A_{Rel\,2}$[%]	7.1	± 2.5
$I_{Rel\,2}$[%]	14.4	± 2.4
A_3[kCnts/Chnl]	7.43	± 0.21
τ_3[ns]	7404	± 121
I_3[kCnts]	3440	± 151
$A_{Rel\,3}$[%]	92.2	± 2.8
$I_{Rel\,3}$[%]	78.9	± 3.2
$Bkgr_{Dec}$[kCnts]	0.0078	± 0.0023
$\tau_{Av\,Int}$[ns]	13487	± 2181
$\tau_{Av\,Amp}$[ns]	8651	± 101

Figure S76. Left: Raw time-resolved photoluminescence decay of **PtL^b-12** (c ≈ 10^{-5} M) in a frozen glassy matrix of 2Me-THF at 77 K, including the residuals (λ_{exc} = 376.7 nm, λ_{em} = 540 nm). Right: Fitting parameters including pre-exponential factors and confidence limits.

3) Theoretical calculations

All density functional theory (DFT) calculations were performed using the quantum chemistry package Gaussian 09 Rev. D.01,[1] with the PBE0 exchange-correlation functionals and the SDD basis set,[2] which applies an effective core potential for the Pt atoms and the D95 basis set for H, C, N, and O atoms.[3,4] Grimme's D3 dispersion correction was applied with Becke-Johnson damping.[5] The solvent tetrahydrofuran (THF) was described by the polarizable continuum model (PCM) in an integral equation formalism framework with atomic radii from the universal force field model (UFF).[6,7]

The T_1 geometry was energetically optimized using Kohn-Sham DFT with multiplicity 3. The character of the excited state was determined using time-dependent DFT (TDDFT) linear response theory and a subsequent correlated electron-hole pair analysis using the software TheoDORE.[8] Molecular orbitals were visualized using Avogadro 1.2.0.[9]

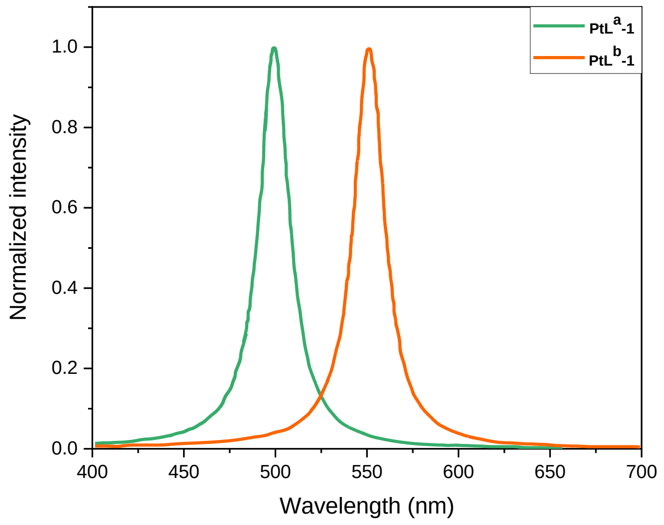

Figure S77. Calculated emission spectra at 77 K in THF.

4) Thermochromic properties

DSC traces were recorded with a Mettler Toledo DSC3+. Samples (2-5 mg) were heated with a rate of 10 K/min and Ar was used as flushing gas with a flow of 20 mL/min. Samples were measured three times.

Polarized optical microscopy (POM) images were acquired on a Nikon Eclipse Ni equipped with a Linkam heating station and an OPTIX-CAM SUMNIT 2K camera. A heating rate of 10 K/min was used.

Temperature-dependent luminescence of the solid samples were measured on a Shimadzu RF-6000 fluorospectrometer. The solid samples were placed between quartz glass slides and fixed with clips. The sample was placed in a homebuilt heating stage and installed inside the fluorospectrometer using an adaptable solid sample holder. The lit was closed and temperature was subsequently increased. Each spectrum was measured at 100 nm/s and all data were used as non-corrected values. The samples were excited at 470 nm, which remained constant in every measurement to ensure comparability. The bandwidth was set to 10 nm. After every 5 °C step, a spectrum was monitored (range: 500 – 800 nm). The spectra were normalized to 1 by dividing the spectra by the maximum value detected during one experimental data row. The samples were heated up to 140 °C in the fluorospectrometer. **PtL^a-12** was heated to only 100 °C since at this temperature the change in emission properties was already detectable and this compound had also a melting point (≈ 180 °C) higher than the tolerated temperature of the fluorospectrometer. The photographs were recorded using a Canon EOS 1100D using the same homebuilt heating stage. The samples were irradiated with a handheld 365 nm UV lamp.

The X-ray diffraction data was were recorded using a Stoe STADI P diffractometer (Cu Kα1 radiation: λ = 1.5406 Å, Ge 111 monochromator) in transmission geometry with a Dectris MYTHEN 1K detector. The samples were measured on flat sample holders over a 2θ -range of 4 – 40 ° with a scanning step of 3° and acquisition time of 120 s per step.

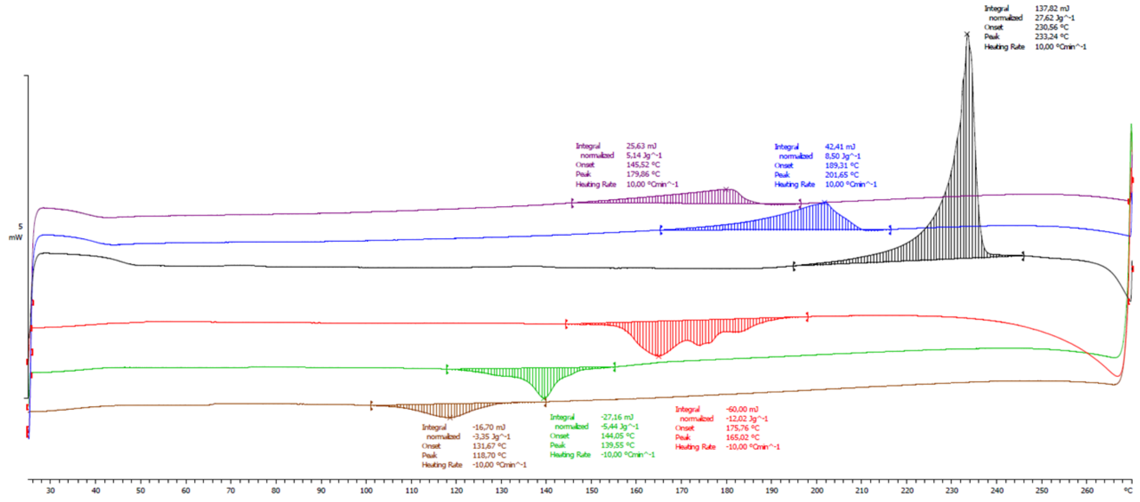

Figure S78. DSC profile of **PtL^a-6** on heating with a heating/cooling rate of 10 °C/min. First cycle (black and red curves), second cycle (blue and green curves), third cycle (purple and brown curves).

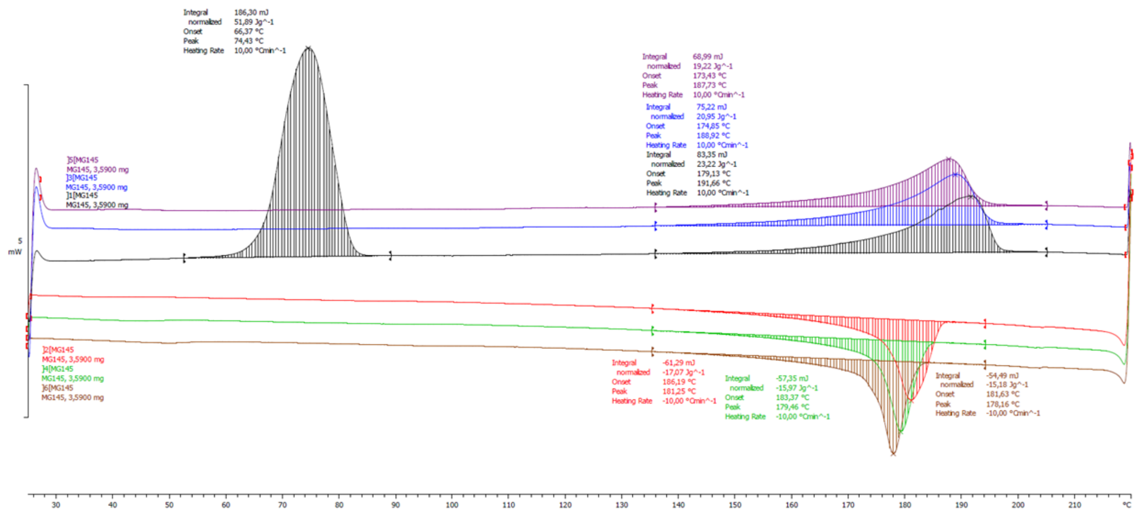

Figure S79. DSC profile of **PtL^a-12** on heating with a heating/cooling rate of 10 °C/min. First cycle (black and red curves), second cycle (blue and green curves), third cycle (purple and brown curves).

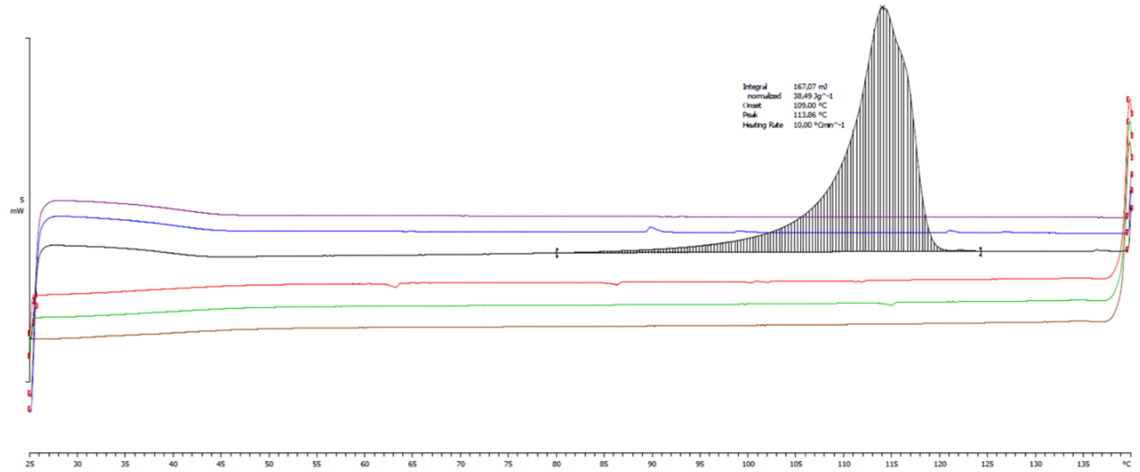

Figure S80. DSC profile of **PtL^b-6** on heating with a heating/cooling rate of 10 °C/min. First cycle (black and red curves), second cycle (blue and green curves), third cycle (purple and brown curves).

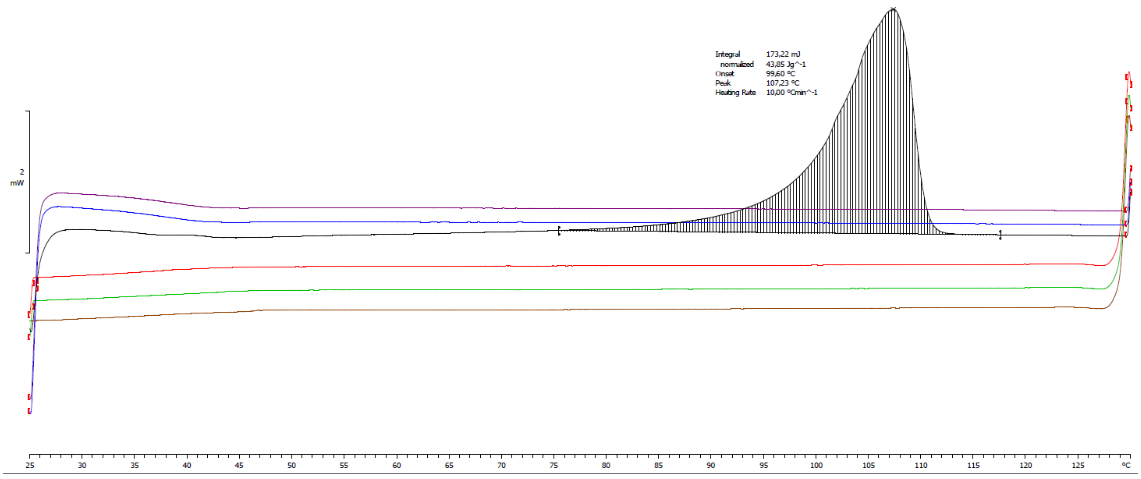

Figure S81. DSC profile of **PtL^b-12** on heating with a heating/cooling rate of 10 °C/min. First cycle (black and red curves), second cycle (blue and green curves), third cycle (purple and brown curves).

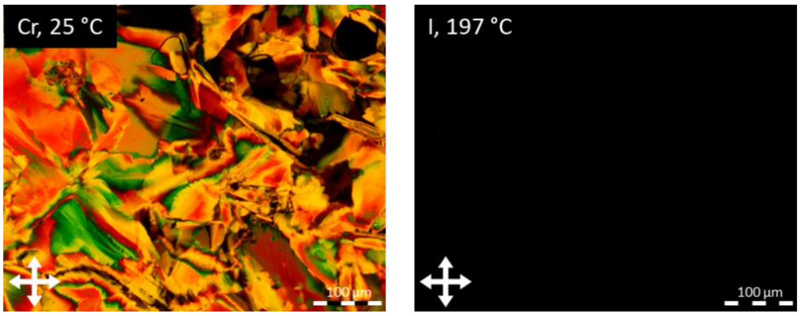

Figure S82. POM images of **PtL^a-12** in the crystalline phase (left) and in the isotropic phase (right) after melting on cooling.

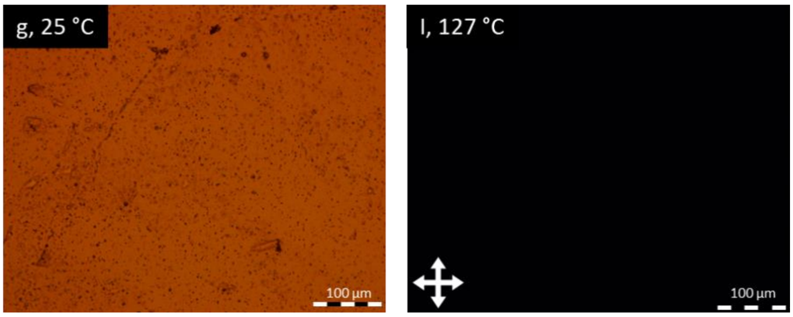

Figure S83. POM images of **PtL^b-6** in the glassy phase (left) and in the isotropic phase (right) after melting on cooling.

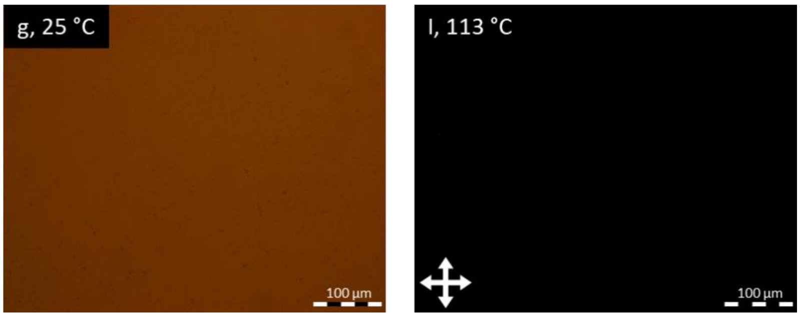

Figure S84. POM images of **PtL^b-12** in the glassy phase (left) and in the isotropic phase (right) after melting on cooling.

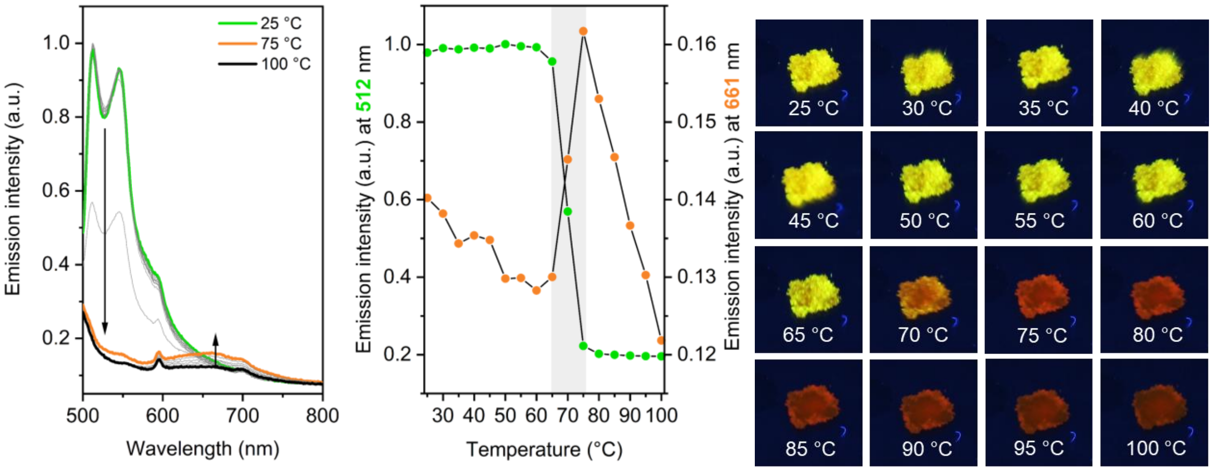

Figure S85. Left: Emission spectra of solid **PtLª-12** upon heating (λ_{exc} = 470 nm). Centre: Emission intensity at 512 nm and 661 nm as a function of the temperature. Right: Photographs of solid **PtLª-12** at different temperatures under UV excitation, λ_{exc} = 365 nm.

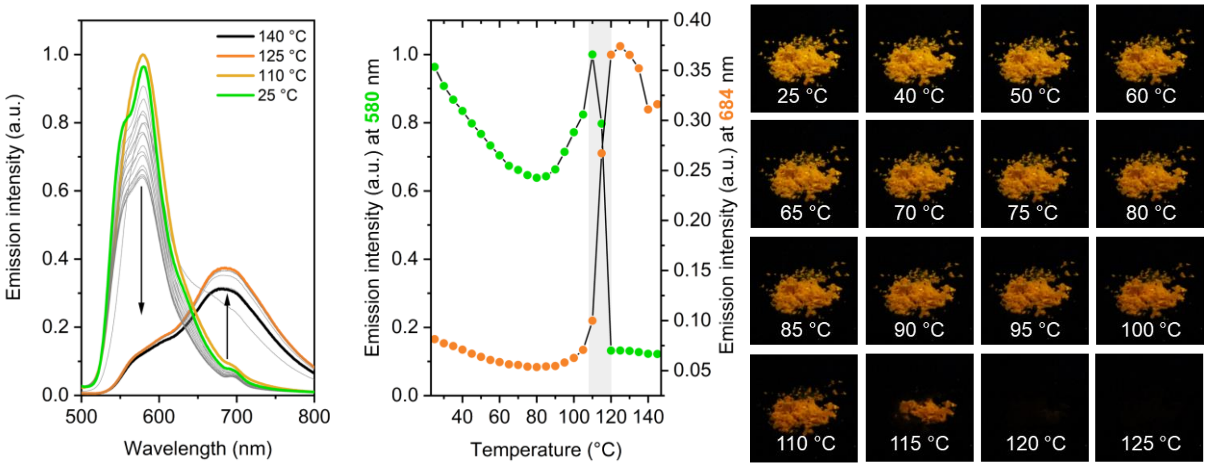

Figure S86. Left: Emission spectra of solid **PtLᵇ-6** upon heating (λ_{exc} = 470 nm). Centre: Emission intensity at 512 nm and 661 nm as a function of the temperature. Right: Photographs of solid **PtLª-12** at different temperatures under UV excitation, λ_{exc} = 365 nm.

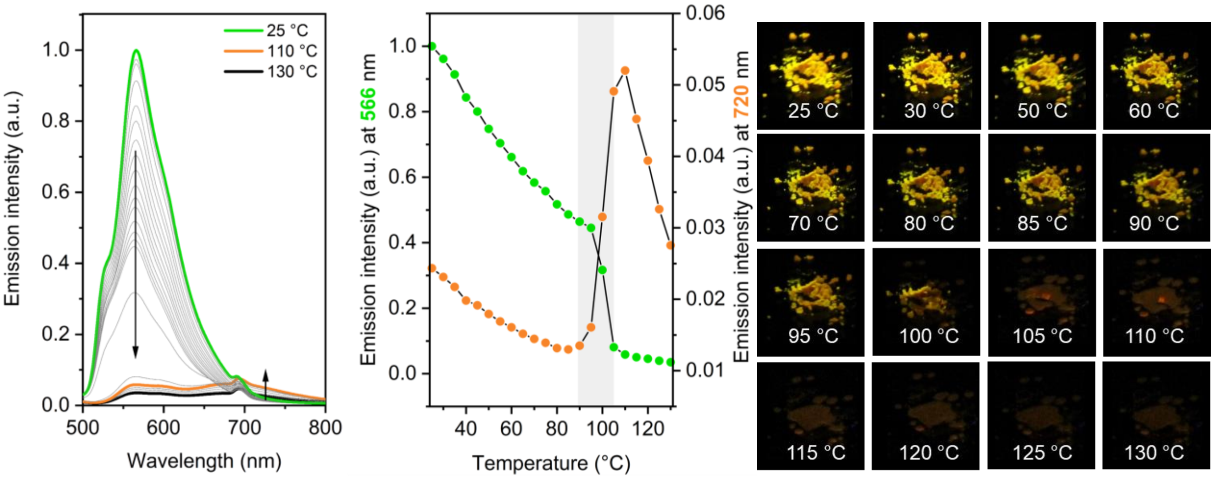

Figure S87. Left: Emission spectra of solid **PtLᵇ-12** upon heating (λ_{exc} = 470 nm). Centre: Emission intensity at 512 nm and 661 nm as a function of the temperature. Right: Photographs of solid **PtLª-12** at different temperatures under UV excitation, λ_{exc} = 365 nm.

5) References

1. Frisch, M.J.; Trucks, G.W.; Schlegel, H.B.; Scuseria, G.E.; Robb, M. a.; Cheeseman, J.R.; Scalmani, G.; Barone, V.; Petersson, G. a.; Nakatsuji, H.; et al. Gaussian 09 Revision D.01. 2009.
2. Adamo, C.; Barone, V. Toward Reliable Density Functional Methods without Adjustable Parameters: The PBE0 Model. *J. Chem. Phys.* **1999**, *110*, 6158–6170, doi:10.1063/1.478522.
3. Andrae, D.; Häußermann, U.; Dolg, M.; Stoll, H.; Preuß, H. Energy-Adjusted Ab Initio Pseudopotentials for the Second and Third Row Transition Elements. *Theoretica Chimica Acta* **1990**, *77*, 123–141, doi:10.1007/BF01114537.
4. Dunning, T.H.; Hay, P.J. *Modern Theoretical Chemistry*; Schaefer III, H.F., Ed.; Plenum: New York, 1977; ISBN 9781475708899.
5. Grimme, S.; Ehrlich, S.; Georigk, L. Software News and Updates Gabedit — A Graphical User Interface for Computational Chemistry Softwares. *Journal of computational chemistry* **2011**, *32*, 1456–1465, doi:10.1002/jcc.
6. Tomasi, J.; Mennucci, B.; Cammi, R. Quantum Mechanical Continuum Solvation Models. *Chem. Rev.* **2005**, *105*, 2999–3093, doi:10.1021/cr9904009.
7. Rappé, A.K.; Casewit, C.J.; Colwell, K.S.; Goddard III, W.A.; Skiff, W.M. UFF, a Full Periodic Table Force Field for Molecular Mechanics and Molecular Dynamics Simulations. *J. Am. Chem. Soc.* **1992**, *114*, 10024–10035, doi:10.1021/ja00051a040.
8. Plasser, F. TheoDORE: A Toolbox for a Detailed and Automated Analysis of Electronic Excited State Computations. *J. Chem. Phys.* **2020**, *152*, 084108, doi:10.1063/1.5143076.
9. Hanwell, M.D.; Curits, D.E.; Lonie, D.C.; Vandermeersch, T.; Zurek, E.; Hutchison, G.R. Avogadro: An Advanced Semantic Chemical Editor, Visualization, and Analysis Platform. *Journal of Cheminfomatics* **2012**, *4*, 1–17, doi:10.1016/j.aim.2014.05.019.
